# Synthesis of Novel Azo-Linked 5-Amino-Pyrazole-4-Carbonitrile Derivatives Using Tannic Acid–Functionalized Silica-Coated Fe_3_O_4_ Nanoparticles as a Novel, Green, and Magnetically Separable Catalyst

**DOI:** 10.3389/fchem.2021.724745

**Published:** 2021-11-12

**Authors:** Farhad Sedighi Pashaki, Mohammad Nikpassand

**Affiliations:** Department of Chemistry, Rasht Branch, Islamic Azad University, Rasht, Iran

**Keywords:** 5-amino-pyrazole-4-carbonitriles, Fe3O4@SiO2@Tannic acid, malononitrile, three-component reaction, tannic acid

## Abstract

Tannic acid–linked silica-coated Fe_3_O_4_ nanoparticles (Fe_3_O_4_@SiO_2_@Tannic acid) were prepared and characterized by transmission electron microscope (TEM), field emission scanning electron microscope (FE-SEM), X-ray powder diffraction (XRD), X-ray spectroscopy (EDX), vibrating sample magnetometry (VSM), and Fourier transform infrared (FT-IR) spectroscopy. Fe_3_O_4_@SiO_2_@Tannic acid supplies an environmentally friendly procedure for the synthesis of some novel 5-amino-pyrazole-4-carbonitriles through the three*-*component mechanochemical reactions of synthetized azo-linked aldehydes, malononitrile, and phenylhydrazine or *p*-tolylhydrazine. These compounds were produced in high yields and at short reaction times. The catalyst could be easily recovered and reused for six cycles with almost consistent activity. The structures of the synthesized 5-amino-pyrazole-4-carbonitrile compounds were confirmed by ^1^H NMR, ^13^C NMR, and FTIR spectra, and elemental analyses.

## Introduction

**FIGURE 1:**
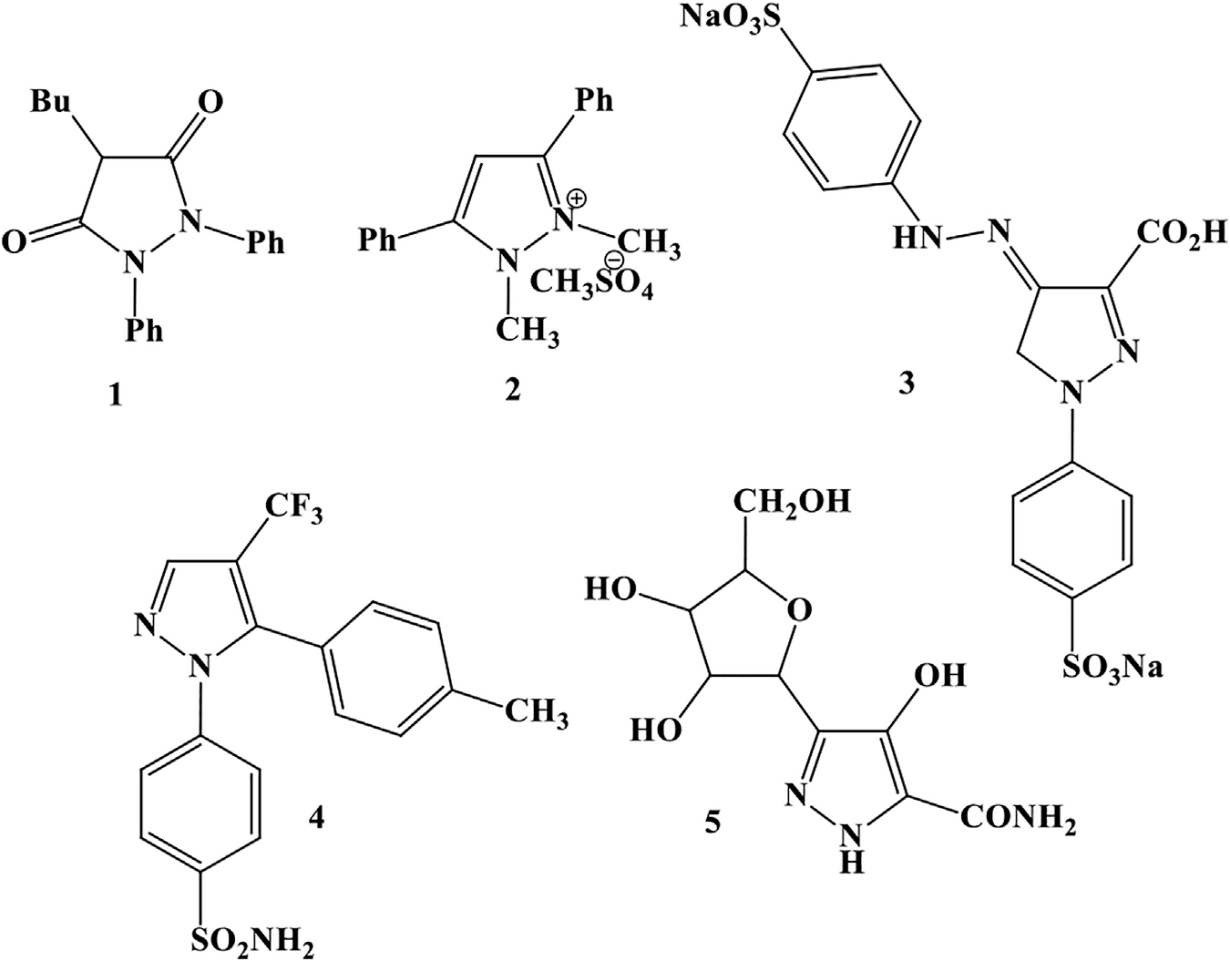
Some of the pyrazoles having biological properties.

One of the largest groups of heterocyclic compounds is five-membered rings with more than one heteroatom. One of the 5-membered rings with 2-heteroatom heterocycles is pyrazoles. Pyrazoles and their salts have numerous biological and pharmaceutical properties such as anti-inflammatory, sedative, hypnotic, fever-resistant, antifungal, and antibacterial. For example, 1) phenylbutazone acts as an anti-inflammatory agent, 2) diphenucate acts as a herbicide, 3) tartazine acts as a food coloring agent, 4) cecluxib acts as an anti-inflammatory agent, and 5) pyrazophine acts as a natural antibiotic and antitumor agent (Figure 1) (Bekhite and Aziem, 2004; Liu et al., 2008).

The synthesis of pyrazoles is specific because they are found in several different structures, including pyrazoles such as pyrazolotriazines (Karci and Demircah, 2008), pyrazolotetrazinones and pyrazolopyrimidines (Wu et al., 2006), and pyrazolo-pyrazines (El-Emary, 2006).

The simplest and most common method to synthesize pyrazoles is to use 1,3-dicarbonyl compounds or similar compounds such as acetals and imines with hydrazines (Bekhite and Aziem, 2004). In recent years, the synthesis of pyrazoles has become widespread. Saleh et al. (2012) prepared pyrazolo[3,4-*b*]pyridines from the reaction of phenyl sulfone synthon with *N*-phenyl benzene carbohydrazonyl chloride. The compounds produced in this study have anti-inflammatory properties. Trofimov et al. synthesized 3-amino-3-hydroxyalkyl-1-amino thiocarbonyl pyrazoles through the stereospecific cyclization reaction of 1, 2-acetylene-3-hydroxynitriles with thiosemicarbazide (Trofimov et al., 2008). Ortiz et al. (2006) prepared a new series of pyrazoles during the Diels–Alder reaction of 1,2,3-triazoles with diethyl acetylene dicarboxylate (DMAD) followed by pyrolysis of 3,4-dicarboxylate under solvent-free conditions.

Recently, different methods have been proposed to synthesize diverse pyrazole derivatives (Salaheldin et al., 2007). These include the synthesis of 4-substituted pyrazoles during the 1,3-dipolar ring-forming reaction between diazo compounds with triple bonds (Martin et al., 2006); the three-component reaction between aromatic aldehydes, malononitrile, and phenylhydrazine (Bhale et al., 2014); the Michael addition reaction using 2-methyl-3-nitrochrome as a starting material (Takagi et al., 1987); the preparation of oxoalkanenitrile or aminonitrile derivatives (Al-Qalaf et al., 2009), from a four-component reaction with aryl aldehydes, hydrazines, ethylacetoacetate, and malononitrile (Kiyani et al., 2013), and the reaction of enamines with hydroxylamine hydrochloride (Tominaga et al., 1990).

Some methods have also been reported for the synthesis of pyrazoles with azo bridges, such as the preparation of azo dyes from pyrazoles with a nitro group, such as 1-aryl-5-amino-4-cyano-pyrazole as the starting material (Towne et al., 1968), and the coupling reaction between pyrazolo[3,4-*d*]-pyrazine with phenol and 1-naphthol (Kasimogullari et al., 2010).

Chemistry is advancing toward new approaches that focus on the environment. Chemists try to use green techniques such as nontoxic solvents (such as water), solvent-free syntheses, cheap and available catalysts, and one-step multicomponent reactions; nanocatalysts play an essential role in green synthesis. Nano-dimensions provide tremendous advantages for using nanoparticles as catalysts. By reducing the particle size of the catalyst, there is an increase in contact surface with the reactants, and the catalytic power is improved, resulting in maximum efficiency with a small amount of catalyst. Another useful feature of nanocatalysts is their heterogeneity with high catalytic activity, so at the end of the reaction, the catalyst can be separated from the reaction mixture by smoothing and reused (Polshettiwar and Varma, 2010; Fihri et al., 2011; Fardood et al., 2017; Rezaei et al., 2017).

Compared to other nanoparticles, magnetite (Fe_3_O_4_), due to its unique magnetic properties (Xin et al., 2020), easy magnetic separation (Hamedi et al., 2018), low toxicity (Zhao et al., 2014), environmental compatibility (Eslahi et al., 2021), and chemically modifiable surface (Bai et al., 2013), has attracted scientists. Therefore, applications of these magnetic nanoparticles (MNPs) have been developed in drug delivery, cancer treatment, magnetic resonance imaging, tissue repairing, contrast agents, magnetic storage media, biosensing, magnetic inks for jet printing, and catalysis (Inaloo et al., 2020; Eslahi et al., 2021). However, MNPs easily aggregate in aqueous solutions due to their anisotropic dipolar attraction (Sardarian et al., 2019). Also, they are unstable in acidic environments and may be oxidized by air, which can alter their magnetic properties, reduce adsorption capacity, and limit the range of application (Pourjavadi et al., 2012). To overcome this limitation, stabilization of MNPs is performed. Magnetic shells, with core advantages and a wide range of shells, have attracted much attention in intensive research (Zhao et al., 2015).

This research is essential to design an efficient, green, and simple method to prepare 5-amino-pyrazole-4-carbonitriles. Herein, we report the mechanochemical synthesis of new 5-amino-pyrazole-4-carbonitriles with azo-linked aldehydes, malononitrile, and phenylhydrazine or *p*-tolylhydrazine at room temperature in the presence of tannic acid–functionalized silica-coated Fe_3_O_4_ nanoparticles (Fe_3_O_4_@SiO_2_@Tannic acid).

## Experimental

### Material and Method

Chemicals were purchased from Merck and Fluca and used as raw materials of standard purity. Melting temperatures were measured on electro-thermal 9100 devices and were uncorrected. For ultrasound reactions, the ultrasound apparatus Astra 3D (9.5 dm^3^, 45 kHz, 305 W) from TECNO-GAZ was used. FT-IR spectra were obtained on a Shimadzu FT-IR-8400S spectrometer. A Bruker DRX-500 Avance spectrometer was used to obtain the ^1^H NMR and ^13^C NMR spectra with DMSO-*d*_6_ as the solvent and TMS as internal standard. Elemental analyses were recorded on a Carlo-Erba EA1110CNNO-S analyzer. All mechanochemical reactions were carried out using a Retsch MM400 vibrational ball mill, equipped with Retsch 25 ml screw-top vessels, containing a 13.6-g stainless steel ball of 15 mm diameter unless otherwise stated. The operating frequency was set at 25 Hz for each experiment. The products were dried in a Carbolite PF60 oven set at 80°C.

### Synthesis of Silica-Coated Fe_3_O_4_ (Fe_3_O4@SiO_2_@Tannic Acid) MNPs

#### A. Synthesis of Fe_3_O_4_ MNPs and Fe_3_O_4_@SiO_2_-Cl MNPs

The Fe3O4 and Fe3O4@SiO2‐Cl MNPs were synthesized by the research group (Nikpassand et al., 2015; Nikpassand et al., 2017b).

#### B. Synthesis of Fe_3_O_4_@SiO_2_@Tannic Acid Nanoparticles

**FIGURE 2:**
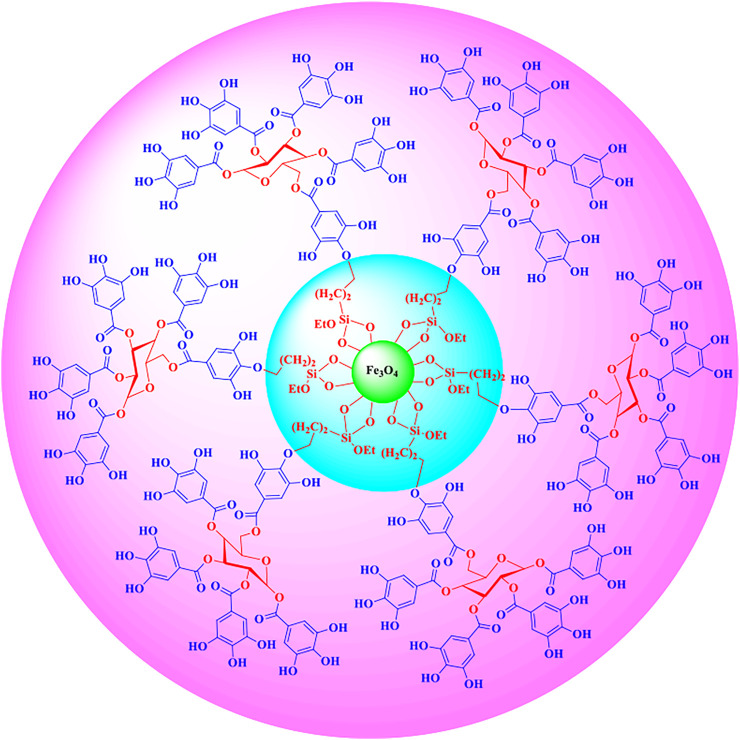
Structure of Fe_3_O_4_@SiO_2_@Tannic acid MNPs.

**FIGURE 3:**
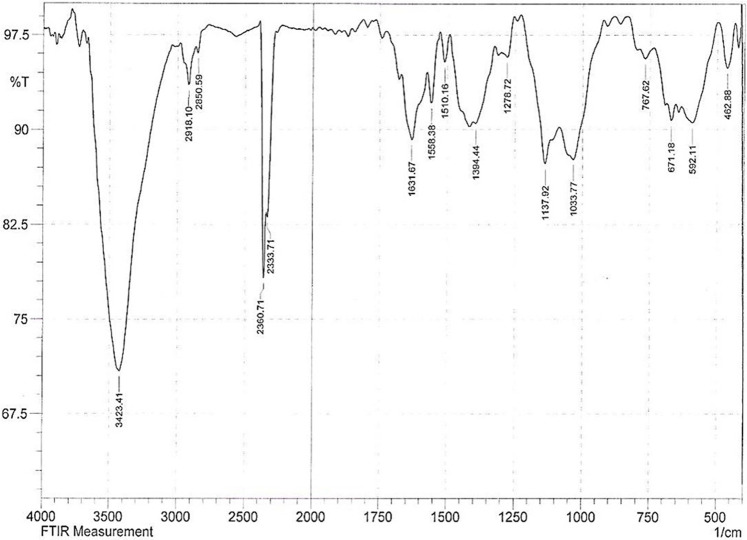
FT-IR spectra of synthesized Fe_3_O_4_@SiO_2_@Tannic acid MNPs.

**FIGURE 4:**
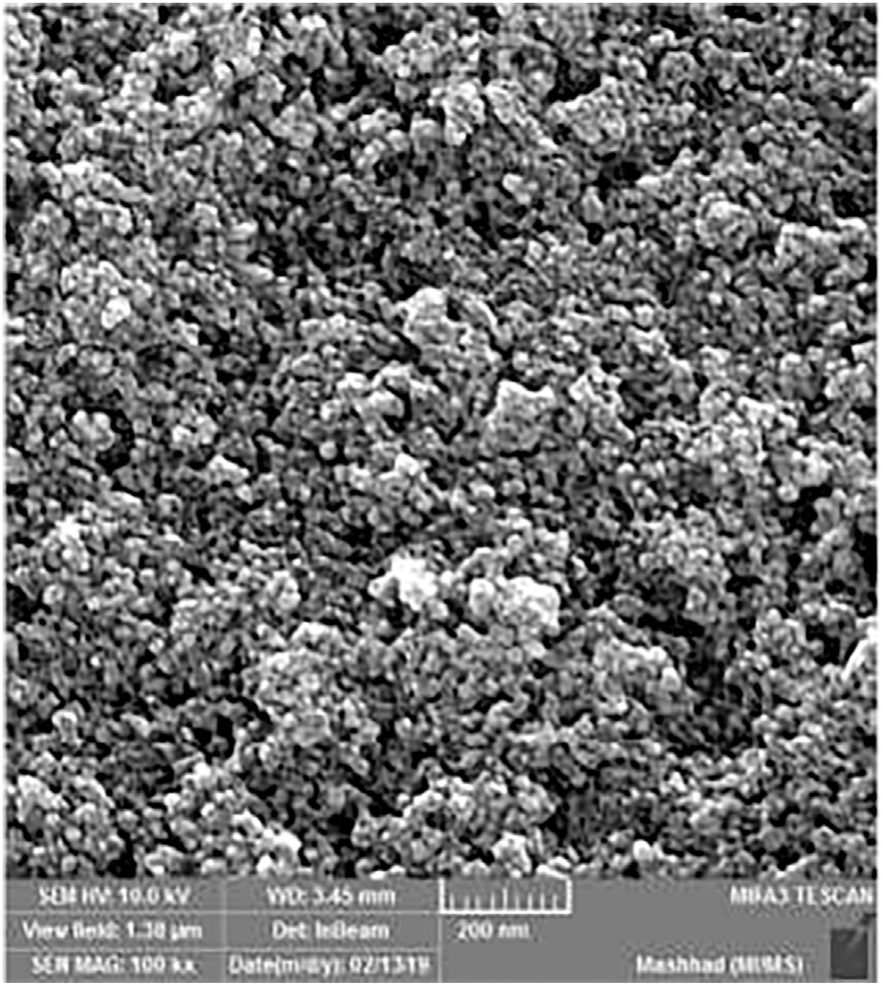
FE-SEM image of synthesized Fe_3_O_4_@SiO_2_@Tannic acid MNPs.

**FIGURE 5:**
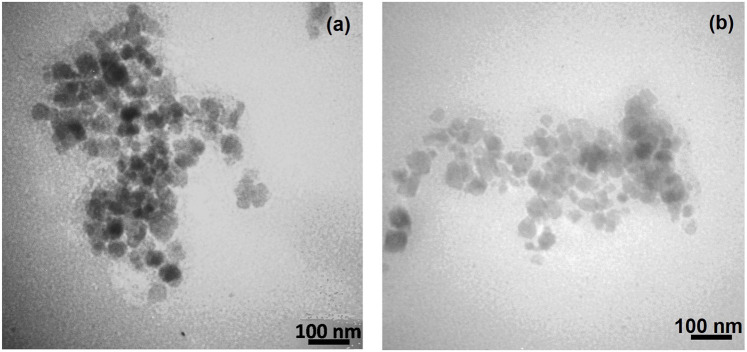
TEM images of synthesized Fe_3_O_4_@SiO_2_@Tannic acid MNPs: **(a)** after one cycle of reaction; **(b)** after six cycles of reaction.

**FIGURE 6:**
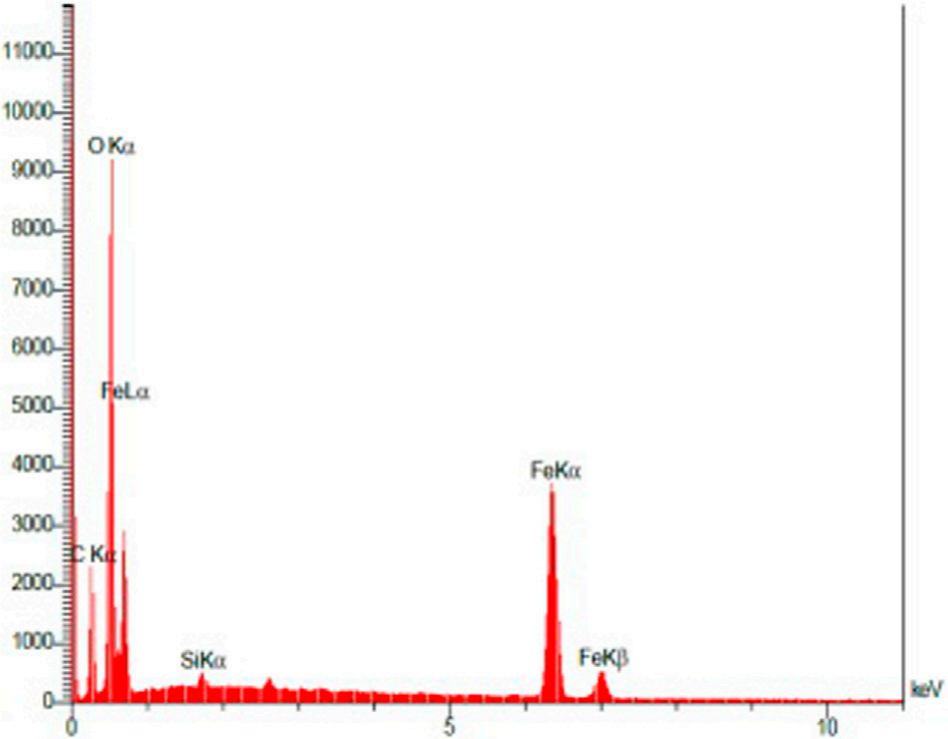
EDX image of synthesized Fe_3_O_4_@SiO_2_@Tannic acid MNPs.

**FIGURE 7:**
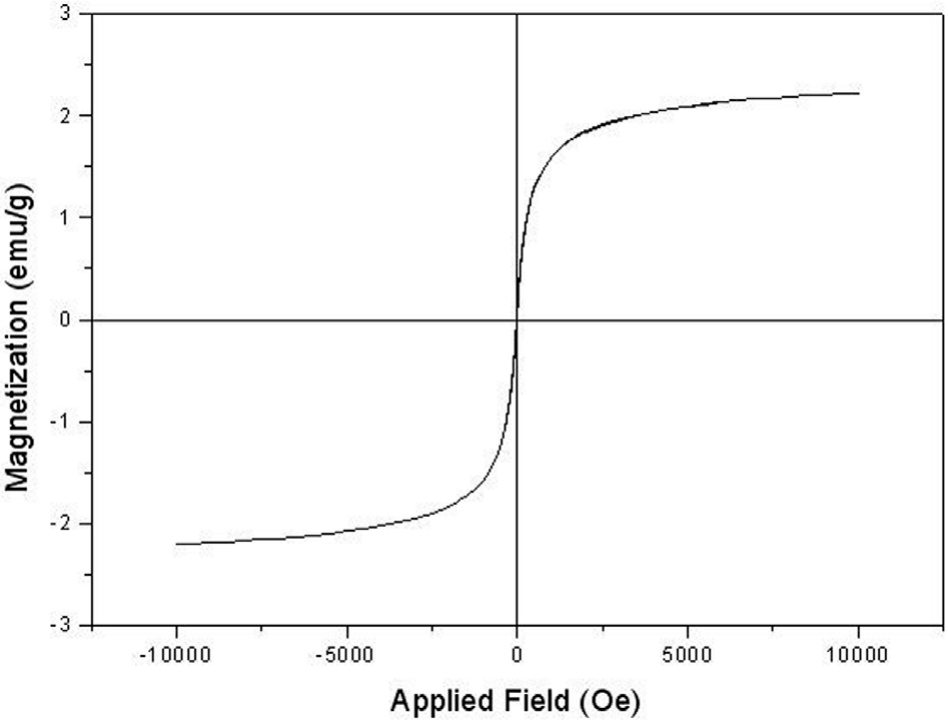
VSM image of synthesized Fe_3_O_4_@SiO_2_@Tannic acid MNPs.

**FIGURE 8:**
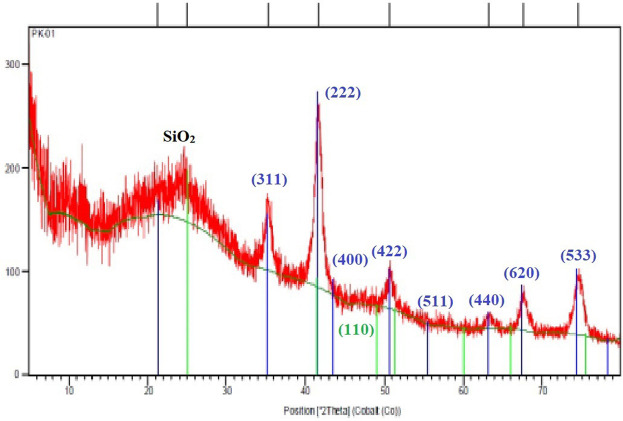
XRD image of synthesized Fe_3_O_4_@SiO_2_@Tannic acid MNPs.

Then Fe_3_O_4_@SiO_2_-Cl MNPs, tannic acid, and 15 ml of distilled water were stirred for 24 h. Then, 4 ml of 10% NaOH was added to the reaction mixture and stirred for 5 h. Then, 5 ml of triethylamine was added to the reaction mixture, and after stirring with a magnet, it was separated and incubated in the oven at 50°C for 24 h (Figure 1). The structure of nanocatalysts was confirmed by FT-IR, XRD, EDX, VSM, TEM, and SEM techniques (Figures 2–8).

### General Procedure for Preparation of 5-Amino-Pyrazole-4-Carbonitriles 4a-k

A mixture of synthesized azo-linked salicylaldehyde (1 mmol), phenylhydrazine (1 mmol, 0.108 g), or paratolylhydrazine (1 mmol, 0.120 g), malononitrile (1 mmol, 0.065 g), and 0.1 g Fe_3_O_4_@SiO_2_@Tannic acid was added to a Retsch 50-ml screw-top vessel equipped with a 20-mm stainless steel ball, and set to shake for the required reaction time (Table 2). Ball milling was performed at 20–25 Hz frequency at room temperature for the time given in Table 2. The progress of the reaction was investigated by thin-layer chromatography (TLC Silica gel 60 F₂₅₄, ethyl acetate: *n*-hexane 1: 2). After completion of the reaction, the resulting mixture was dissolved in hot ethanol (20 ml), and the catalyst was separated by a 1.4 T external magnet and washed with hot distilled water (5 ml) and ethanol (5 ml) two times. The resulting 3-pyrazolyl-4*H*-1,2,4-triazole was isolated and purified using column chromatography (silica gel 60 (0.063–0.200 mm); ethyl acetate: *n*-hexane 1: 2).

### Characterization Data

5-Amino-3-(5-((4-chlorophenyl)diazenyl)-2-hydroxyphenyl)-1-phenyl-1*H*-pyrazole-4-carbonitrile (4a); yellow solid, m.p.: 235–237°C, FT-IR (KBr, cm^−1^) ν_max_ 3,292 (N-H stretch), 3,064 (O-H stretch), 2,390 (C≡N stretch), 1,602 (N=N stretch), 1,566, 1,544, and 1,492 (C=C stretch), 1,276 (C-N stretch), 1,255 (C-O stretch), and 1,002 (C-Cl stretch) cm^−1^. ^1^H NMR (500 MHz, DMSO-d_6_): δ (ppm): 6.82 (t, J = 7.2 Hz, 1H), 7.04–7.11 (m, 4H), 7.28 (t, J = 7.5 Hz, 2H), 7.66 (dd, J = 6.3, 2.1 Hz, 2H), 7.80 (dd, J = 8.7, 2.4 Hz, 1H), 7.90 (dd, J = 6.3, 2.1 Hz, 2H), 8.25 (s, 2H, NH2), and 10.60 (s, 1H, OH) ppm. ^13^C NMR (125 MHz, DMSO-d_6_): δ (ppm): 112.3, 117.2, 119.6, 122.2, 122.3, 123.9, 124.4, 129.7, 129.9, 134.4, 135.1, 135.5, 142.6, 143.2, 145.1, 145.7, 151.1, and 159.2 ppm. Anal. calcd for C_22_H_15_ClN_6_O: C, 63.69; H, 3.64; and N, 20.26. Found: C, 63.72; H, 3.63; and N, 20.25.

5-Amino-3-(5-((2-chlorophenyl)diazenyl)-2-hydroxyphenyl)-1-phenyl-1*H*-pyrazole-4-carbonitrile (4b); brown solid, m.p.: 176–178°C, FT-IR (KBr, cm^−1^) ν_max_ 3,288 (N-H stretch), 3,060 (O-H stretch), 2,390 (C≡N stretch), 1,602 (N=N stretch), 1,566 and 1,494 (C=C stretch), 1,276 (C=N stretch), 1,255 (C-O stretch), and 1,029 (C-Cl stretch) cm^−1^. ^1^H NMR (500 MHz, DMSO-d_6_): δ (ppm): 4.50 (s, 2H, NH_2_), 6.80 (t, J = 7.8 Hz, 1H), 7.06 (d, J = 7.8 Hz, 2H), 7.16 (d, J = 7.8 Hz, 1H), 7.27 (t, J = 7.5 Hz, 2H), 7.36–7.55 (m, 2H), 7.68 (dd, J = 7.5, 1.2 Hz, 2H), 7.80 (dd, J = 7.8, 2.4 Hz, 1H), and 8.28 (s, 1H, OH) ppm. ^13^C NMR (125 MHz, DMSO-d_6_): δ (ppm): 112.3, 112.5, 117.3, 118.00, 119.6, 122.3, 123.2, 123.6, 128.4, 129.7, 130.2, 131.1, 132.2, 133.8, 135.0, 145.2, 146.1, 148.5, and 159.5 ppm. Anal. calcd for C_22_H_15_ClN_6_O: C, 63.69; H, 3.64; and N, 20.26. Found: C, 63.67; H, 3.65; and N, 20.28.

5-Amino-3-(2-hydroxy-5-((2-methyl-4-nitrophenyl)diazenyl)phenyl)-1-phenyl-1*H*-pyrazole-4-carbonitrile (4c); red solid, m.p.: 193–195°C, FT-IR (KBr, cm^−1^) ν_max_ 3,421 (N-H stretch), 3,330 (O-H stretch), 2,196 (C≡N stretch), 1,683 (N=N stretch), 1,600 and 1,564 (C=C stretch), 1,519 (NO_2_ stretch), 1,492 (C=C stretch), 1,334 (NO_2_ stretch), and 1,255 (C-O stretch) cm^−1^. ^1^H NMR (500 MHz, DMSO-d_6_): δ (ppm): 2.42 (s, 3H, CH_3_), 6.81 (t, J = 7.2 Hz, 1H), 7.04–7.10 (m, 3H), 7.27 (t, J = 8.1 Hz, 2H), 7.68 (d, J = 9.0 Hz, 1H), 7.80 (dd, J = 9.0, 2.7 Hz, 1H), 8.12 (dd, J = 8.7, 2.7 Hz, 1H), 8.22 (s, 1H), 8.26 (d, J = 2.1 Hz, 1H), and 10.61 (s, 1H, OH) ppm. ^13^C NMR (125 MHz, DMSO-d_6_): δ (ppm): 25.51, 112.3, 117.0, 117.3, 118.4, 119.6, 122.3, 122.5, 122.9, 123.7, 123.8, 126.6, 129.7, 134.9, 138.5, 144.5, 145.1, 146.4, 148.0, 154.0, 154.0, and 159.9 ppm. Anal. calcd for C_23_H_17_N_7_O_3_: C, 62.87; H, 3.90; and N, 22.31. Found: C, 62.87; H, 3.89; and N, 22.33.

5-Amino-3-(2-hydroxy-5-((4-nitrophenyl)diazenyl)phenyl)-1-phenyl-1*H*-pyrazole-4-carbonitrile (4d); red solid, m.p.: 149–151°C, FT-IR (KBr, cm^−1^) ν_max_ 3,461 (N-H stretch), 3,307 (N-H stretch), 3,037 (O-H stretch), 2,189 (C≡N stretch), 1,662 (N=N stretch), 1,641, 1,598, and 1,566 (C=C stretch), 1,515 and 1,340 (NO_2_ stretch), and 1,251 (C-O stretch) cm^−1^. ^1^H NMR (500 MHz, DMSO-d_6_): δ (ppm): 6.78–7.30 (m, 6H), 7.49–7.54 (m, 1H), 7.96–8.02 (m, 2H), 8.16 (s, 1H), 8.23–8.25 (m, 1H), 8.34–8.39 (m, 1H), and 10.27 (s, 1H, OH) ppm. ^13^C NMR (125 MHz, DMSO-d_6_): δ (ppm): 112.1, 112.3, 116.3, 117.6, 119.4, 119.8, 119.9, 120.8, 123.5, 125.3, 127.8, 129.6, 129.7, 136.8, 137.8, 145.0, 145.1, and 156.1 ppm. Anal. calcd for C_22_H_15_N_7_O_3_: C, 62.11; H, 3.55; and N, 23.05. Found: C, 62.09; H, 3.57; and N, 23.06.

5-Amino-3-(5-((4-bromophenyl)diazenyl)-2-hydroxyphenyl)-1-phenyl-1*H*-pyrazole-4-carbonitrile (4e); off-white solid, m.p.: 218–220°C, FT-IR (KBr, cm^−1^) ν_max_ 3,433 (N-H stretch), 3,321 (N-H stretch), 3,051 (O-H stretch), 2,190 (C≡N stretch), 1,683 (N=N stretch), 1,658 and 1,568 (C=C stretch), 1,255 (C-O stretch), and 1,002 (C-Br stretch) cm^−1^. ^1^H NMR (500 MHz, DMSO-d_6_): δ (ppm): 6.79 (t, J = 6.9 Hz, 1H), 7.04–7.11 (m, 2H), 7.28 (t, J = 5.8 Hz, 2H), 7.76–7.84 (m, 5H), 8.24–8.26 (m, 2H), and 10.60 (s, 1H, OH) ppm. ^13^C NMR (125 MHz, DMSO-d_6_): δ (ppm): 112.1, 112.3, 117.2, 119.6, 122.2, 122.4, 123.9, 124.6, 124.8, 129.7, 132.8, 133.1, 135.3, 145.1, 145.7, 151.4, 156.1, and 159.2 ppm. Anal. calcd for C_22_H_15_BrN_6_O: C, 57.53; H, 3.29; and N, 18.30. Found: C, 57.55; H, 3.30; and N, 18.29.

5-Amino-3-(2-hydroxy-5-((4-methoxyphenyl)diazenyl)phenyl)-1-phenyl-1*H*-pyrazole-4-carbonitrile (4f); yellow solid, m.p.: 184–186°C, FT-IR (KBr, cm^−1^) ν_max_ 3,419 (N-H stretch), 3,315 (O-H stretch), 2,189 (C≡N stretch), 1,686 (N=N stretch), 1,647, 1,600, and 1,577 (C=C stretch), and 1,244 (C-O stretch) cm^−1^. ^1^H NMR (500 MHz, DMSO-d_6_): δ (ppm): 3.85 (s, 3H, CH_3_O), 6.82 (t, J = 7.2 Hz, 1H), 7.09–7.20 (m, 5H), 7.29 (t, J = 7.6 Hz, 2H), 7.71–7.77 (m, 1H), 7.88 (d, J = 8.4 Hz, 2H), 8.19–8.29 (m, 1H), and 10.61 (s, 1H, OH) ppm. ^13^C NMR (125 MHz, DMSO-d_6_): δ (ppm): 55.9, 112.3, 114.9, 115.1, 115.6, 117.1, 119.6, 121.8, 122.1, 123.4, 124.1, 124.6, 129.7, 130.7, 136.1, 145.1, 145.8, 146.7, and 158.4 ppm. Anal. calcd for C_23_H_18_N_6_O_2_: C, 67.31; H, 4.42; and N, 20.48. Found: C, 67.33; H, 4.41; and N, 20.50.

5-Amino-3-(5-((4-chlorophenyl)diazenyl)-2-hydroxyphenyl)-1-(*p*-tolyl)-1*H*-pyrazole-4-carbonitrile (4 g); brown solid, m.p.: 234–236°C, FT-IR (KBr, cm^−1^) ν_max_ 3,292 (N-H stretch), 3,064 (O-H stretch), 2,339 (C≡N stretch), 1,602 (N=N stretch), 1,1566, 1,544 and 1,452 (C=C stretch), 1,276 (C-N stretch), 1,255 (C-O stretch), and 1,002 (C-Cl stretch) cm^−1^. ^1^H NMR (500 MHz, DMSO-d_6_): δ (ppm): 2.25 (s, 3H, CH_3_), 6.94 (d, J = 8.4 Hz, 2H), 7.08–7.11 (m, 4H), 7.66 (dt, J = 8.4, 3.0 Hz, 2H), 7.78 (dd, J = 8.6, 2.4 Hz, 1H), 7.91 (dt, J = 8.4, 3.0 Hz, 2H), 8.21–8.22 (s, 2H), and 10.49 (s, 1H, OH) ppm. ^13^C NMR (125 MHz, DMSO-d_6_): δ (ppm): 20.7, 112.4, 117.1, 122.3, 122.4, 123.3, 123.7, 124.3, 128.2, 129.9, 130.2, 132.5, 134.7, 135.5, 137.6, 142.8, 145.7, 151.1, and 159.1 ppm. Anal. calcd for C_23_H_17_ClN_6_O: C, 64.41; H, 4.00; and N, 19.60. Found: C, 64.39; H, 3.99; and N, 19.63.

5-Amino-3-(2-hydroxy-5-((2-methyl-4-nitrophenyl)diazenyl)phenyl)-1-(*p*-tolyl)-1*H*-pyrazole-4-carbonitrile (4h); brown solid, m.p.: 190–192°C, FT-IR (KBr, cm^−1^) ν_max_ 3,423 and 3,328 (N-H stretch), 3,218 (O-H stretch), 2,268 (C≡N stretch), 1,681 (N=N stretch), 1,664 (C=N stretch), 1,618, 1,583, and 1,564 (C=C stretch), 1,515 and 1,340 (NO_2_ stretch), and 1,255 (C-O stretch) cm^−1^.^1^H NMR (500 MHz, DMSO-d_6_): δ (ppm): 2.33 (s, 3H, CH_3_), 2.74 (s, 3H, CH_3_), 4.51 (s, 2H, NH_2_), 6.94 (d, J = 8.4 Hz, 2H), 7.06–7.18 (m, 3H), 7.66 (d, J = 8.4 Hz, 1H), 7.78 (dd, J = 8.7 2.4 Hz, 1H), 8.15 (dd, J = 8.7, 2.7 Hz, 1H), 8.20 (s, 1H), 8.24 (d, J = 2.4 Hz, 1H), and 10.19 (s, 1H, OH) ppm. ^13^C NMR (125 MHz, DMSO-d_6_): δ (ppm): 17.4, 20.6, 112.4, 112.5, 112.7, 117.0, 117.3, 122.4, 122.5, 123.4, 123.7, 124.4, 126.6, 128.2, 130.1, 134.4, 138.5, 142.9, 146.4, 148.0, 154.1, and 159.9 ppm. Anal. calcd for C_24_H_19_N_7_O_3_: C, 63.57; H, 4.22; and N, 21.62. Found: C, 63.59; H, 4.20; and N, 21.63.

5-Amino-3-(5-((2-chlorophenyl)diazenyl)-2-hydroxyphenyl)-1-(*p*-tolyl)-1*H*-pyrazole-4-carbonitrile (4i); brown solid, m.p.: 172–174°C, FT-IR (KBr, cm^−1^) ν_max_ 3,218 (N-H stretch), 3,035 (O-H stretch), 2,290 (C≡N stretch), 1,667 (N=N stretch), 1,664, 1,612, and 1,581 (C=C stretch), 1,247 (C-O stretch), and 1,056 (C-Cl stretch) cm^−1^. ^1^H NMR (500 MHz, DMSO-d_6_): δ (ppm): 2.23 (s, 3H, CH_3_), 7.53 (s, 2H, NH2), 6.92–7.10 (m, 5H), 7.46–7.56 (m, 3H), 7.68–7.78 (m, 2H), 8.22 (s, 1H), and 10.23 (s, 1H, OH) ppm. ^13^C NMR (125 MHz, DMSO-d_6_): δ (ppm): 20.7, 112.3, 112.5, 115.3, 118.0, 122.4, 123.6, 128.2, 128.4, 128.5, 129.7, 130.1, 131.0, 131.1, 133.7, 134.3, 134.5, 142.9, 146.1, 148.1, and 159.5 ppm. Anal. calcd for C_23_H_17_ClN_6_O: C, 64.41; H, 4.00; and N, 19.60. Found: C, 64.39; H, 4.01; and N, 19.59.

5-Amino-3-(2-hydroxy-5-((4-nitrophenyl)diazenyl)phenyl)-1-(*p*-tolyl)-1*H*-pyrazole-4-carbonitrile (4j); brown solid, m.p.: 131–133°C, FT-IR (KBr, cm^−1^) ν_max_ 3,477 and 3,309 (N-H stretch), 3,045 (O-H stretch), 2,360 (C≡N stretch), 1,614 (N=N stretch), 1,589, 1,564, and 1,488 (C=C stretch), 1,402 (NO_2_ stretch), and 1,271 (C-O stretch) cm^−1^. ^1^H NMR (500 MHz, DMSO-d_6_): δ (ppm): 12.23 (s, 3H, CH_3_), 4.52 (s, 2H, NH_2_), 6.87–6.98 (m, 1H),7.05–7.09 (m, 1H), 7.14–7.20 (m, 1H), 7.37 (s, 1H), 7.49–7.54 (m, 2H), 7.80–7.84 (dd, J = 8.6, 1.8 Hz, 1H), 8.05 (d, J = 8.6 Hz, 1H), 8.14 (s, 1H), 8.23–8.27 (m, 1H), 8.40 (d, J = 8.6 Hz, 1H), and 8.92 (s, 1H, OH) ppm. ^13^C NMR (125 MHz, DMSO-d_6_): δ (ppm): 20.7, 112.2, 112.4, 112.5, 116.3, 119.7, 120.9, 122.6, 123.5, 125.4, 127.7, 128.0, 129.4, 130.1, 137.3, 142.9, 145.9, 148.3, and 156.1 ppm. Anal. calcd for C_23_H_17_N_7_O_3_: C, 62.87; H, 3.90; and N, 22.31. Found: C, 62.85; H, 3.88; and N, 22.33.

5-Amino-3-(5-((4-bromophenyl)diazenyl)-2-hydroxyphenyl)-1-(*p*-tolyl)-1*H*-pyrazole-4-carbonitrile (4k); yellow solid, m.p.: 180–183°C, FT-IR (KBr, cm^−1^) ν_max_ 3,218 (N-H stretch), 3,037 (O-H stretch), 2,366 (C≡N stretch), 1,668 (N=N stretch), 1,573, 1,517, and 1,483 (C=C stretch), 1,280 (C-O stretch), and 1,006 (C-Br stretch) cm^−1^.^1^H NMR (500 MHz, DMSO-d_6_): δ (ppm): 2.22 (s, 3H, CH_3_), 4.56 (s, 2H, NH_2_), 6.93–6.98 (m, 3H),7.05–7.09 (m, 3H), 7.14 (d, J = 8.7 Hz, 1H), 7.27–7.81 (m, 2H), 8.17 (d, J = 2.4 Hz, 1H), and 8.26 (s, 1H) ppm. ^13^C NMR (125 MHz, DMSO-d_6_): δ (ppm): 20.6, 112.4, 112.5, 115.3, 117.2, 122.3, 124.5, 126.1, 128.1, 129.7, 130.1, 130.8, 132.8, 134.6, 142.9, 143.6, 145.6, 151.4, and 159.3 ppm. Anal. calcd for C_23_H_17_BrN_6_O: C, 58.36; H, 3.62; and N, 17.76. Found: C, 58.34; H, 3.63; and N, 17.77.

## Results and Discussion

### Synthesis and Characterization of Fe_3_O_4_@SiO_2_@Tannic Acid

In continuation of our research for the green synthesis of organic compounds (Nikpassand et al., 2012; Nikpassand et al., 2016; Nikpassand and Pirdelzendeh, 2016; Nikpassand et al., 2017; Aghazadeh and Nikpassand, 2019; Nikpassand et al., 2018; Masoumi Shahi et al., 2019; Zare Fekri et al., 2019; Nikpassand, 2020), herein we wish to report the synthesis of novel azo-linked 5-amino-pyrazole-4-carbonitriles catalyzed by tannic acid–functionalized silica-coated Fe_3_O_4_ nanoparticles (Fe_3_O_4_@SiO_2_@Tannic acid).

The structure of the Fe_3_O_4_@SiO_2_@Tannic acid nanoparticles is synthesized in three steps from existing commercial materials, as shown in Figure 2. Fe_3_O_4_@SiO_2_ core-shell structures were sequentially treated with 3-chloropropyltrimethoxysilane. Next, it was treated with tannic acid to produce Fe_3_O_4_@SiO_2_@Tannic acid (Figure 2).

FT-IR spectroscopy of Fe_3_O_4_@SiO_2_@Tannic acid MNPs was performed to identify the functional groups of the synthesized nanoparticles. The strong stretching bond at 3,409 cm^−1^ is related to the O-H stretching vibrations of the phenolic moiety of the nano-catalyst, and C=O stretching bands of carboxylic acid were shown at 1704 cm^−1^, which confirms the presence of tannic acid in the structure of nanoparticles. The bonds at 1,620, 1,506, and 1,453 cm^−1^ are assigned to the C=C stretching vibrations of the aromatic moiety. Also, vibrations of Si-O-Si bonds in the SiO_2_ shell were observed at 1,116 and 906 cm^−1^ (Figure 3).

The size and morphology of the Fe_3_O_4_@SiO_2_@Tannic acid MNPs were studied using transmission electron microscopy and field emission scanning electron microscopy (Figures 4, 5). The transmission electron microscope (TEM) and field emission scanning electron microscope (FE-SEM) images in Figures 4, 5 show that the Fe_3_O_4_@SiO_2_@Tannic acid nanoparticles have an almost spherical morphology with a particle size of 10–20 nm. In addition, TEM images show aggregation that confirms the successful bonding of tannic acid with magnetic nanoparticles (Figures 4, 5).

The data from the energy-dispersive X-ray spectroscopy (EDX) analysis of the synthesized Fe_3_O_4_@SiO_2_@Tannic acid MNPs confirm the nanoparticle structure. Thus, the presence of Fe (21.35 w/w %), O (52.38 w/w %), Si (0.36 w/w %), and C (25.92 w/w %) atoms in the structure proves the presence of Fe_3_O_4_ core in the structure of Fe_3_O_4_@SiO_2_@Tannic acid MNPs (Figure 6).

The VSM plot of the Fe_3_O_4_@SiO_2_@Tannic acid MNPs is presented in Figure 7. As can be seen, the saturation magnetization of the MNPs is smaller than that of the pure Fe_3_O_4_. VSM was measured during solid sampling at the tip of a vibrating rod at room temperature and analyzed in an applied magnetic field from −10 to 10 kOe (Figure 7).

XRD analysis of the Fe_3_O_4_@SiO_2_@Tannic acid catalyst in contact with pure Fe_3_O_4_ confirms the formation of Fe_3_O_4_ MNPs. This pattern shows characteristic peaks at 2θ = 21.3, 25.1, 35.2, 41.5, 43.4, 49.0, 50.4, 51.3, 55.7, 60.0, 63.2, 66.0, 67.4, 74.3, 75.5, and 78.3. These peaks indicate the pure face-centered cubic structure of Fe_3_O_4_, and the broad peak at 10–30° is related to Fe_3_O_4_ covered by SiO_2_ (Figure 8).

### Catalytic Application

**SCHEME 1:**
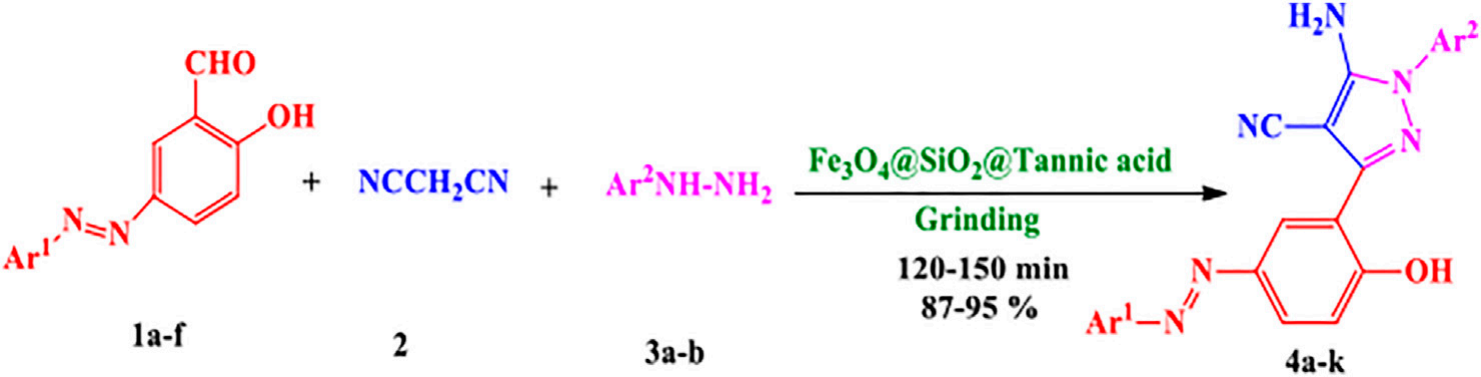
Synthesis of novel 5-amino-pyrazole-4-carbonitriles using Fe_3_O_4_@SiO_2_@Tannic acid.

To evaluate the catalytic capability of the synthesized heterogeneous catalyst (Fe_3_O_4_@SiO_2_@Tannic acid) in organic reactions, we chose to examine its activity in a one-pot mechanochemical reaction between synthetized azo-linked aldehydes, malononitrile, and phenylhydrazine or *p*-tolylhydrazine (Scheme 1).

Initially, 5-((4-chlorophenyl)diazenyl)-2-hydroxybenzaldehyde **1a** (1 mmol, 0.260 g), malononitrile **2** (1 mmol, 0.065 g), phenylhydrazine **3a** (1 mmol, 0.108 g), and 0.1 g of Fe_3_O_4_@SiO_2_@Tannic acid were employed to produce 5-amino-3-(5-((4-chlorophenyl)diazenyl)-2-hydroxyphenyl)-1-phenyl-*1H*-pyrazole-4-carbonitrile (**4a**), and the effect of various factors such as the type of catalyst, its relative amount of raw material, and reaction temperature on this sample reaction was investigated (Tables 1–3).

**TABLE 1:** Influence of catalyst types on reaction time and efficiency in the synthesis of **4a**.

Entry	Catalyst	Yield (%)	Time (h)
^a^1	−	55	**24**
^a^2	Nano-SiO_2_	63	**6**
^a^3	K10	66	**12**
^a^4	Nano-Fe_3_O_4_	72	**6**
^a^5	Fe_3_O_4_@SiO_2_@Tannic acid	91	**2**
^b^6	[BBIM]Br	78	**5**
^b^7	[BBIM]HSO_4_	83	**5**

a Reaction conditions: 5-((4-chlorophenyl)diazenyl)-2-hydroxybenzaldehyde **1a** (1 mmol), malononitrile **2** (1 mmol), and phenylhydrazine **3a** (1 mmol) were used under solvent-free conditions.

b 2 ml of ionic liquid was used in entries 6 and 7.

**TABLE 2:** Investigation of the amount of catalyst used in the synthesis of **4a**.

Entry	Amount of catalyst (g)	Yield (%)	Time (h)
1	0.05	78	6
2	0.1	91	2
3	0.2	91	2

**TABLE 3:** Synthesis of 5-amino-pyrazole-4-carbonitriles using Fe_3_O_4_@SiO_2_@Tannic acid.

Entry	Product	Structure	Time (min)	Yield (%)^a^
1	**4a**	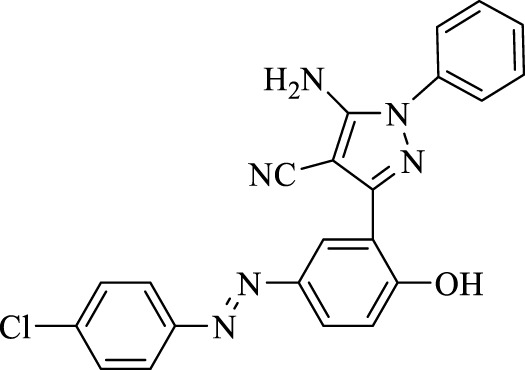	120	91
2	**4b**	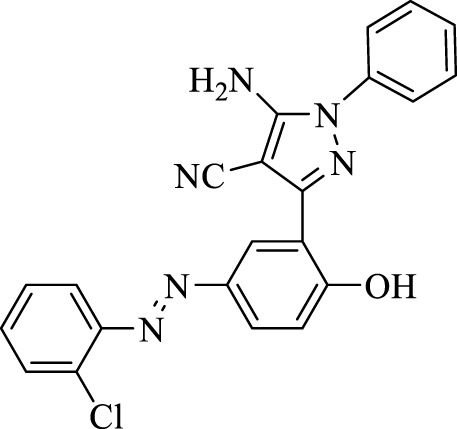	120	87
3	**4c**	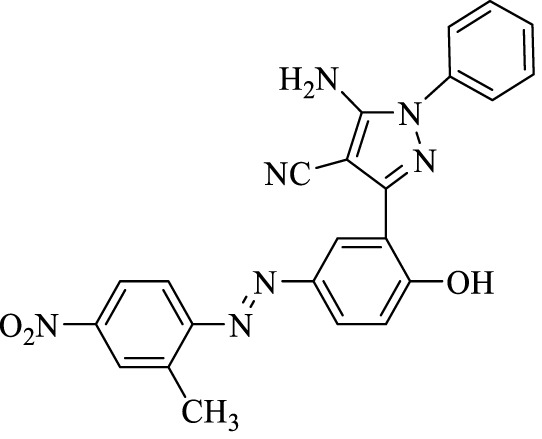	150	89
4	**4d**	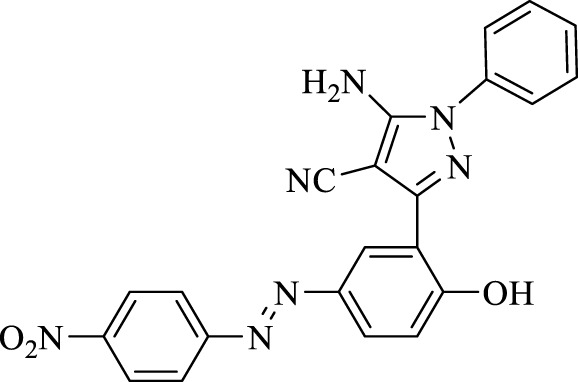	120	95
5	**4e**	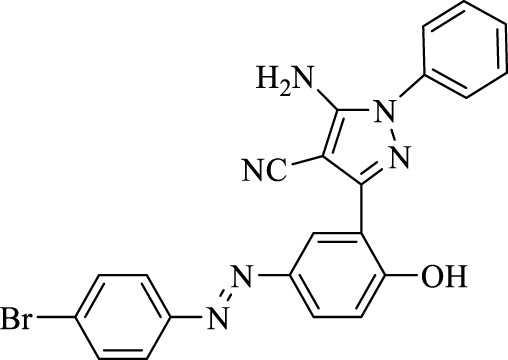	120	93
6	**4f**	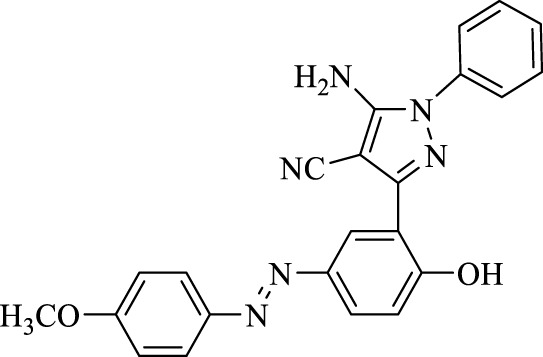	150	90
7	**4g**	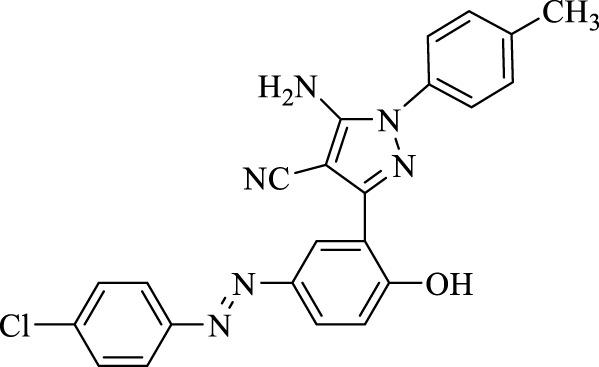	120	92
8	**4h**	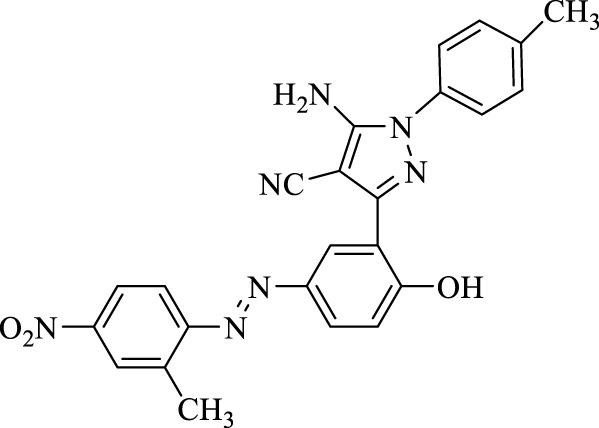	150	89
9	**4i**	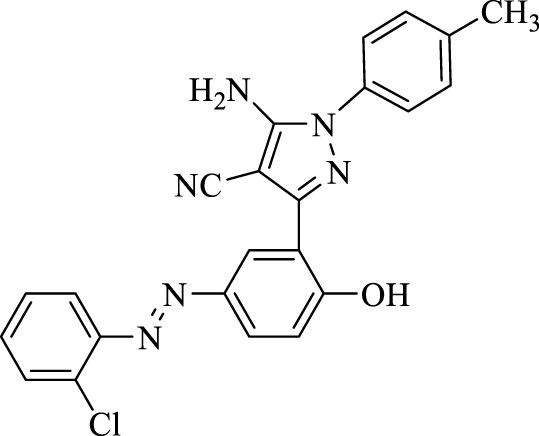	150	89
10	**4j**	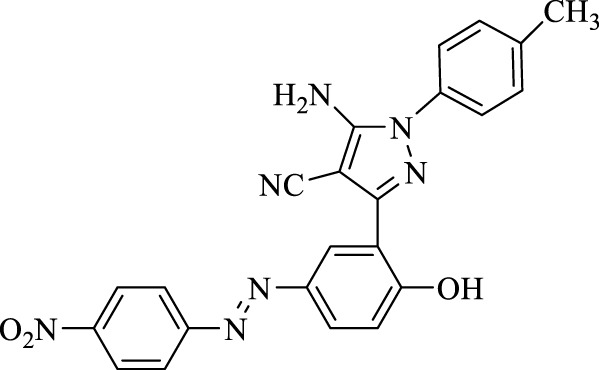	120	94
11	**4k**	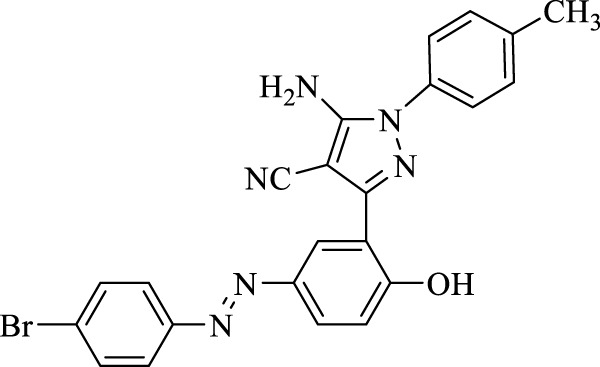	120	93

a Yields based upon the starting azo-linked aldehydes.

#### Effect of Catalyst Type

To find the appropriate catalyst to synthesize the derivatives of azo-linked 5-amino-pyrazole-4-carbonitriles, the reaction of 5-((4-chlorophenyl)diazenyl)-2-hydroxybenzaldehyde **1a** (1 mmol, 0.260 g), malononitrile **2** (1 mmol, 0.065 g), and phenylhydrazine **3a** (1 mmol, 0.108 g) in the presence of 0.1 g of available catalysts at 80°C under different conditions was used, and the efficiency and reaction rates were compared (Table 1).

#### Effect of Fe_3_O_4_@SiO_2_@Tannic Acid Catalyst Value

The synthesis of product **4a** with different amounts of Fe_3_O_4_@SiO_2_@Tannic acid at room temperature was investigated, and it was found that 0.1 g of the desired catalyst per 1 mmol of substrate gave a better yield in a shorter reaction time (Table 2).

To present the efficiency and generality of the mechanochemical reaction, various azo-linked aldehydes, malononitrile, and phenylhydrazine or *p*-tolylhydrazine were reacted in the presence of Fe_3_O_4_@SiO_2_@Tannic acid at room temperature (Scheme 1 and Table 3).

The recyclability and reusability of a catalyst were studied in the model one-pot mechanochemical reaction between various azo-linked aldehydes, diverse hydrazines, and malononitrile. At the end of the reaction, the separated catalyst can be reused after washing with warm EtOH and drying at 80°C. Fe_3_O_4_@SiO_2_@Tannic acid was used again for subsequent experiments under similar reaction conditions. The catalyst could be reused for the next cycle without any considerable loss of its activity. The yields of the product decreased only slightly after reusing the catalyst six times (Table 4). TEM images of the synthesized Fe_3_O_4_@SiO_2_@Tannic acid MNPs after one cycle of reaction and after six cycles of reaction are shown in Figure 5.

**TABLE 4:** Reusability of catalyst in the synthesis of **4a**.

Run	1	2	3	4	5	6	7
Yield	91	91	90	90	91	89	85
Mp (°C)	235–237	235–237	234–236	235–237	233–235	234–236	233–235

## Conclusion

In conclusion, Fe_3_O_4_@SiO_2_@Tannic acid was synthesized and investigated as a new, environmentally friendly, inexpensive, mild, and reusable catalyst for the mechanochemical synthesis of azo-linked 5-amino-pyrazole-4-carbonitriles. High yield, a simple work-up procedure, observance of green chemistry principles, eco-friendly procedure using natural ingredients, ease of separation, recyclability of the magnetic catalyst, and waste reduction are some advantages of this method.

## Data Availability

The original contributions presented in the study are included in the article/Supplementary Material. Further inquiries can be directed to the corresponding author.
