# Supplementary material for: Synthesis of Novel Azo-Linked 5-Amino-Pyrazole-4-Carbonitrile Derivatives Using Tannic Acid–Functionalized Silica-Coated Fe3O4 Nanoparticles as a Novel, Green, and Magnetically Separable Catalyst
Source: Front Chem. 2021 Nov 12;9:724745. doi: 10.3389/fchem.2021.724745 (PMC8633510; doi:10.3389/fchem.2021.724745)

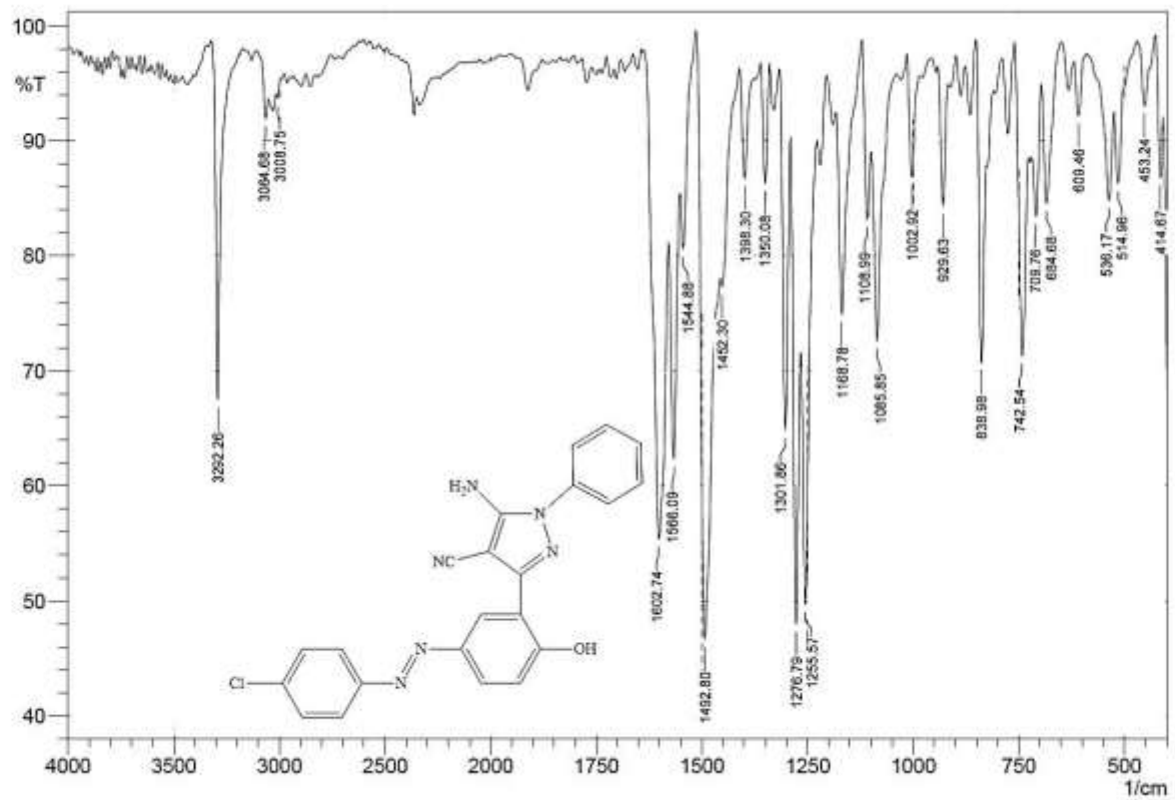

100
%T
90
80
70
60
50
40
4000
3500
3000
2500
2000
1750
1500
1250
1000
750
500
1/cm
3292.26
3064.68
3008.75
1602.74
1566.09
1544.88
1492.80
1452.30
1398.30
1350.08
1301.86
1276.79
1255.57
1168.78
1108.99
1085.85
1002.92
929.63
838.98
742.54
709.76
684.68
609.46
536.17
514.96
453.24
414.67
H2N
NC
N
N
OH
Cl
N
N

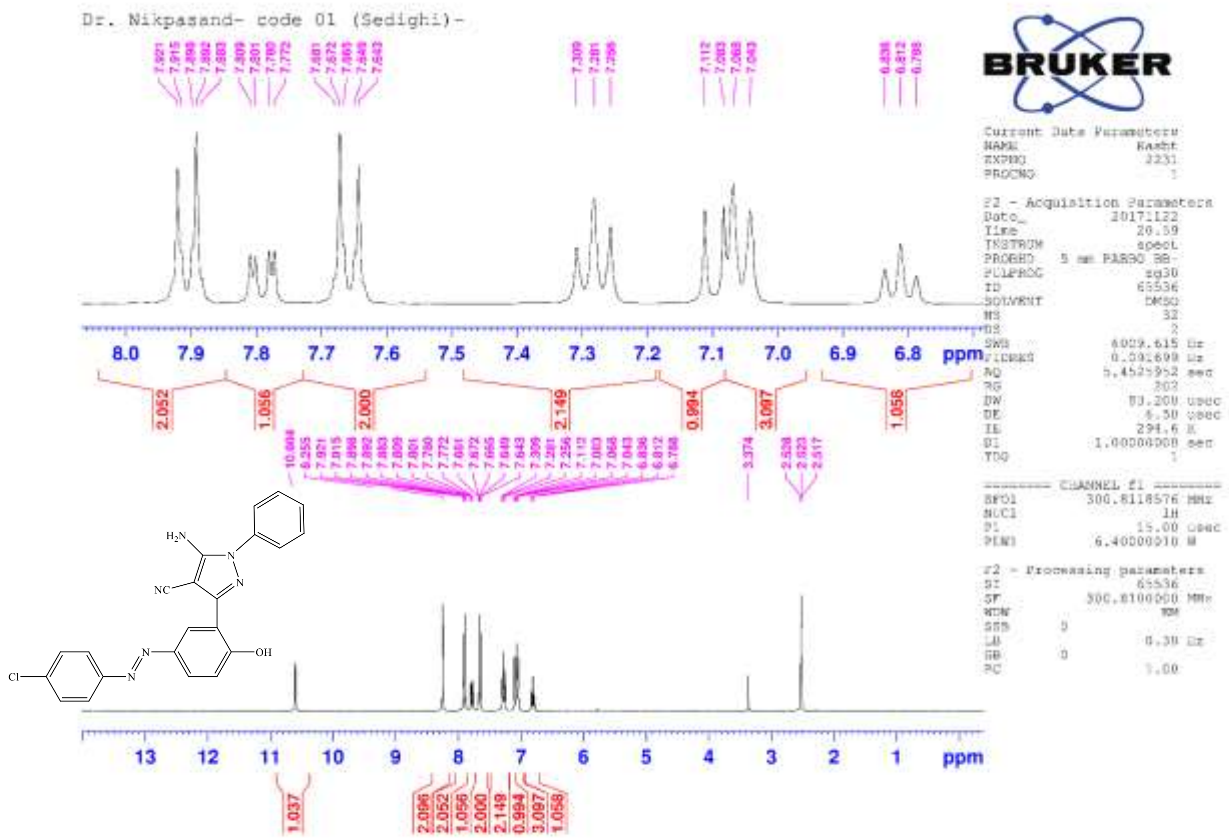
Dr. Nikpasand- code 01 (Sedighi)-
BRUKER
Current Data Parameters
NAME Kasht
EXPNO 2231
PROCNO 1
F2 - Acquisition Parameters
Date_ 20171122
Time 20.59
INSTRUM spect
PROBHD 5 mm PABBO BB-
PULPROG zg30
TD 65536
SOLVENT DMSO
NS 32
DS 2
SWH 6009.615 Hz
FIDRES 0.091699 Hz
AQ 5.4525952 sec
RG 203
DW 83.200 usec
DE 6.50 usec
TE 294.6 K
D1 1.00000000 sec
TD0 1
CHANNEL f1
SFO1 300.8118576 MHz
NUC1 1H
P1 15.00 usec
PLW1 6.40000010 W
F2 - Processing parameters
SI 65536
SF 300.8100000 MHz
WDW EM
SSB 0
LB 0.30 Hz
GB 0
PC 1.00
ppm

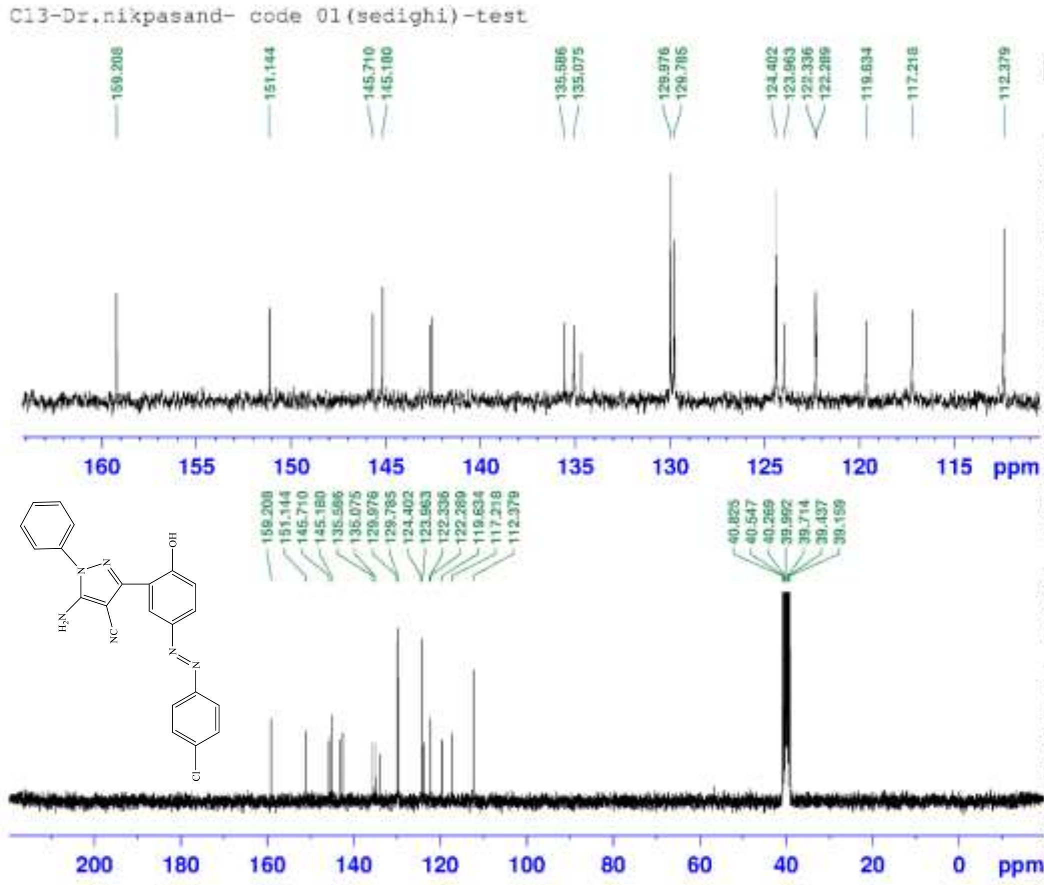

BRUKER

Current Data Parameters
NAME Rasgt
EXPNO 1321
PROCNO 1

F2 - Acquisition Parameters
Date_ 20171217
Time 9.43
INSTRUM spect
PROBHD 5 mm PABBO BB-
PULPROG zgpg30
TD 65536
SOLVENT DMSO
NS 800
DS 4
SWH 18115.941 Hz
FIDRES 0.276427 Hz
AQ 1.8087935 sec
RG 202
DW 27.600 usec
DE 6.50 usec
TE 296.0 K
D1 2.00000000 sec
D11 0.03000000 sec
TD0 1

======== CHANNEL f1 ========
SFO1 75.6462982 MHz
NUC1 13C
P1 10.00 usec
PLW1 30.00000000 W

======== CHANNEL f2 ========
SFO2 300.8112032 MHz
NUC2 1H
CPDPRG[2 waltz16
PCPD2 90.00 usec
PLW2 6.40000010 W
PLW12 0.17778000 W
PLW13 0.14399999 W

F2 - Processing parameters
SI 32768
SF 75.6387350 MHz
WDW EM
SSB 0
LB 1.00 Hz
GB 0
PC 1.40

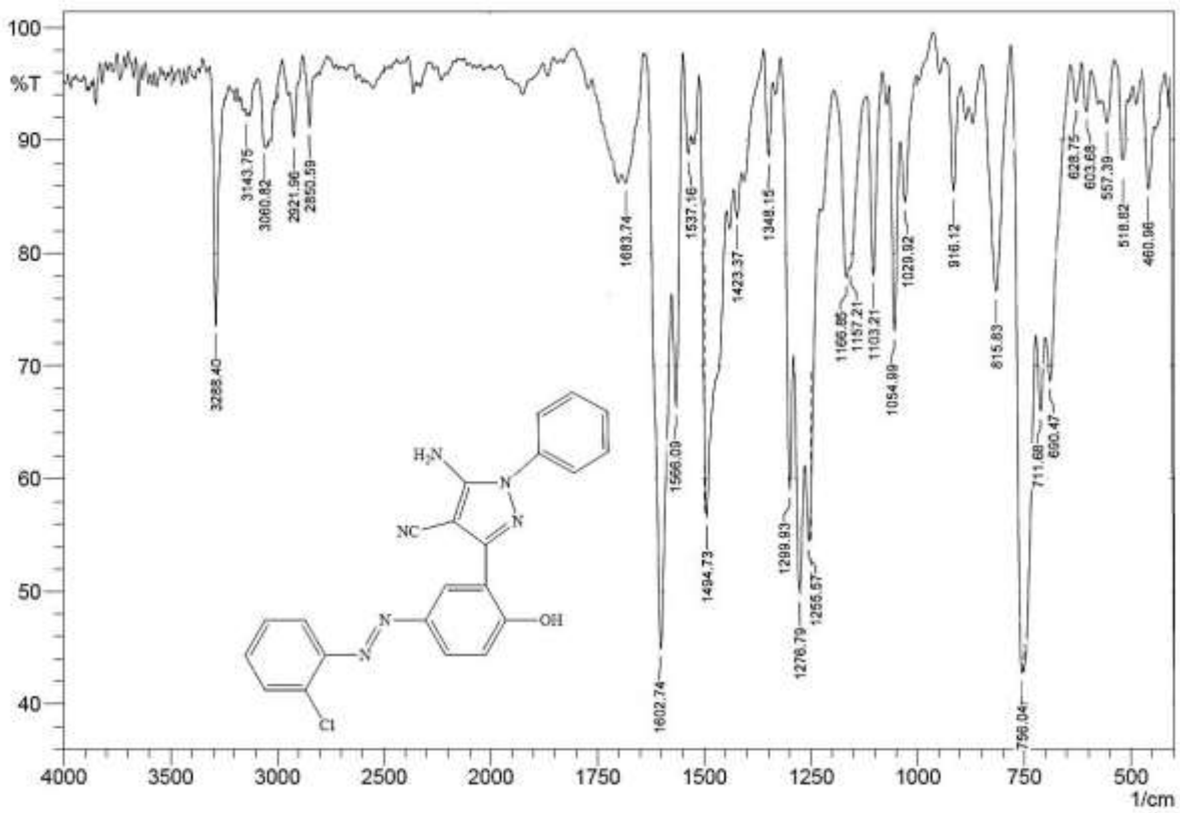
100
%T
90
80
70
60
50
40
4000
3500
3000
2500
2000
1750
1500
1250
1000
750
500
1/cm
3288.40
3143.75
3060.82
2921.96
2850.59
1683.74
1602.74
1566.09
1537.16
1494.73
1423.37
1348.15
1299.93
1276.79
1255.57
1166.85
1157.21
1103.21
1054.99
1029.92
916.12
815.83
756.04
711.68
690.47
628.75
603.68
557.39
518.82
460.96
H2N
N
N
NC
OH
N
N
Cl

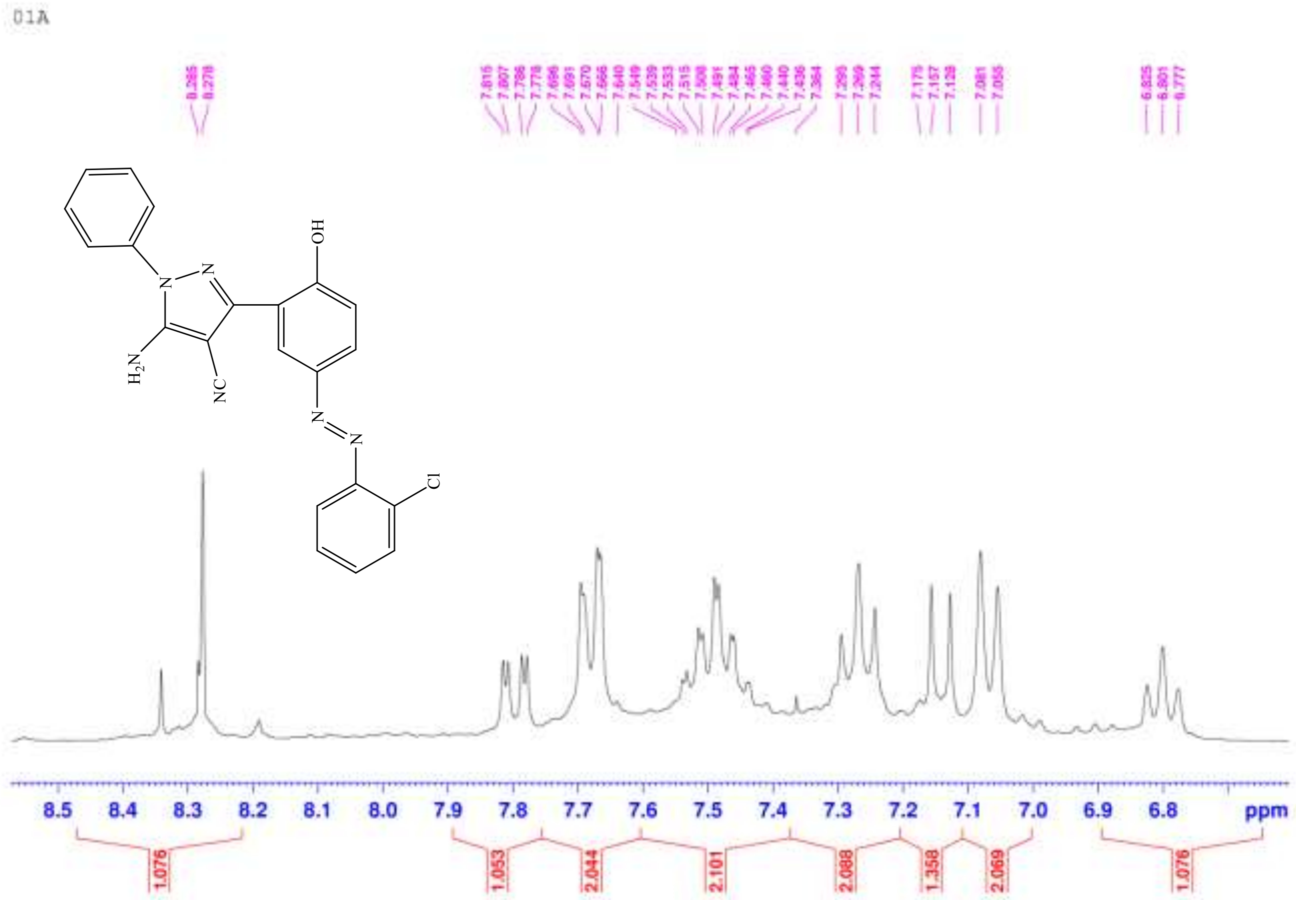

01A
8.285
8.278
7.815
7.807
7.786
7.778
7.696
7.691
7.670
7.666
7.640
7.549
7.539
7.533
7.515
7.508
7.491
7.484
7.465
7.460
7.440
7.436
7.364
7.295
7.269
7.244
7.175
7.157
7.128
7.081
7.055
6.825
6.801
6.777
OH
N
N
H2N
NC
N
N
Cl
8.5
8.4
8.3
8.2
8.1
8.0
7.9
7.8
7.7
7.6
7.5
7.4
7.3
7.2
7.1
7.0
6.9
6.8
ppm
1.076
1.053
2.044
2.101
2.088
1.358
2.069
1.076

01A

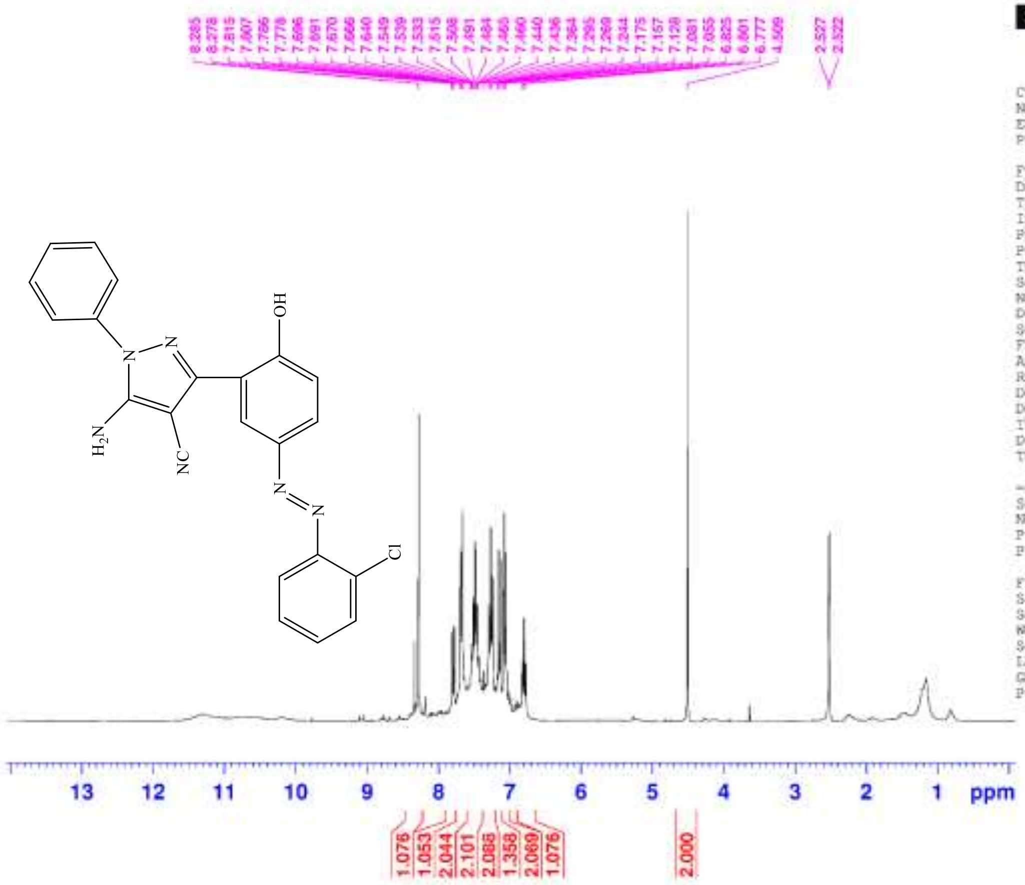

Current Data Parameters
NAME 12-29-2017
EXPNO 12
PROCNO 1

F2 - Acquisition Parameters
Date_ 20171230
Time 20.27
INSTRUM spect
PROBHD 5 mm PABBO BB-
PULPROG zg30
TD 65536
SOLVENT DMSO
NS 128
DS 2
SWH 6009.615 Hz
FIDRES 0.091699 Hz
AQ 5.4525952 sec
RG 33.22
DW 83.200 usec
DE 6.50 usec
TE 0 K
D1 1.00000000 sec
TD0 1

======== CHANNEL f1 ========
SFO1 300.8118576 MHz
NUC1 1H
P1 15.00 usec
PLW1 6.40000010 W

F2 - Processing parameters
SI 65536
SF 300.8100000 MHz
WDW EM
SSB 0
LB 0.30 Hz
GB 0
PC 1.00

C13-Dr.nikpasand- code 01A(sedighi)-test

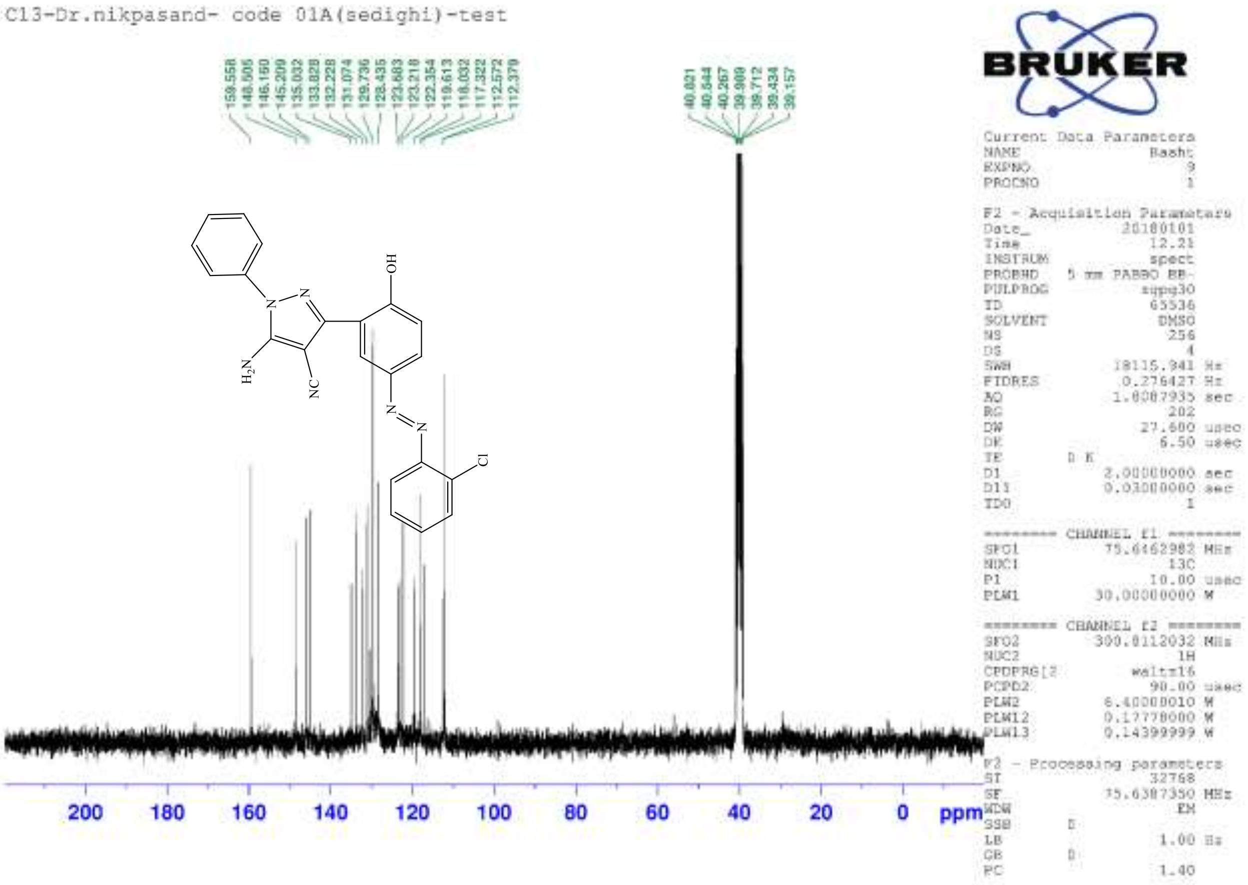

C13-Dr.nikpasand- code 01A(sedighi)-test

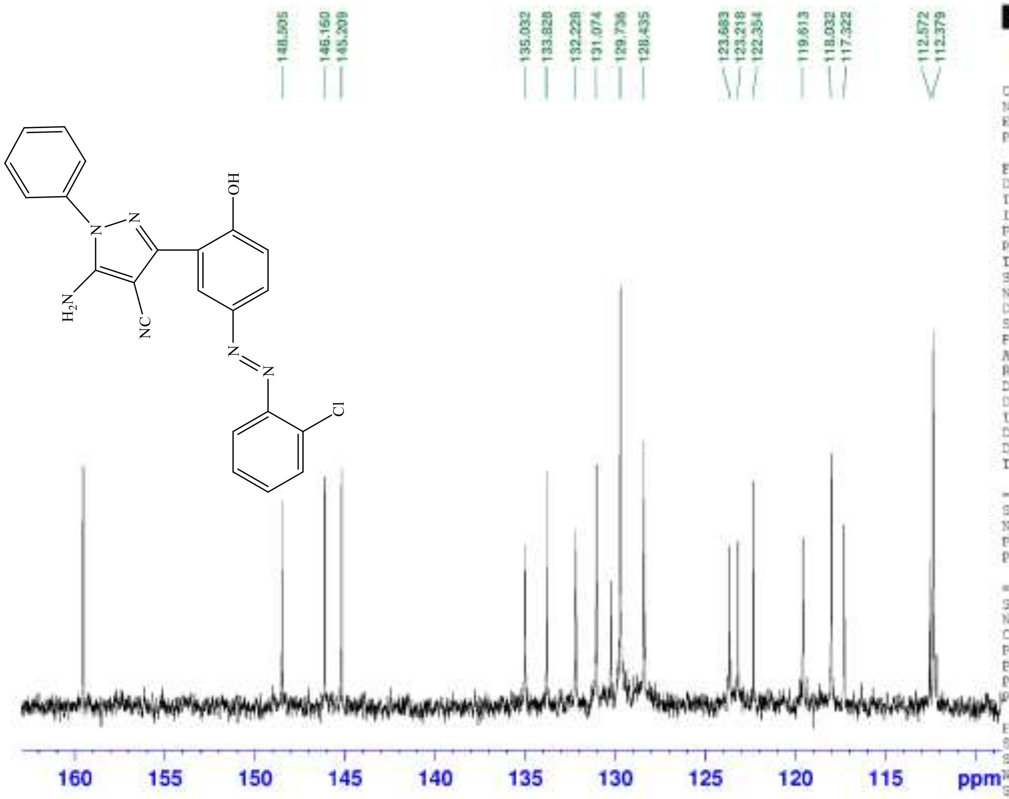


```
Current Data Parameters
NAME                Rasht
EXPNO                   9
PROCNO                  1

F2 - Acquisition Parameters
Date_            20180101
Time                12.21
INSTRUM             spect
PROBHD   5 mm PABBO BB-
PULPROG            zgpg30
TD                  65536
SOLVENT              DMSO
NS                    256
DS                      4
SWH             18115.941 Hz
FIDRES           0.276427 Hz
AQ              1.8087935 sec
RG                    202
DW                 27.600 usec
DE                   6.50 usec
TE                    0 K
D1             2.00000000 sec
D11            0.03000000 sec
TD0                     1

======== CHANNEL f1 ========
SFO1         75.6462982 MHz
NUC1                  13C
P1                  10.00 usec
PLW1          30.00000000 W

======== CHANNEL f2 ========
SFO2        300.8112032 MHz
NUC2                   1H
CPDPRG[2          waltz16
PCPD2               90.00 usec
PLW2           6.40000010 W
PLW12          0.17778000 W
PLW13          0.14399999 W

F2 - Processing parameters
SI                  32768
SF           75.6387350 MHz
WDW                    EM
SSB                     0
LB                   1.00 Hz
GB                      0
PC                   1.40
```

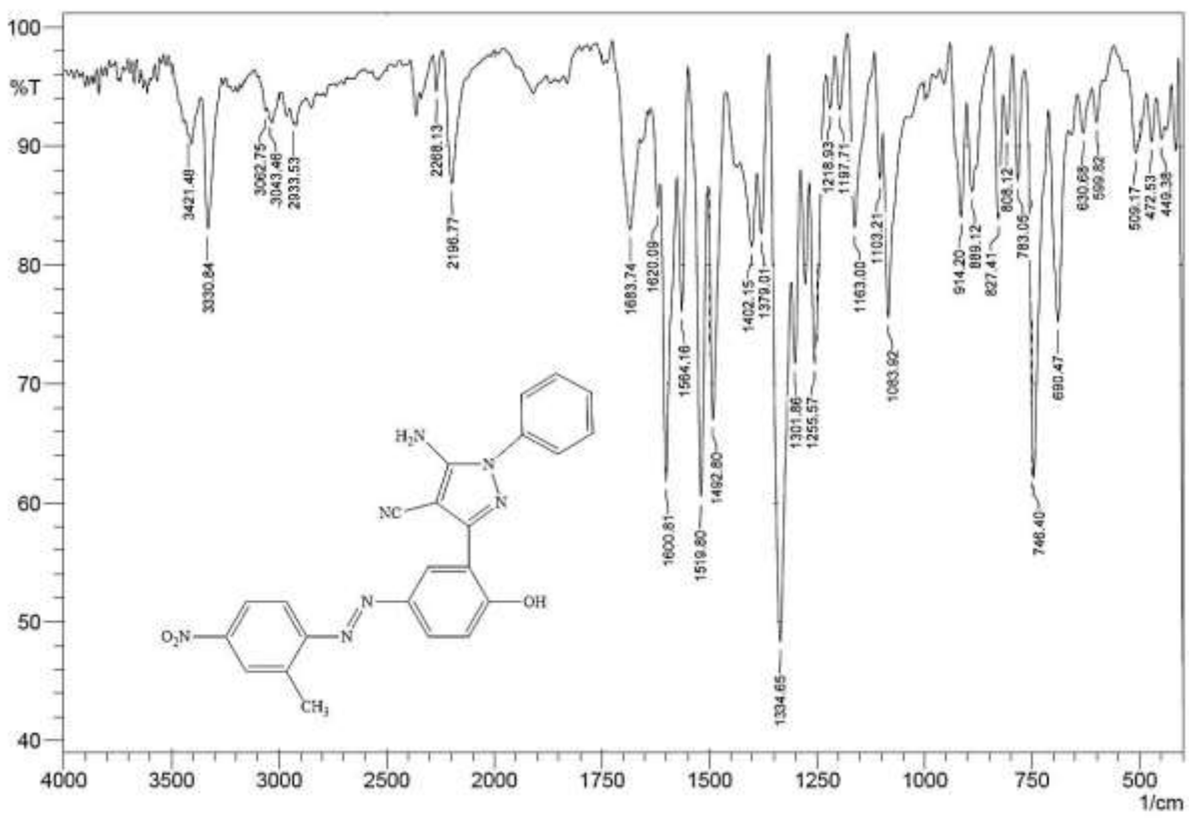
100
%T
90
80
70
60
50
40
4000
3500
3000
2500
2000
1750
1500
1250
1000
750
500
1/cm
3421.48
3330.84
3062.75
3043.46
2933.53
2268.13
2196.77
1683.74
1620.09
1600.81
1564.16
1519.80
1492.80
1402.15
1379.01
1334.65
1301.86
1255.57
1218.93
1197.71
1163.00
1103.21
1083.92
914.20
889.12
827.41
808.12
783.05
746.40
690.47
630.68
599.82
509.17
472.53
449.38
H₂N
N
N
NC
OH
N
N
O₂N
CH₃

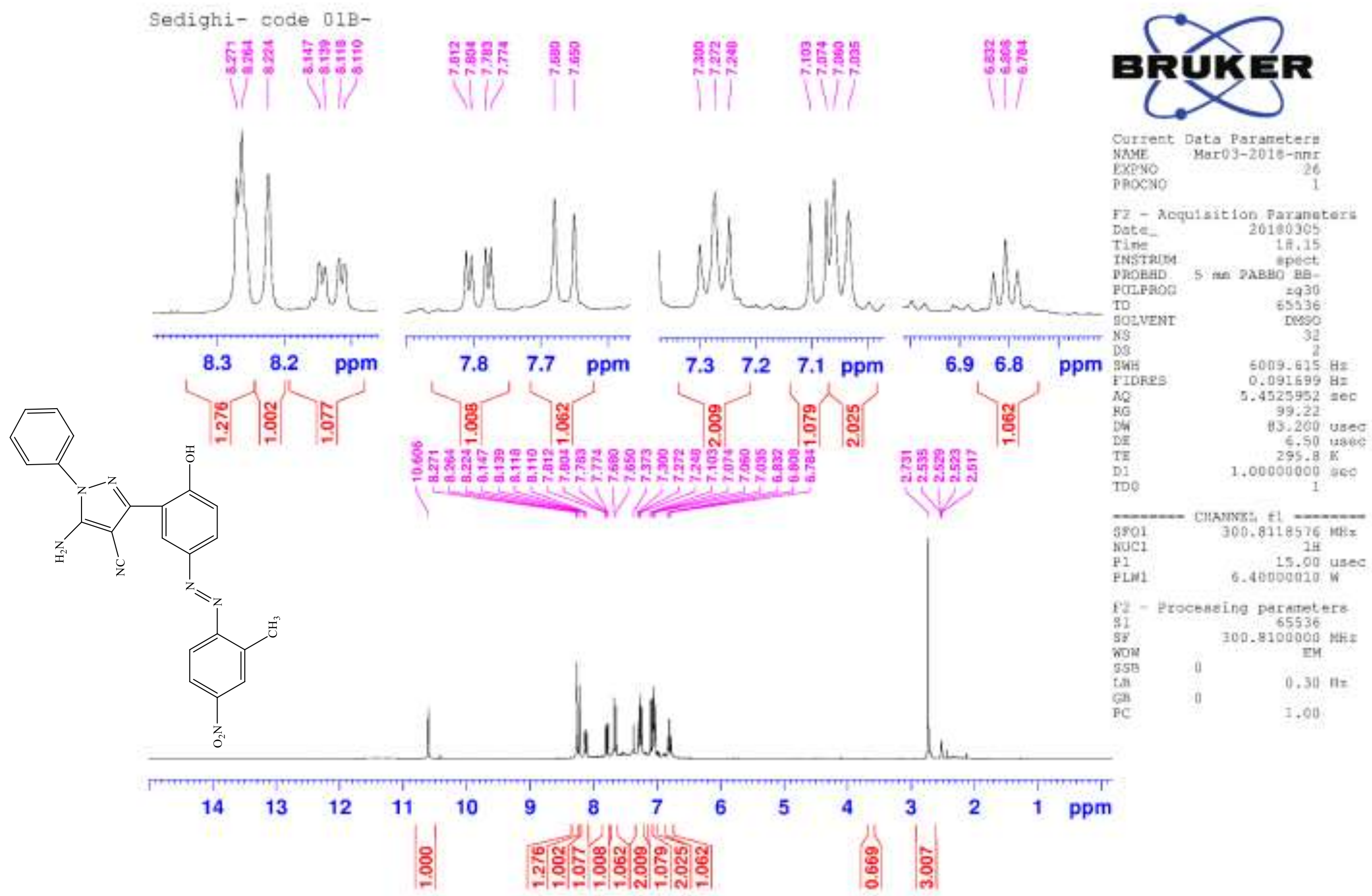
Sedighi- code 01B-
BRUKER
Current Data Parameters
NAME Mar03-2018-nmr
EXPNO 26
PROCNO 1
F2 - Acquisition Parameters
Date_ 20180305
Time 18.15
INSTRUM spect
PROBHD 5 mm PABBO BB-
PULPROG zg30
TD 65536
SOLVENT DMSO
NS 32
DS 2
SWH 6009.615 Hz
FIDRES 0.091699 Hz
AQ 5.4525952 sec
RG 99.22
DW 83.200 usec
DE 6.50 usec
TE 295.8 K
D1 1.00000000 sec
TD0 1
======== CHANNEL f1 ========
SFO1 300.8118576 MHz
NUC1 1H
P1 15.00 usec
PLW1 6.40000010 W
F2 - Processing parameters
SI 65536
SF 300.8100000 MHz
WDW EM
SSB 0
LB 0.30 Hz
GB 0
PC 1.00
8.271 8.264 8.224 8.147 8.139 8.118 8.110
7.812 7.804 7.783 7.774 7.680 7.650
7.300 7.272 7.248 7.103 7.074 7.060 7.035
6.832 6.808 6.784
10.606 7.373 2.731 2.535 2.529 2.523 2.517
1.276 1.002 1.077 1.008 1.062 2.009 1.079 2.025 1.062
1.000 0.669 3.007
ppm
H2N
NC
N
OH
CH3
O2N

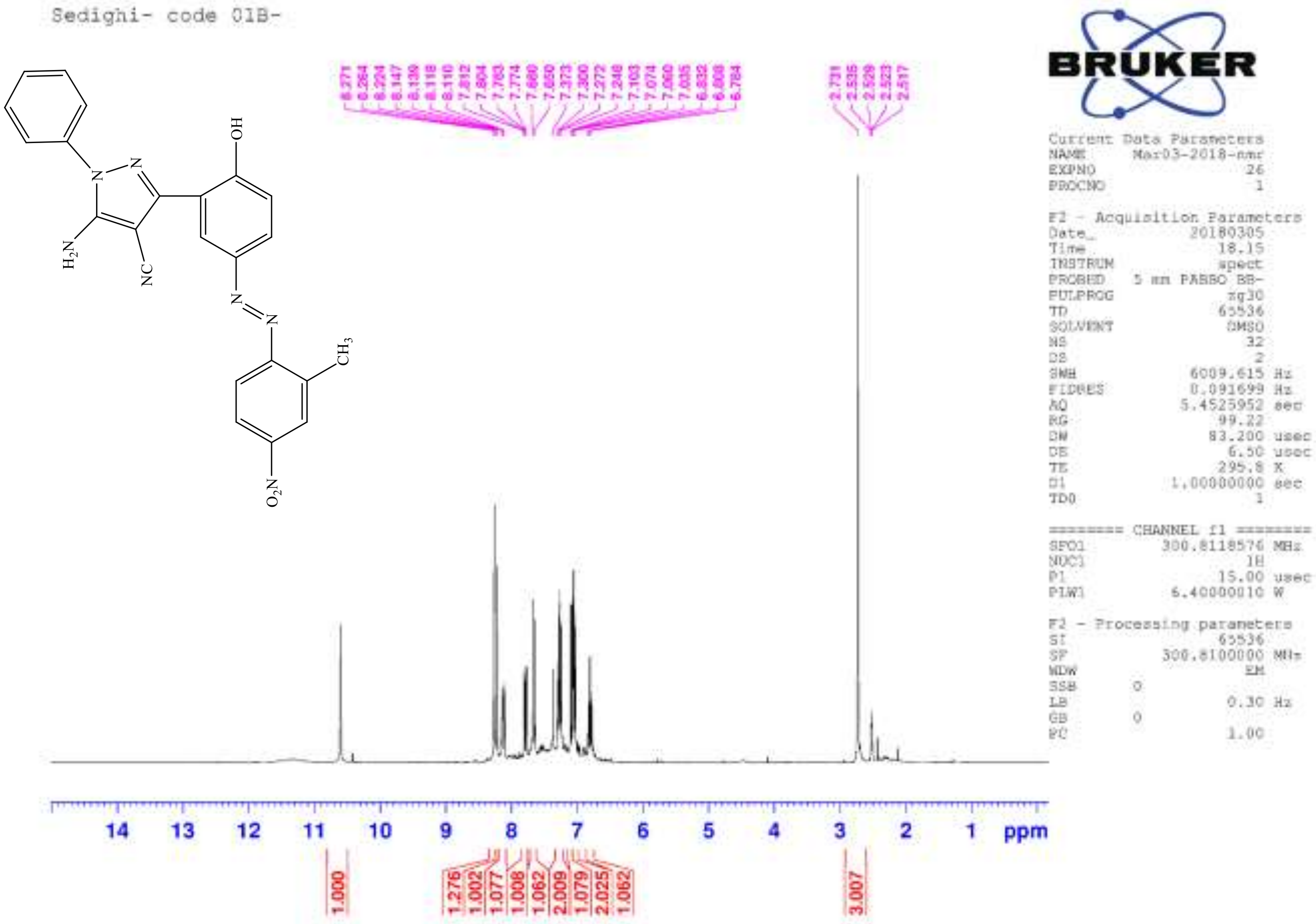

Sedighi- code 01B-
OH
N
N
H2N
NC
N
N
CH3
O2N
8.271 8.264 8.224 8.147 8.139 8.118 8.110 7.812 7.804 7.783 7.774 7.680 7.650 7.373 7.300 7.272 7.248 7.103 7.074 7.060 7.035 6.832 6.808 6.784
2.731 2.535 2.529 2.523 2.517
14 13 12 11 10 9 8 7 6 5 4 3 2 1 ppm
1.000
1.276 1.002 1.077 1.008 1.062 2.009 1.079 2.025 1.062
3.007
BRUKER
Current Data Parameters
NAME Mar03-2018-nmr
EXPNO 26
PROCNO 1
F2 - Acquisition Parameters
Date_ 20180305
Time 18.15
INSTRUM spect
PROBHD 5 mm PABBO BB-
PULPROG zg30
TD 65536
SOLVENT DMSO
NS 32
DS 2
SWH 6009.615 Hz
FIDRES 0.091699 Hz
AQ 5.4525952 sec
RG 99.22
DW 83.200 usec
DE 6.50 usec
TE 295.8 K
D1 1.00000000 sec
TD0 1
======== CHANNEL f1 ========
SFO1 300.8118576 MHz
NUC1 1H
P1 15.00 usec
PLW1 6.40000010 W
F2 - Processing parameters
SI 65536
SF 300.8100000 MHz
WDW EM
SSB 0
LB 0.30 Hz
GB 0
PC 1.00

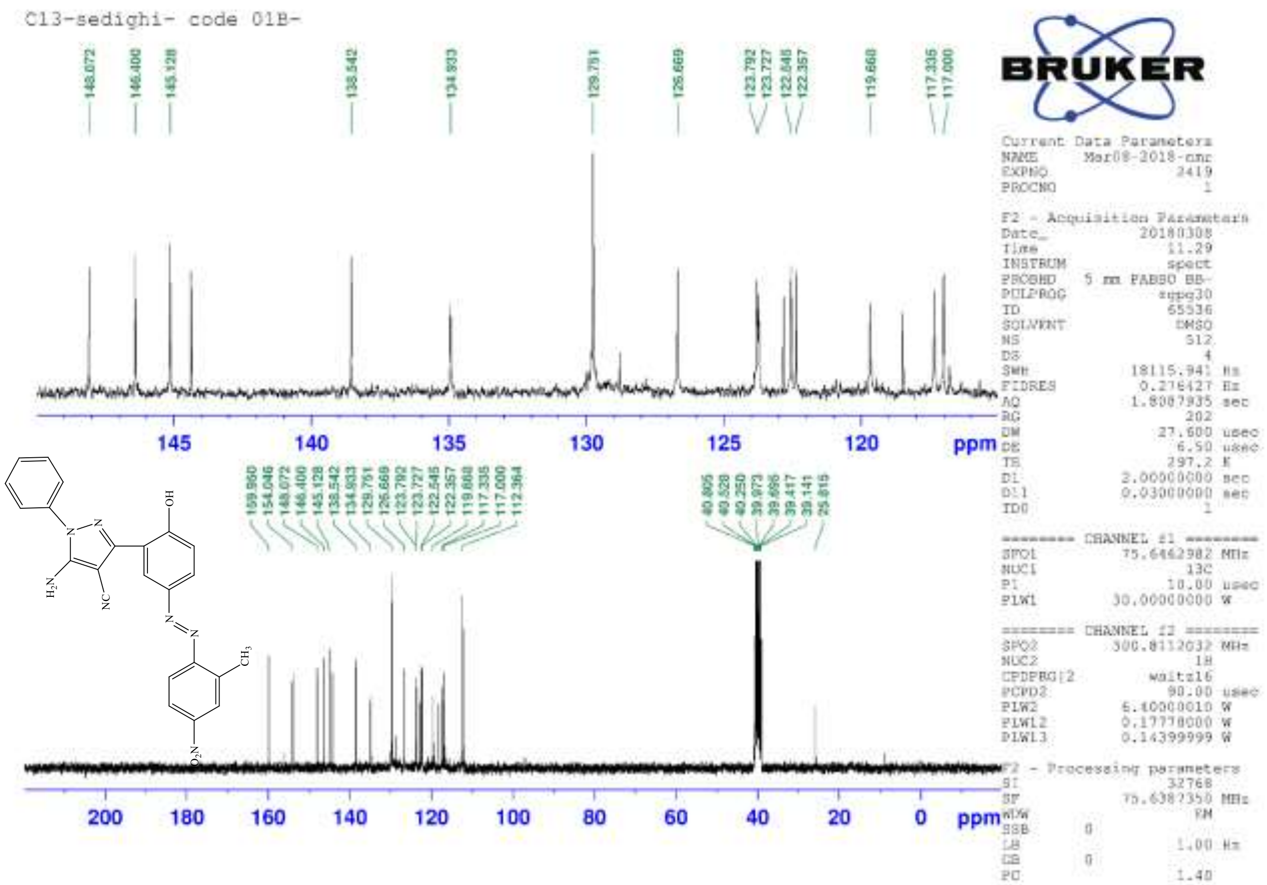
C13-sedighi- code 01B-
148.072
146.400
145.128
138.542
134.933
129.751
126.669
123.792
123.727
122.545
122.357
119.668
117.335
117.000
145
140
135
130
125
120
ppm
159.950
154.046
148.072
146.400
145.128
138.542
134.933
129.751
126.669
123.792
123.727
122.545
122.357
119.668
117.335
117.000
112.364
40.805
40.528
40.250
39.973
39.695
39.417
39.141
25.815
200
180
160
140
120
100
80
60
40
20
0
ppm
OH
N
N
NC
H_2N
N
N
CH_3
O_2N
BRUKER
Current Data Parameters
NAME Mar08-2018-cnc
EXPNO 2419
PROCNO 1
F2 - Acquisition Parameters
Date_ 20180308
Time 11.29
INSTRUM spect
PROBHD 5 mm PABBO BB-
PULPROG zgpg30
TD 65536
SOLVENT DMSO
NS 512
DS 4
SWH 18115.941 Hz
FIDRES 0.276427 Hz
AQ 1.8087935 sec
RG 202
DW 27.600 usec
DE 6.50 usec
TE 297.2 K
D1 2.00000000 sec
D11 0.03000000 sec
TD0 1
======== CHANNEL f1 ========
SFO1 75.6462982 MHz
NUC1 13C
P1 10.00 usec
PLW1 30.00000000 W
======== CHANNEL f2 ========
SFO2 300.8112032 MHz
NUC2 1H
CPDPRG[2 waltz16
PCPD2 90.00 usec
PLW2 6.40000010 W
PLW12 0.17778000 W
PLW13 0.14399999 W
F2 - Processing parameters
SI 32768
SF 75.6387350 MHz
WDW EM
SSB 0
LB 1.00 Hz
GB 0
PC 1.40

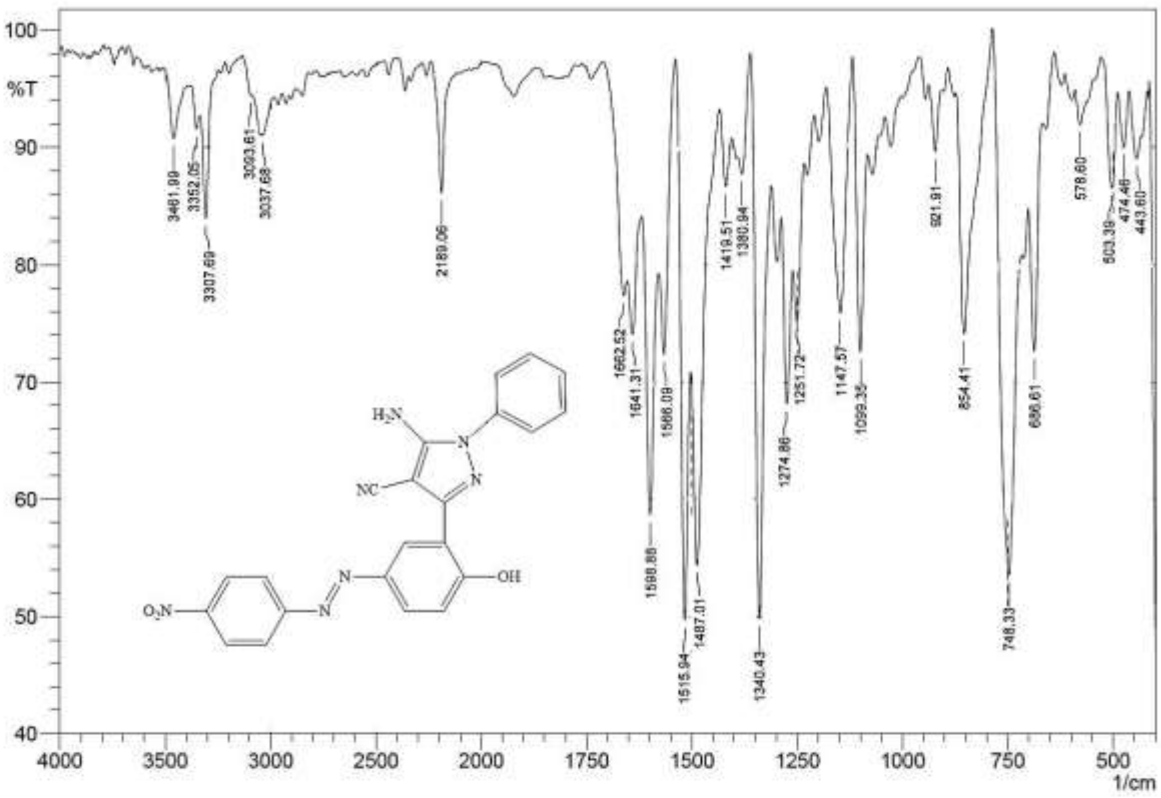

%T
100
90
80
70
60
50
40
4000
3500
3000
2500
2000
1750
1500
1250
1000
750
500
1/cm
3461.99
3352.05
3307.69
3093.61
3037.68
2189.06
1662.52
1641.31
1598.88
1566.09
1515.94
1487.01
1419.51
1380.94
1340.43
1274.86
1251.72
1147.57
1099.35
921.91
854.41
748.33
686.61
578.60
503.39
474.46
443.60
H2N
N
N
NC
N
N
OH
O2N

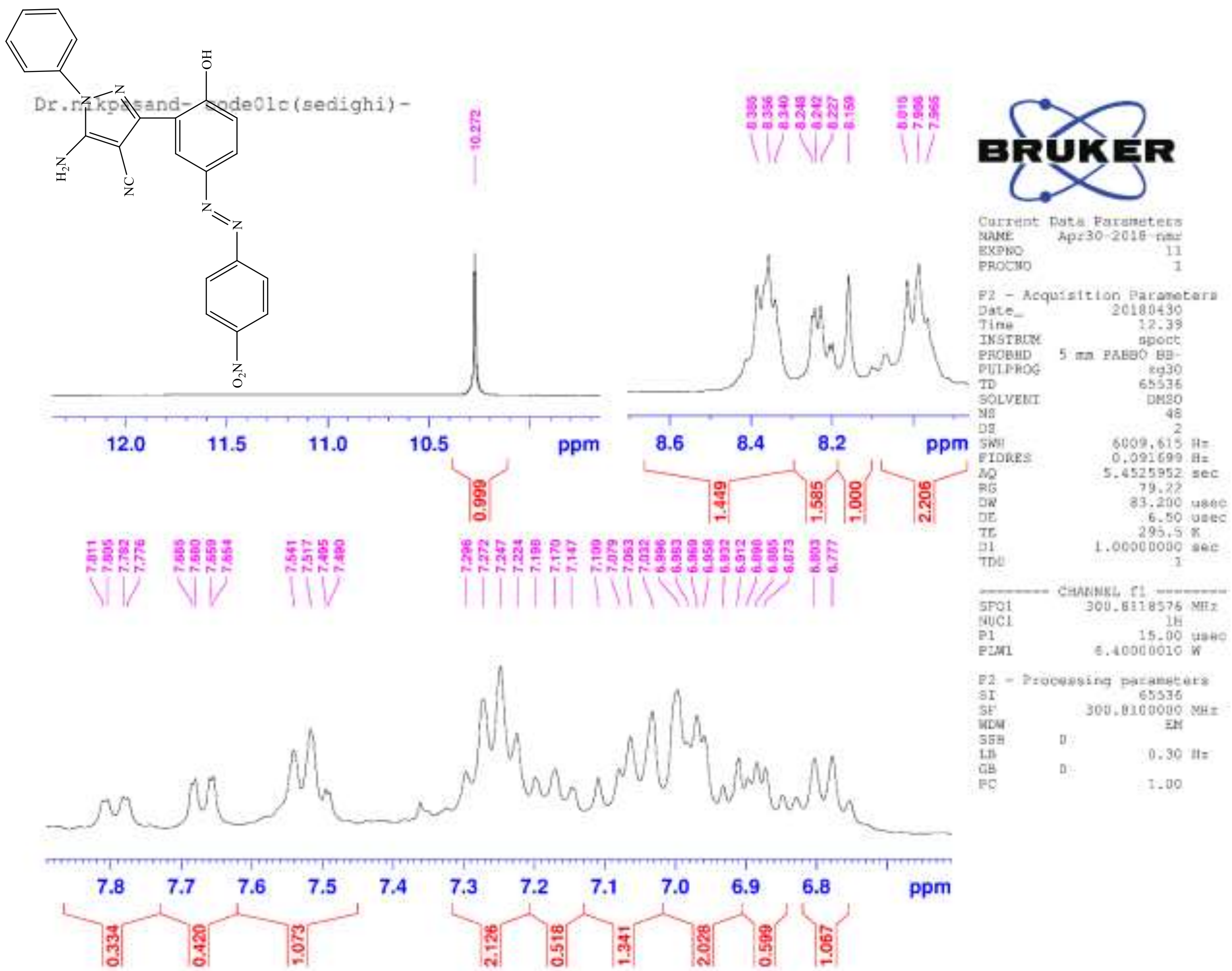
Dr.nikpasand-code01c(sedighi)-
OH
N
N
H_2N
NC
N
N
O_2N
10.272
8.385 8.356 8.340 8.248 8.242 8.227 8.159 8.015 7.986 7.965
12.0 11.5 11.0 10.5 ppm
8.6 8.4 8.2 ppm
0.999
1.449 1.585 1.000 2.206
7.811 7.805 7.782 7.776 7.685 7.680 7.659 7.654 7.541 7.517 7.495 7.490 7.296 7.272 7.247 7.224 7.198 7.170 7.147 7.109 7.079 7.063 7.032 6.996 6.983 6.969 6.958 6.932 6.912 6.898 6.885 6.873 6.803 6.777
7.8 7.7 7.6 7.5 7.4 7.3 7.2 7.1 7.0 6.9 6.8 ppm
0.334 0.420 1.073 2.126 0.518 1.341 2.028 0.599 1.067
BRUKER
Current Data Parameters
NAME Apr30-2018-nmr
EXPNO 11
PROCNO 1
F2 - Acquisition Parameters
Date_ 20180430
Time 12.39
INSTRUM spect
PROBHD 5 mm PABBO BB-
PULPROG zg30
TD 65536
SOLVENT DMSO
NS 48
DS 2
SWH 6009.615 Hz
FIDRES 0.091699 Hz
AQ 5.4525952 sec
RG 79.22
DW 83.200 usec
DE 6.50 usec
TE 295.5 K
D1 1.00000000 sec
TD0 1
======== CHANNEL f1 ========
SFO1 300.8118576 MHz
NUC1 1H
P1 15.00 usec
PLW1 6.40000010 W
F2 - Processing parameters
SI 65536
SF 300.8100000 MHz
WDW EM
SSB 0
LB 0.30 Hz
GB 0
PC 1.00

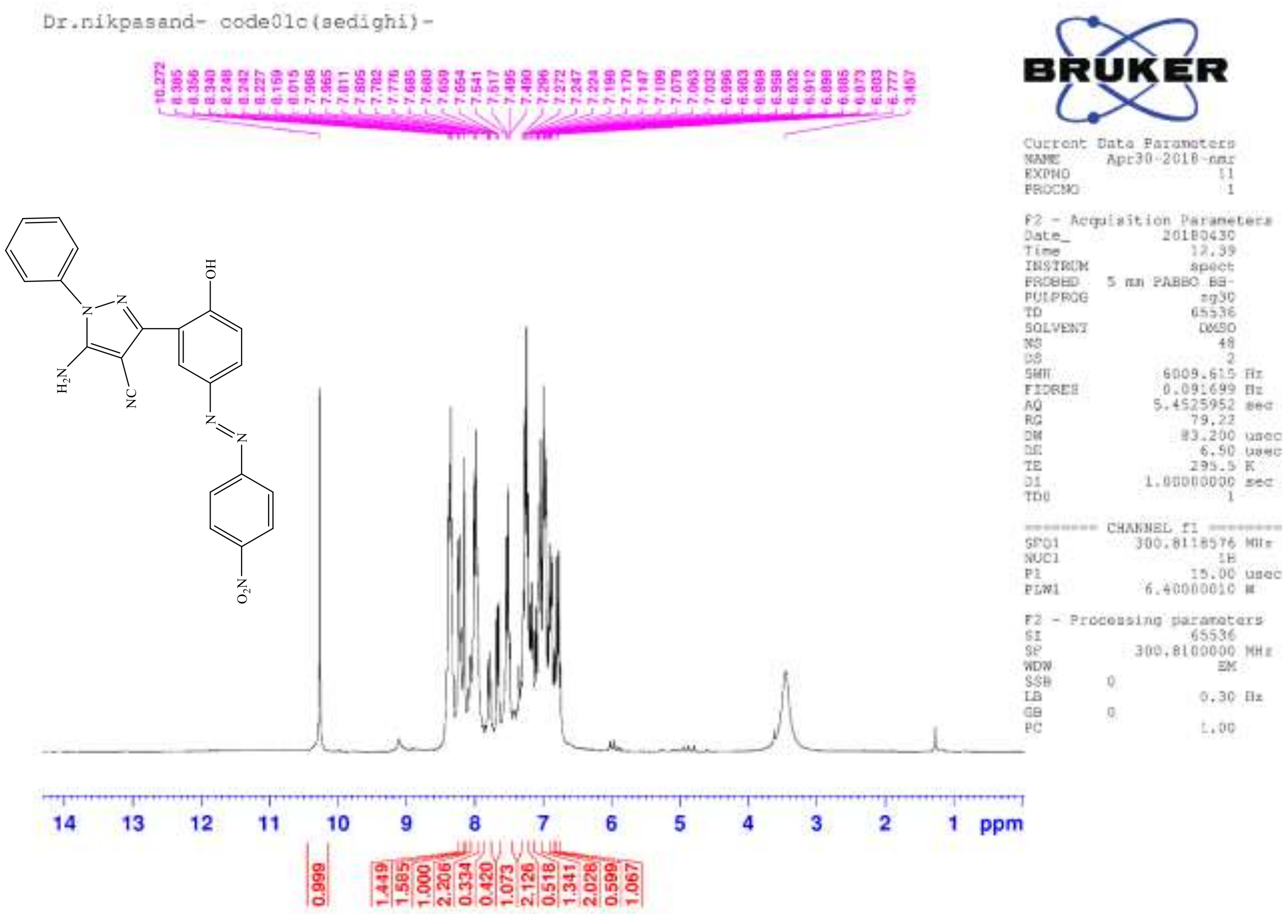

Dr.nikpasand- code01c(sedighi)-
10.272 8.385 8.356 8.340 8.248 8.242 8.227 8.159 8.015 7.986 7.965 7.811 7.805 7.782 7.776 7.685 7.680 7.659 7.654 7.541 7.517 7.495 7.490 7.296 7.272 7.247 7.224 7.198 7.170 7.147 7.109 7.079 7.063 7.032 6.996 6.983 6.969 6.958 6.932 6.912 6.898 6.885 6.873 6.803 6.777 3.457
H2N
NC
N
N
OH
N
N
O2N
14 13 12 11 10 9 8 7 6 5 4 3 2 1 ppm
0.999
1.449 1.585 1.000 2.206 0.334 0.420 1.073 2.126 0.518 1.341 2.028 0.599 1.067
BRUKER
Current Data Parameters
NAME Apr30-2018-nmr
EXPNO 11
PROCNO 1
F2 - Acquisition Parameters
Date_ 20180430
Time 12.39
INSTRUM spect
PROBHD 5 mm PABBO BB-
PULPROG zg30
TD 65536
SOLVENT DMSO
NS 48
DS 2
SWH 6009.615 Hz
FIDRES 0.091699 Hz
AQ 5.4525952 sec
RG 79.22
DW 83.200 usec
DE 6.50 usec
TE 295.5 K
D1 1.00000000 sec
TD0 1
======== CHANNEL f1 ========
SFO1 300.8118576 MHz
NUC1 1H
P1 15.00 usec
PLW1 6.40000010 W
F2 - Processing parameters
SI 65536
SF 300.8100000 MHz
WDW EM
SSB 0
LB 0.30 Hz
GB 0
PC 1.00

C13-Dr.Nikpasand- code 01C(sedighi)-

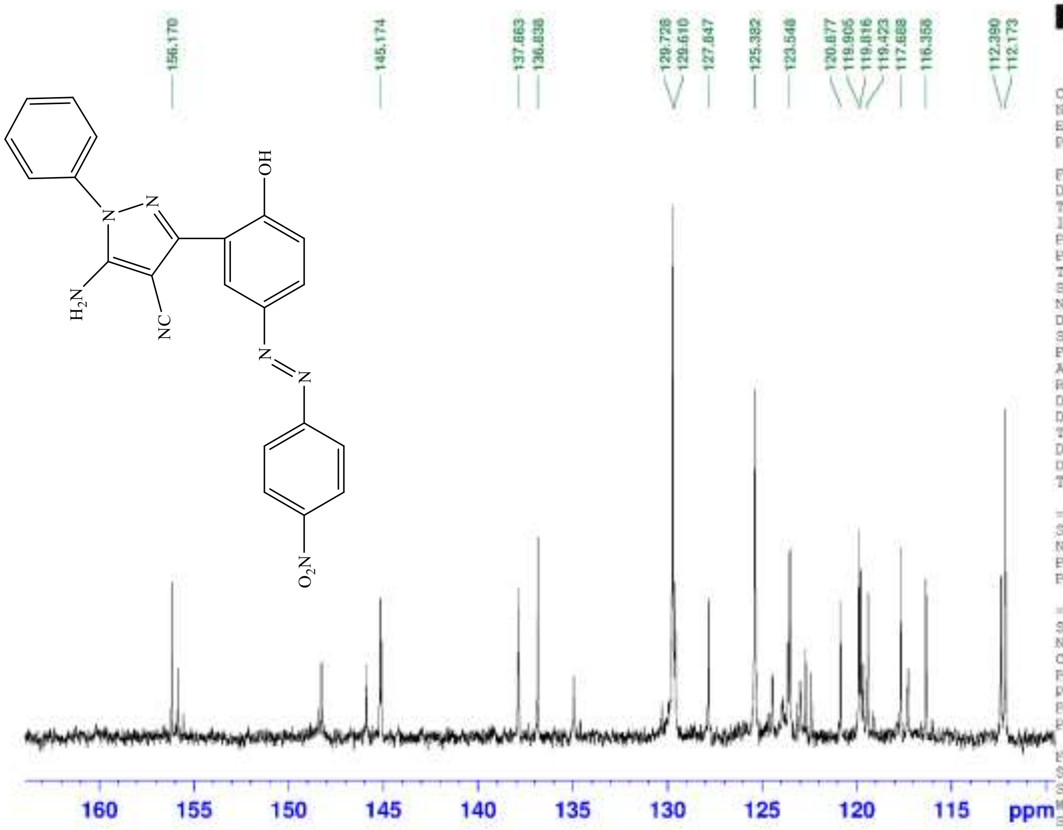


```
Current Data Parameters
NAME       May06-2018-nmr
EXPNO                   4
PROCNO                  1

F2 - Acquisition Parameters
Date_            20180506
Time                11.16
INSTRUM             spect
PROBHD   5 mm PABBO BB-
PULPROG            zgpg30
TD                  65536
SOLVENT              DMSO
NS                    400
DS                      4
SWH            18115.941 Hz
FIDRES          0.276427 Hz
AQ             1.8087935 sec
RG                    202
DW                 27.600 usec
DE                   6.50 usec
TE                  296.8 K
D1            2.00000000 sec
D11           0.03000000 sec
TD0                     1

======== CHANNEL f1 ========
SFO1          75.6462982 MHz
NUC1                  13C
P1                  10.00 usec
PLW1         30.00000000 W

======== CHANNEL f2 ========
SFO2         300.8112032 MHz
NUC2                   1H
CPDPRG[2          waltz16
PCPD2               90.00 usec
PLW2          6.40000010 W
PLW12         0.17778000 W
PLW13         0.14399999 W

F2 - Processing parameters
SI                  32768
SF            75.6387350 MHz
WDW                    EM
SSB        0
LB                   1.00 Hz
GB         0
PC                   1.40
```

C13-Dr.Nikpasand- code 01C(sedighi)-

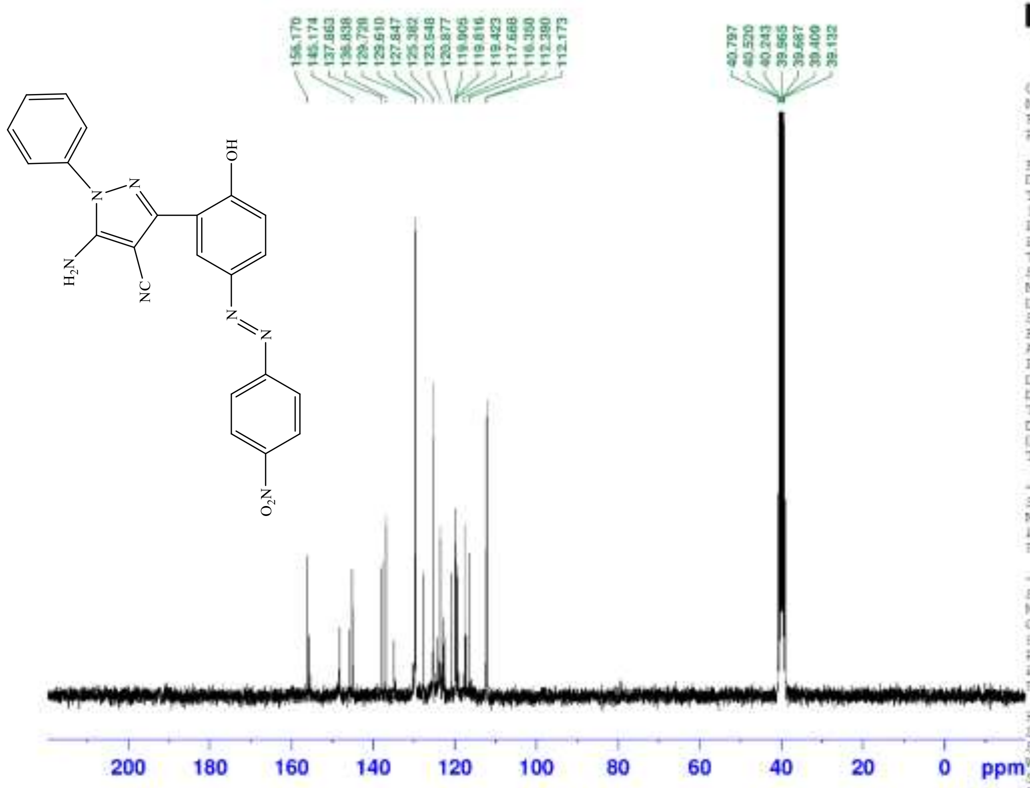

BRUKER

```
Current Data Parameters
NAME        May06-2018-nmr
EXPNO                4
PROCNO               1

F2 - Acquisition Parameters
Date_          20180506
Time              11.16
INSTRUM           spect
PROBHD   5 mm PABBO BB-
PULPROG          zgpg30
TD                65536
SOLVENT            DMSO
NS                  400
DS                    4
SWH           18115.941 Hz
FIDRES         0.276427 Hz
AQ            1.8087935 sec
RG                  202
DW               27.600 usec
DE                 6.50 usec
TE                296.8 K
D1           2.00000000 sec
D11          0.03000000 sec
TD0                   1

======== CHANNEL f1 ========
SFO1         75.6462982 MHz
NUC1                13C
P1                10.00 usec
PLW1        30.00000000 W

======== CHANNEL f2 ========
SFO2        300.8112032 MHz
NUC2                 1H
CPDPRG[2           waltz16
PCPD2             90.00 usec
PLW2         6.40000010 W
PLW12        0.17778000 W
PLW13        0.14399999 W

F2 - Processing parameters
SI                32768
SF           75.6387350 MHz
WDW                  EM
SSB      0
LB                 1.00 Hz
GB       0
PC                 1.40
```

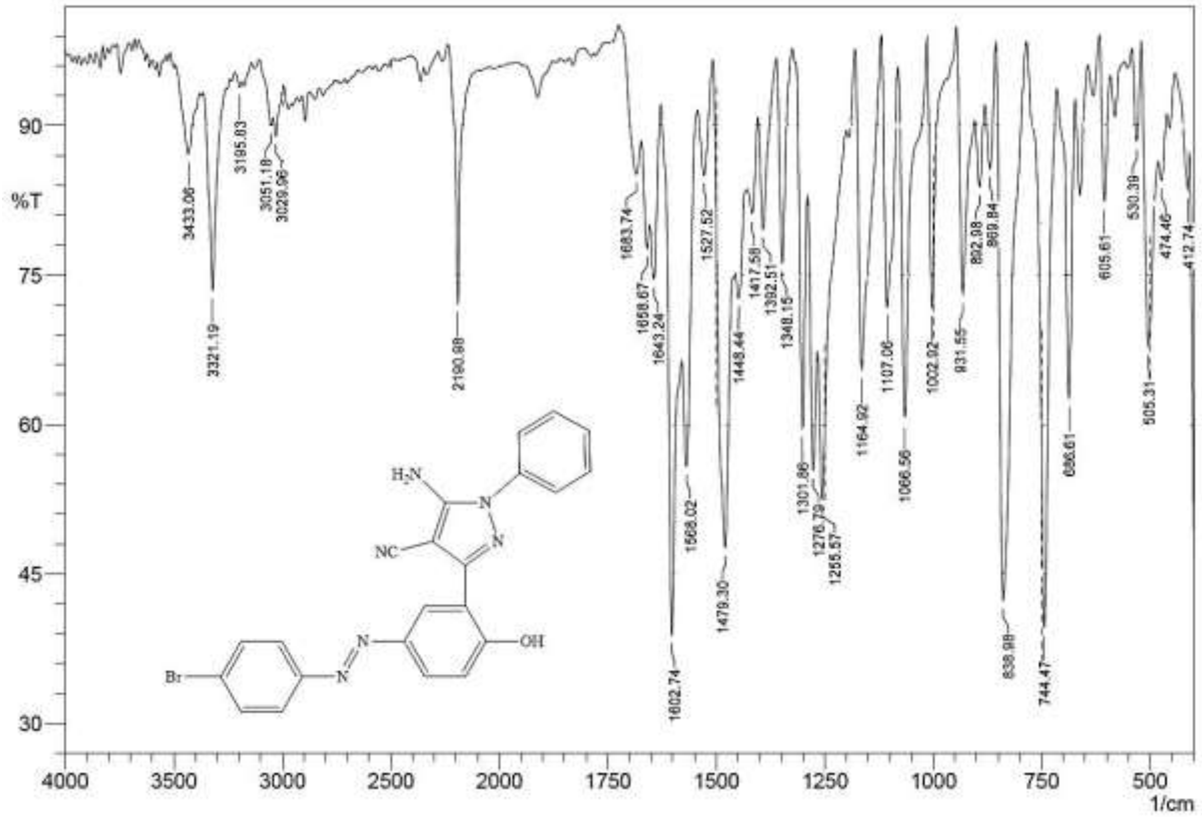

%T
90
75
60
45
30
4000
3500
3000
2500
2000
1750
1500
1250
1000
750
500
1/cm
3433.06
3321.19
3195.83
3051.18
3029.06
2190.88
1683.74
1658.67
1643.24
1602.74
1568.02
1527.52
1479.30
1448.44
1417.58
1392.51
1348.15
1301.86
1276.79
1255.57
1164.92
1107.06
1066.56
1002.92
931.55
892.98
869.84
838.98
744.47
686.61
605.61
530.39
505.31
474.46
412.74
H2N
NC
N
OH
Br

Dr.nikpasand- code 01H(sedighi)-

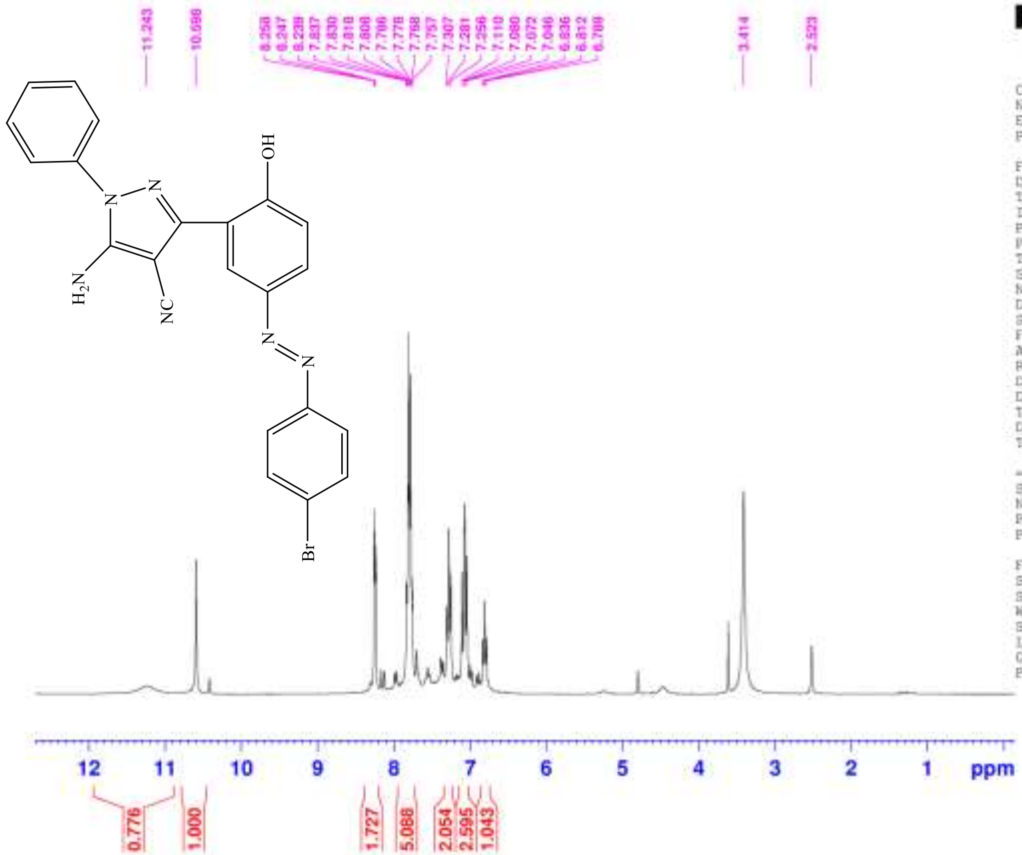


```
Current Data Parameters
NAME      May22-2018-nmr
EXPNO                 32
PROCNO                 1

F2 - Acquisition Parameters
Date_           20180523
Time               12.39
INSTRUM            spect
PROBHD   5 mm PABBO BB-
PULPROG             zg30
TD                 65536
SOLVENT             DMSO
NS                   168
DS                     2
SWH             6009.615 Hz
FIDRES          0.091699 Hz
AQ             5.4525952 sec
RG                113.32
DW                83.200 usec
DE                  6.50 usec
TE                 297.3 K
D1            1.00000000 sec
TD0                    1

======== CHANNEL f1 ========
SFO1         300.8118576 MHz
NUC1                  1H
P1                 15.00 usec
PLW1          6.40000010 W

F2 - Processing parameters
SI                 65536
SF           300.8100000 MHz
WDW                   EM
SSB        0
LB                  0.30 Hz
GB         0
PC                  1.00
```

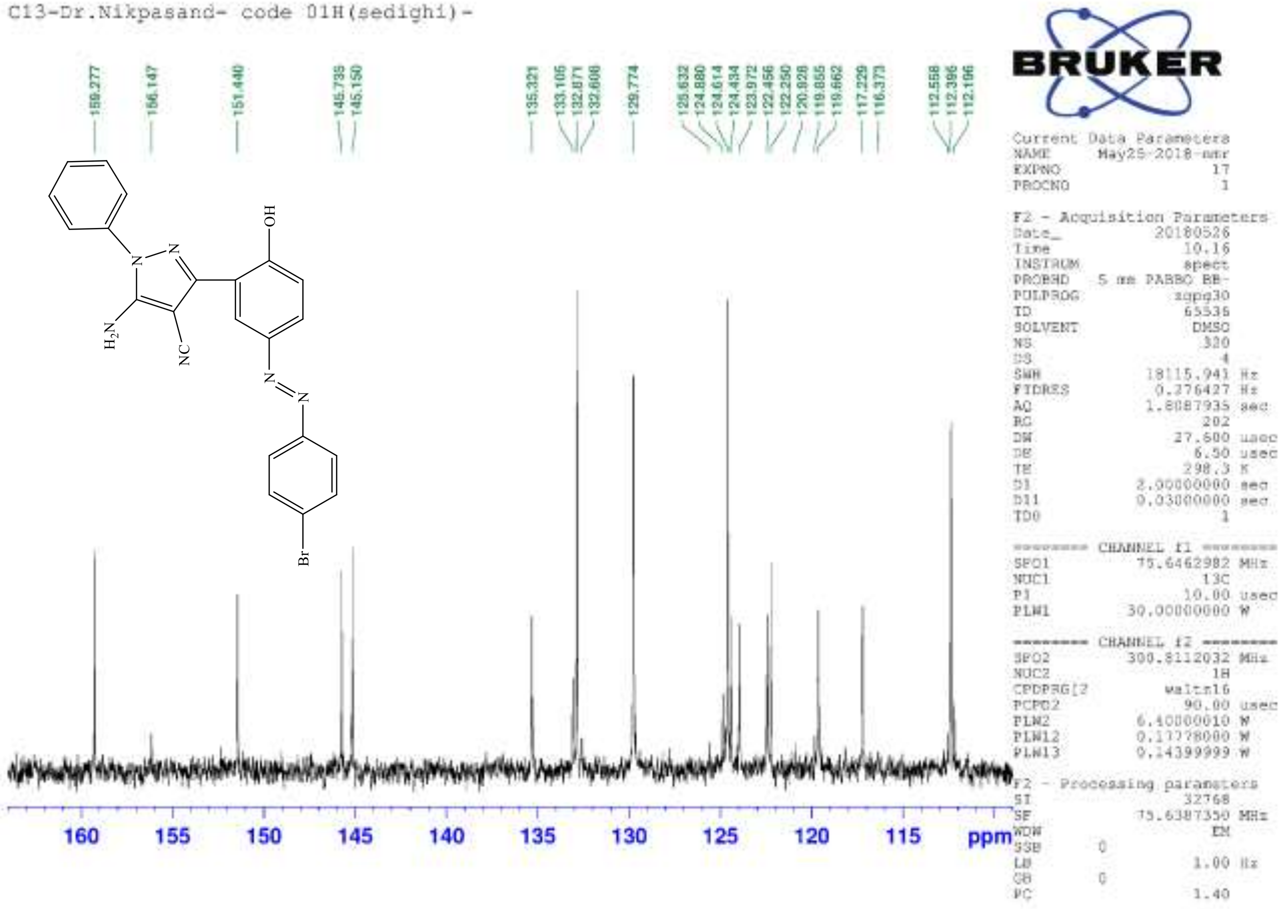
C13-Dr.Nikpasand- code 01H(sedighi)-
159.277
156.147
151.440
145.735
145.150
135.321
133.105
132.871
132.606
129.774
125.632
124.880
124.614
124.434
123.972
122.456
122.250
120.928
119.855
119.662
117.229
116.373
112.558
112.396
112.196
H2N
NC
N
N
OH
N
N
Br
160
155
150
145
140
135
130
125
120
115
ppm
BRUKER
Current Data Parameters
NAME May25-2018-nmr
EXPNO 17
PROCNO 1
F2 - Acquisition Parameters
Date_ 20180526
Time 10.16
INSTRUM spect
PROBHD 5 mm PABBO BB-
PULPROG zgpg30
TD 65536
SOLVENT DMSO
NS 320
DS 4
SWH 18115.941 Hz
FIDRES 0.276427 Hz
AQ 1.8087935 sec
RG 202
DW 27.600 usec
DE 6.50 usec
TE 298.3 K
D1 2.00000000 sec
D11 0.03000000 sec
TD0 1
======== CHANNEL f1 ========
SFO1 75.6462982 MHz
NUC1 13C
P1 10.00 usec
PLW1 30.00000000 W
======== CHANNEL f2 ========
SFO2 300.8112032 MHz
NUC2 1H
CPDPRG[2 waltz16
PCPD2 90.00 usec
PLW2 6.40000010 W
PLW12 0.13778000 W
PLW13 0.14399999 W
F2 - Processing parameters
SI 32768
SF 75.6387350 MHz
WDW EM
SSB 0
LB 1.00 Hz
GB 0
PC 1.40

C13-Dr.Nikpasand- code 01H(sedighi)-

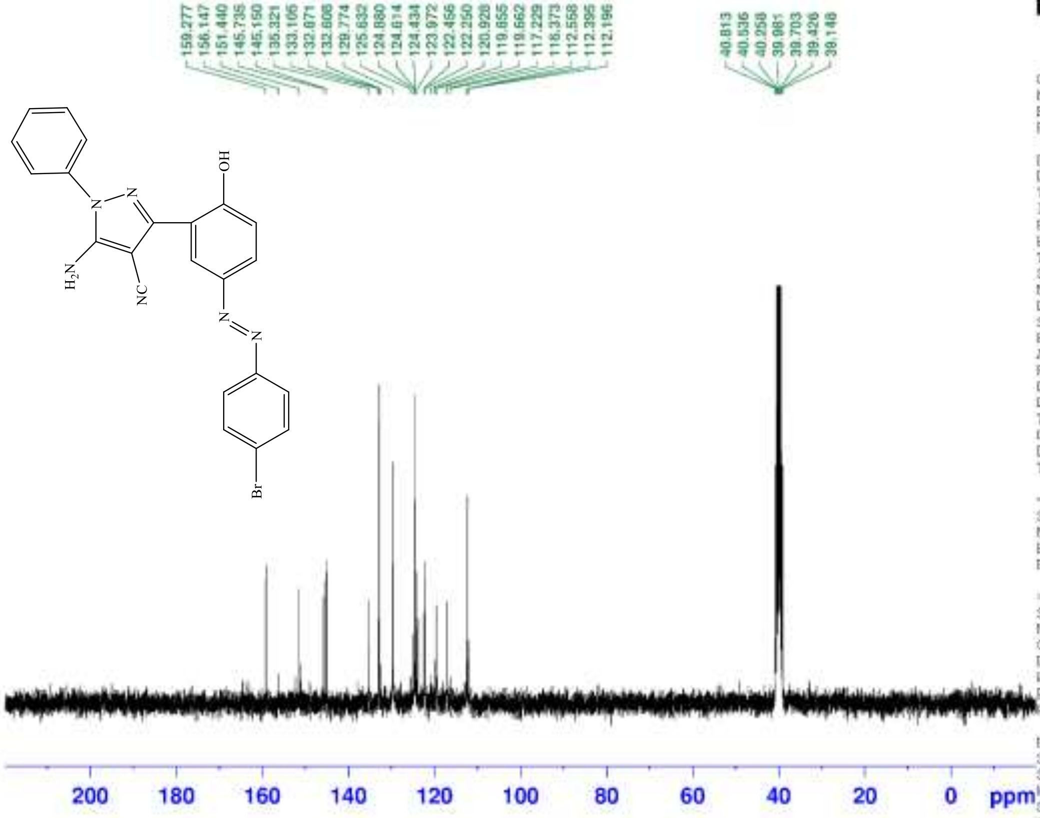

Current Data Parameters
NAME May25-2018-nmr
EXPNO 17
PROCNO 1

F2 - Acquisition Parameters
Date_ 20180526
Time 10.16
INSTRUM spect
PROBHD 5 mm PABBO BB-
PULPROG zgpg30
TD 65536
SOLVENT DMSO
NS 320
DS 4
SWH 18115.941 Hz
FIDRES 0.276427 Hz
AQ 1.8087935 sec
RG 202
DW 27.600 usec
DE 6.50 usec
TE 298.3 K
D1 2.00000000 sec
D11 0.03000000 sec
TD0 1

======== CHANNEL f1 ========
SFO1 75.6462982 MHz
NUC1 13C
P1 10.00 usec
PLW1 30.00000000 W

======== CHANNEL f2 ========
SFO2 300.8112032 MHz
NUC2 1H
CPDPRG[2 waltz16
PCPD2 90.00 usec
PLW2 6.40000010 W
PLW12 0.17778000 W
PLW13 0.14399999 W

F2 - Processing parameters
SI 32768
SF 75.6387350 MHz
WDW EM
SSB 0
LB 1.00 Hz
GB 0
PC 1.40

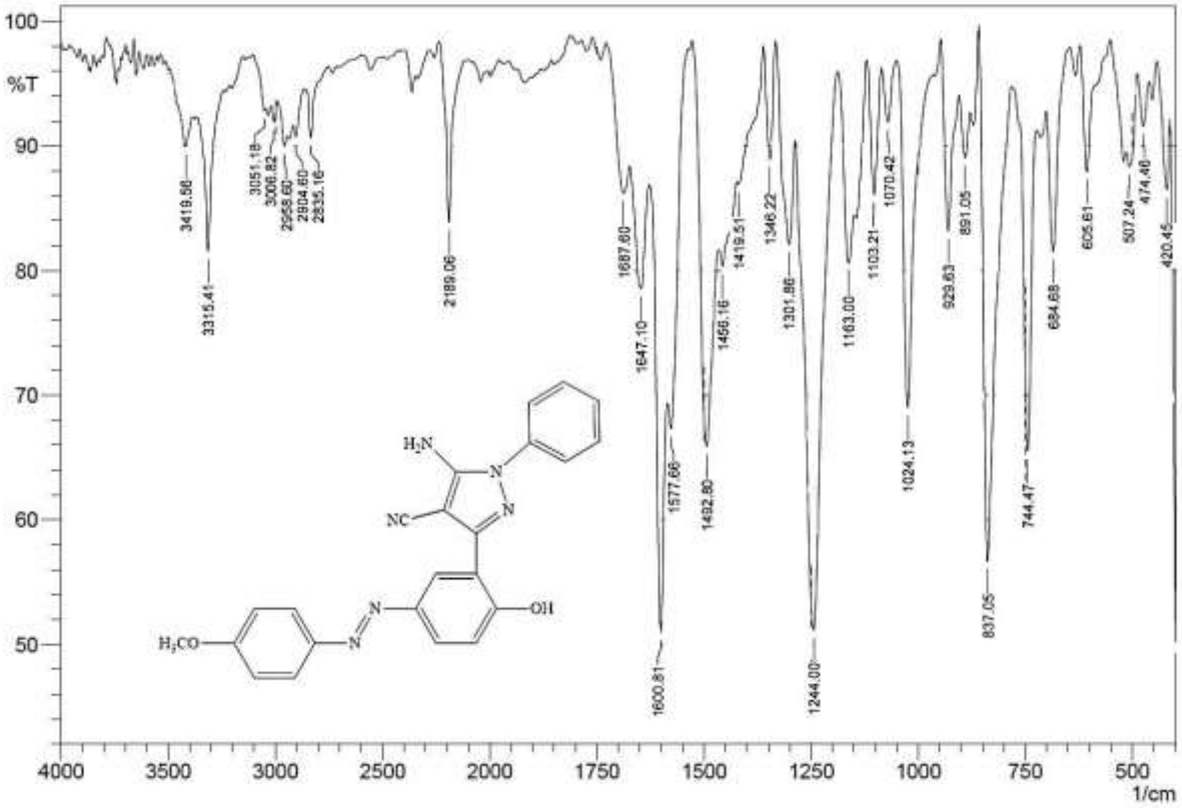

100
%T
90
80
70
60
50
4000
3500
3000
2500
2000
1750
1500
1250
1000
750
500
1/cm
3419.56
3315.41
3051.18
3006.82
2958.60
2904.60
2835.16
2189.06
1687.60
1647.10
1600.81
1577.66
1492.80
1456.16
1419.51
1346.22
1301.86
1244.00
1163.00
1103.21
1070.42
1024.13
929.63
891.05
837.05
744.47
684.68
605.61
507.24
474.46
420.45
H₂N
NC
N
OH
H₃CO

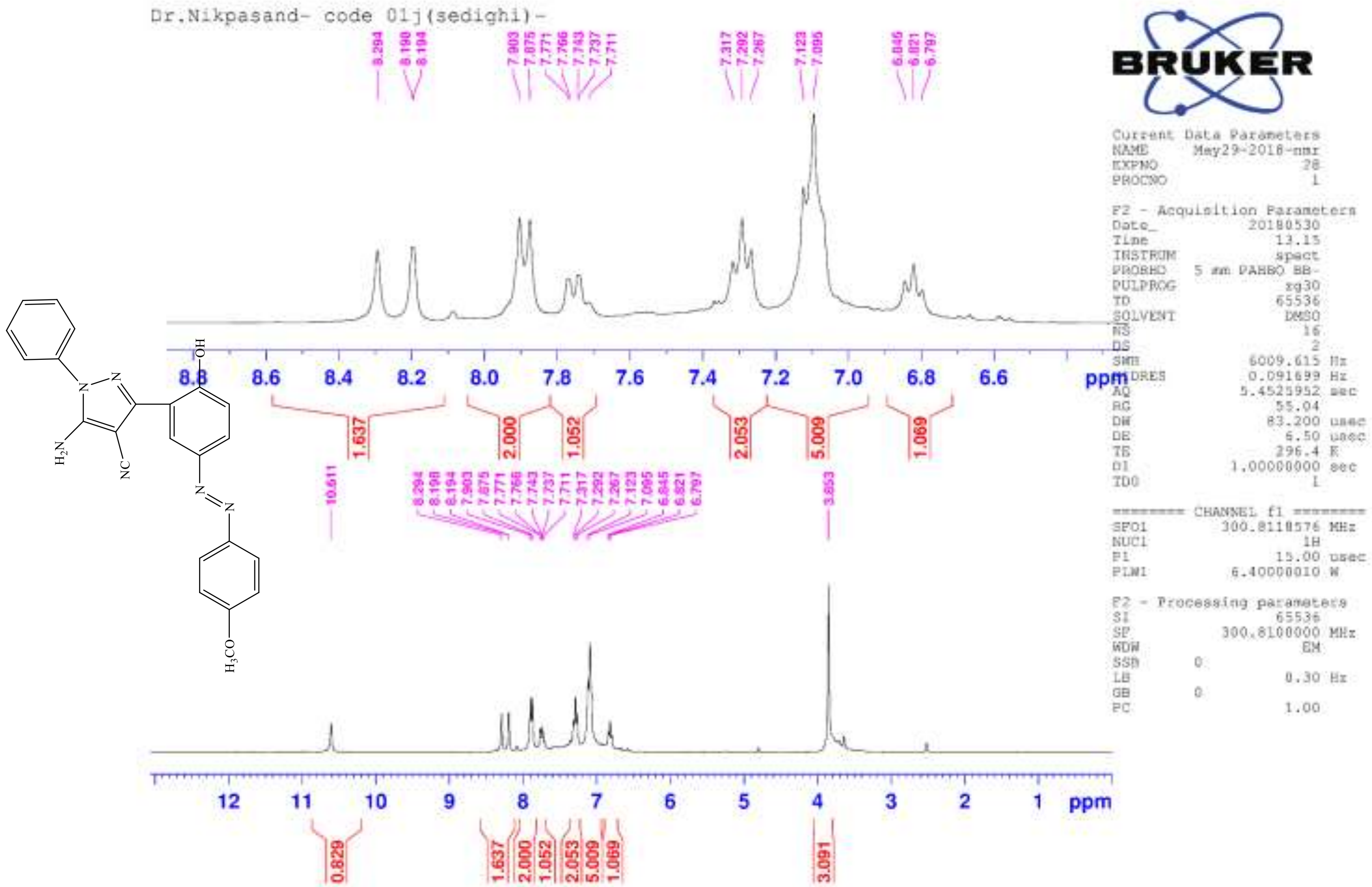
Dr.Nikpasand- code 01j(sedighi)-
8.294
8.198
8.194
7.903
7.875
7.771
7.766
7.743
7.737
7.711
7.317
7.292
7.267
7.123
7.095
6.845
6.821
6.797
10.611
3.853
1.637
2.000
1.052
2.053
5.009
1.069
0.829
3.091
ppm
H2N
NC
N
OH
N=N
H3CO
BRUKER
Current Data Parameters
NAME May29-2018-nmr
EXPNO 28
PROCNO 1
F2 - Acquisition Parameters
Date_ 20180530
Time 13.15
INSTRUM spect
PROBHD 5 mm PABBO BB-
PULPROG zg30
TD 65536
SOLVENT DMSO
NS 16
DS 2
SWH 6009.615 Hz
FIDRES 0.091699 Hz
AQ 5.4525952 sec
RG 55.04
DW 83.200 usec
DE 6.50 usec
TE 296.4 K
D1 1.00000000 sec
TD0 1
======== CHANNEL f1 ========
SFO1 300.8118576 MHz
NUC1 1H
P1 15.00 usec
PLW1 6.40000010 W
F2 - Processing parameters
SI 65536
SF 300.8100000 MHz
WDW EM
SSB 0
LB 0.30 Hz
GB 0
PC 1.00

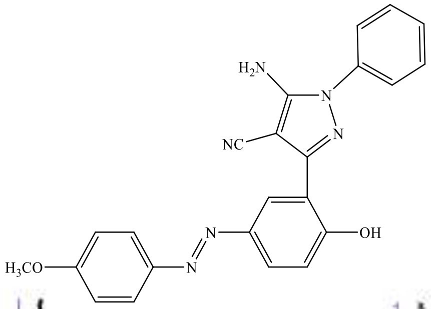
H$_2$N
N
N
NC
N
N
OH
H$_3$CO

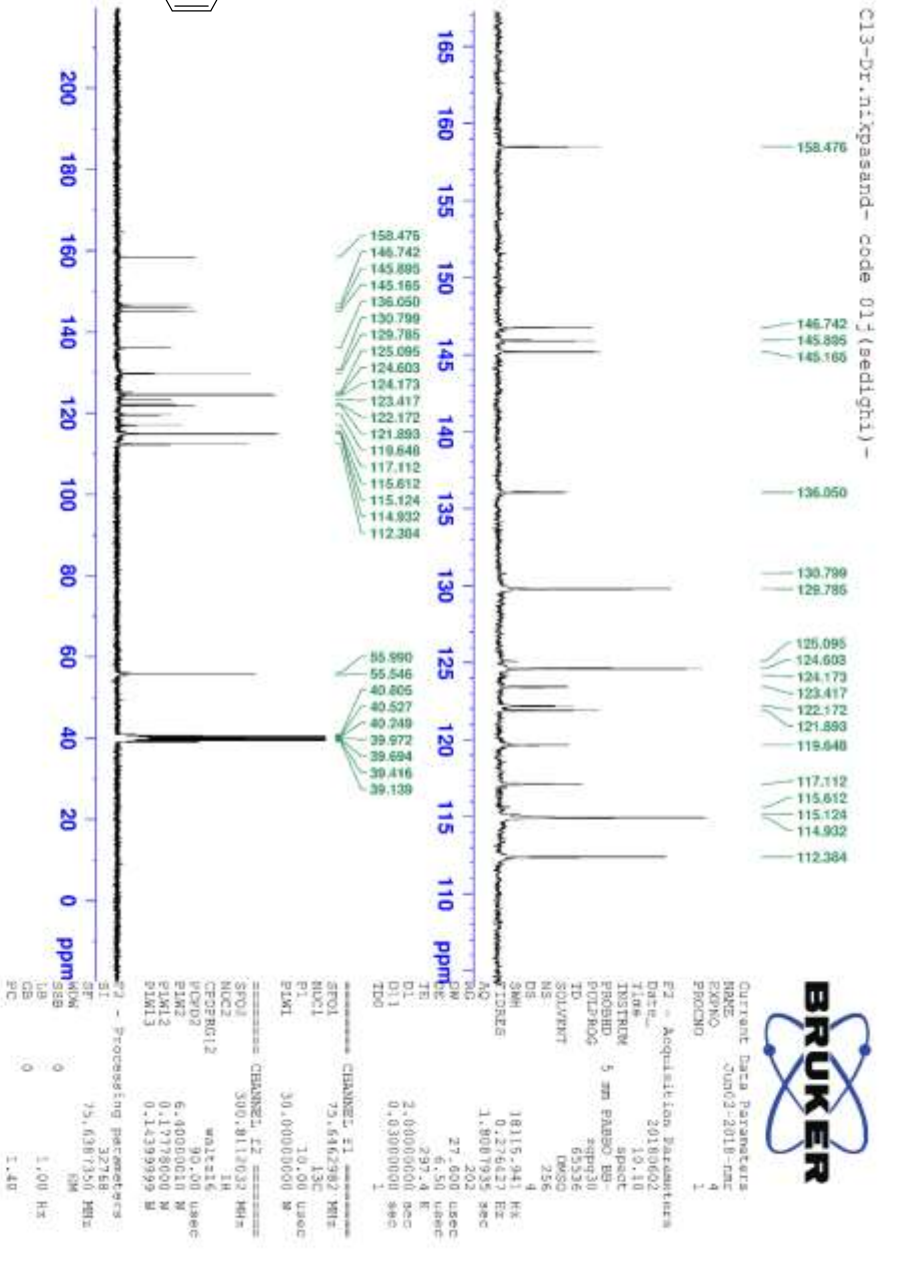
C13-Dr.nikpasand- code 01j(sedighi)-
158.476
146.742
145.895
145.165
136.050
130.799
129.785
125.095
124.603
124.173
123.417
122.172
121.893
119.648
117.112
115.612
115.124
114.932
112.384
55.990
55.546
40.805
40.527
40.249
39.972
39.694
39.416
39.139
ppm
BRUKER

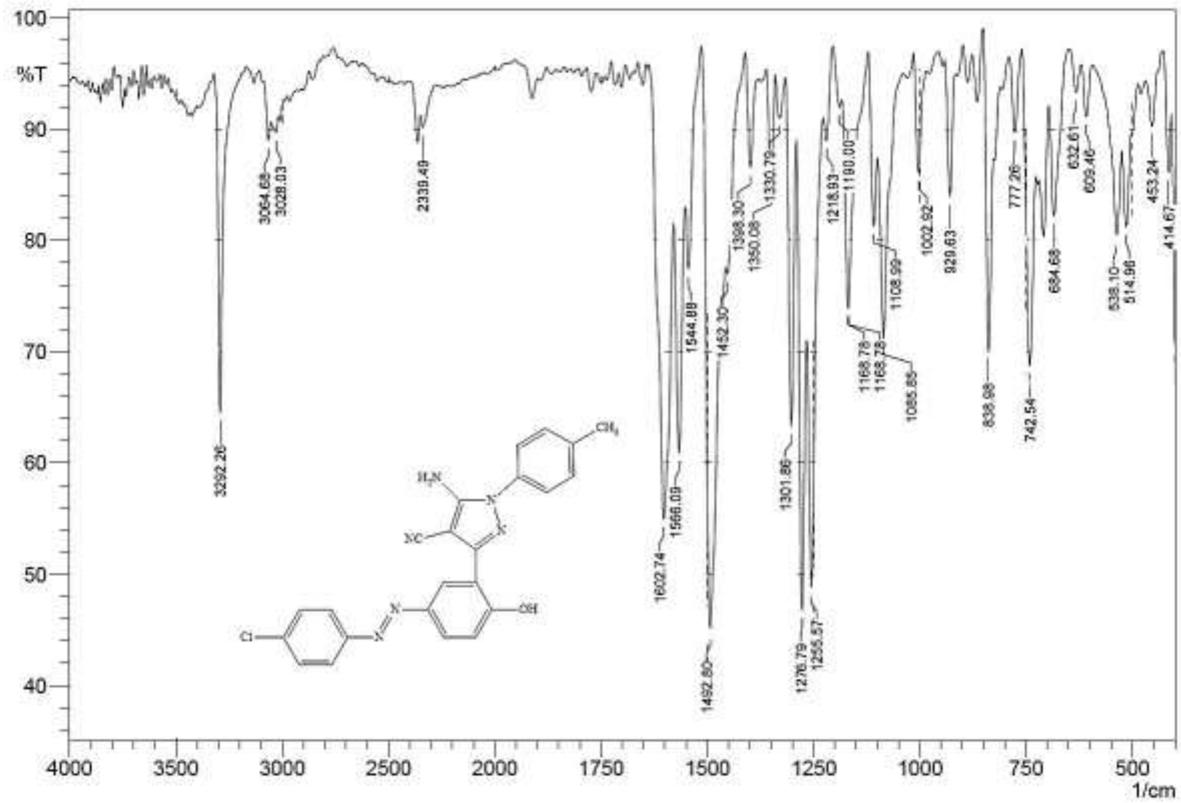

%T
100
90
80
70
60
50
40
4000
3500
3000
2500
2000
1750
1500
1250
1000
750
500
1/cm
3292.26
3064.68
3028.03
2339.49
1602.74
1566.09
1544.88
1492.80
1452.30
1398.30
1350.08
1330.79
1301.86
1276.79
1255.57
1218.93
1180.00
1168.78
1168.78
1108.99
1085.85
1002.92
929.63
838.98
777.26
742.54
684.68
632.61
609.46
538.10
514.96
453.24
414.67
H₂N
NC
OH
Cl
CH₃

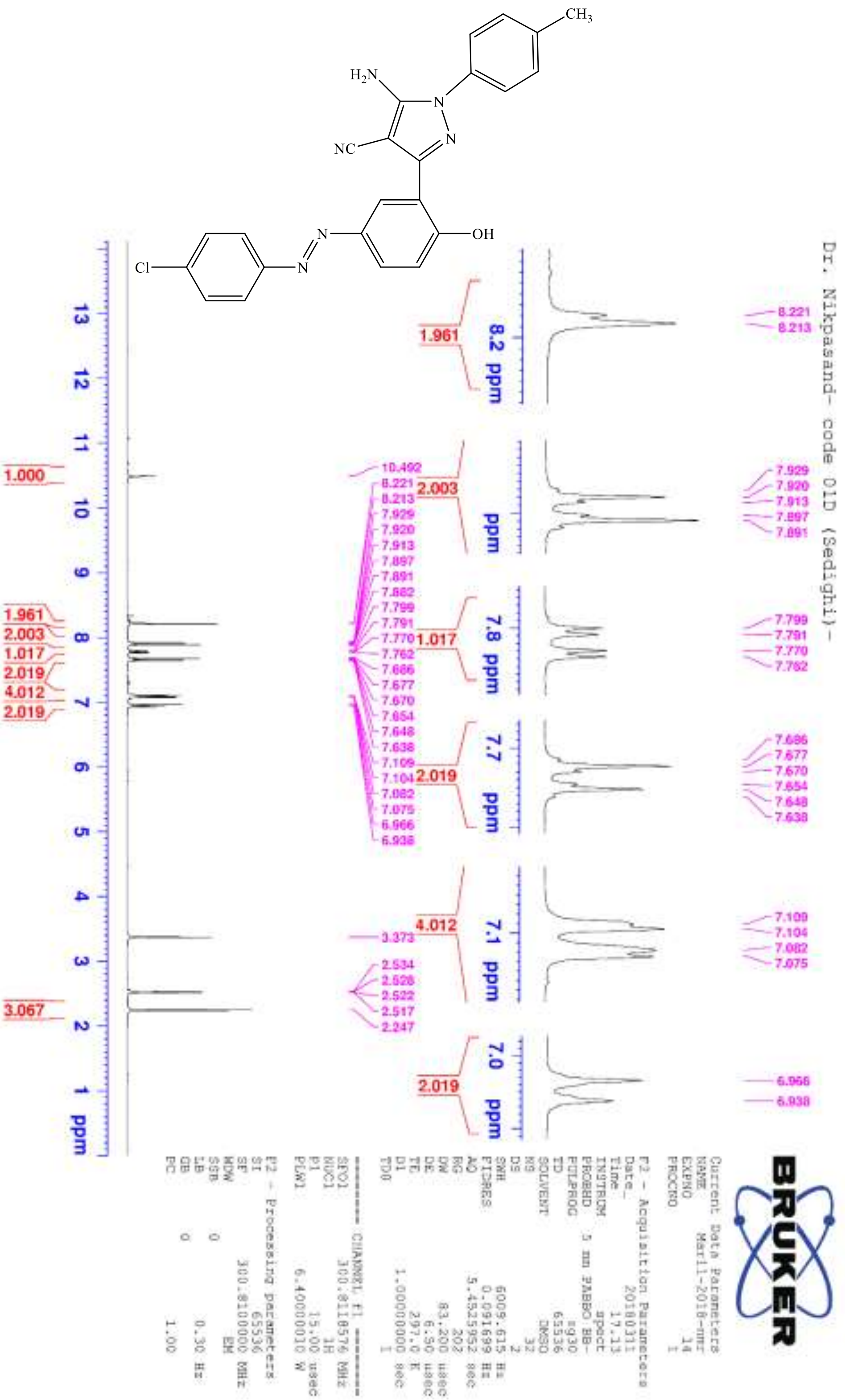

CH3
H2N
NC
OH
Cl
Dr. Nikpasand- code 01D (Sedighi)-
Current Data Parameters
NAME Mar11-2018-nmr
EXPNO 14
PROCNO 1
F2 - Acquisition Parameters
Date_ 20180311
Time 17.13
INSTRUM spect
PROBHD 5 mm PABBO BB-
PULPROG zg30
TD 65536
SOLVENT DMSO
NS 32
DS 2
SWH 6009.615 Hz
FIDRES 0.091699 Hz
AQ 5.4525952 sec
RG 202
DW 83.200 usec
DE 6.50 usec
TE 297.0 K
D1 1.00000000 sec
TD0 1
CHANNEL f1
SFO1 300.8118576 MHz
NUC1 1H
P1 15.00 usec
PLW1 6.40000010 W
F2 - Processing parameters
SI 65536
SF 300.8100000 MHz
WDW EM
SSB 0
LB 0.30 Hz
GB 0
PC 1.00
BRUKER
ppm
10.492
3.373
2.247
1.000
1.961
2.003
1.017
2.019
4.012
3.067

Dr. Nikpasand- code 01D (Sedighi)-

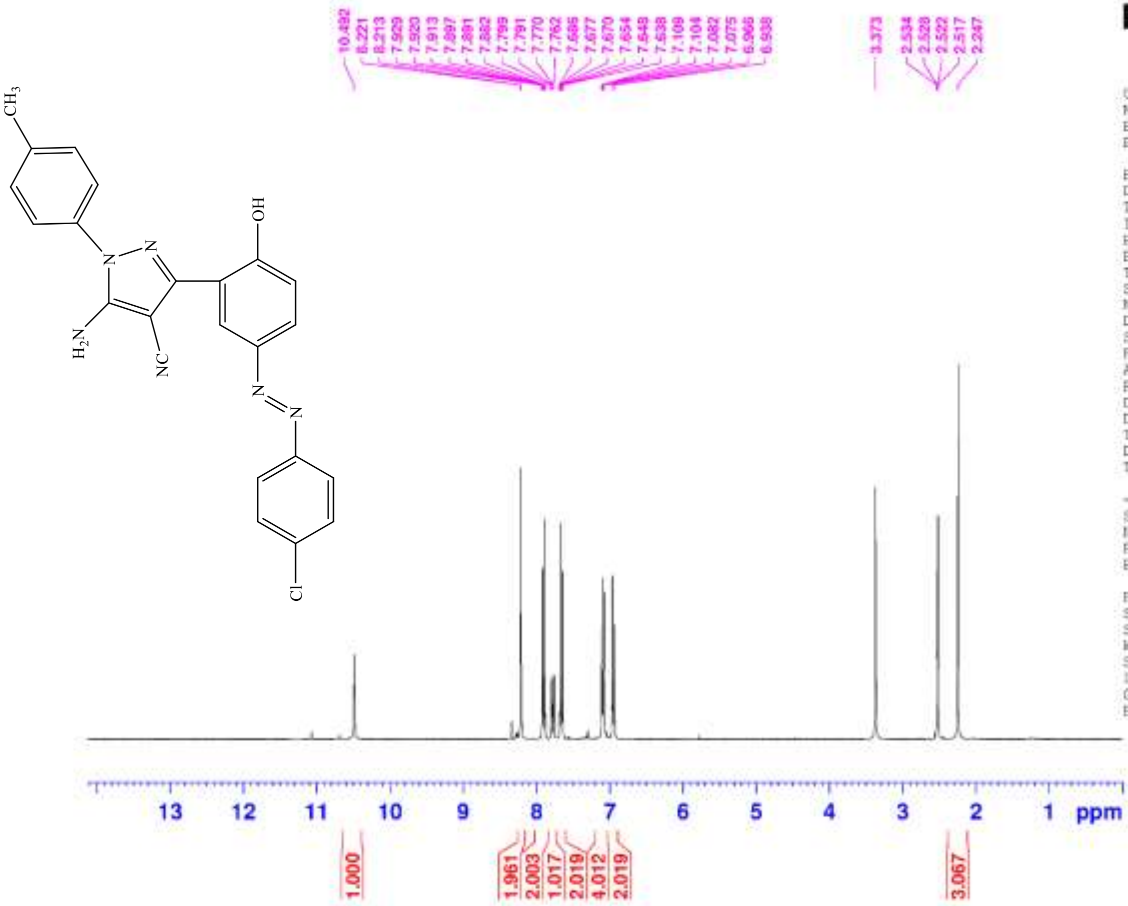

BRUKER

Current Data Parameters
NAME Mar11-2018-nmr
EXPNO 14
PROCNO 1

F2 - Acquisition Parameters
Date_ 20180311
Time 17.13
INSTRUM spect
PROBHD 5 mm PABBO BB-
PULPROG zg30
TD 65536
SOLVENT DMSO
NS 32
DS 2
SWH 6009.615 Hz
FIDRES 0.091699 Hz
AQ 5.4525952 sec
RG 202
DW 83.200 usec
DE 6.50 usec
TE 297.0 K
D1 1.00000000 sec
TD0 1

======== CHANNEL f1 ========
SFO1 300.8118576 MHz
NUC1 1H
P1 15.00 usec
PLW1 6.40000010 W

F2 - Processing parameters
SI 65536
SF 300.8100000 MHz
WDW EM
SSB 0
LB 0.30 Hz
GB 0
PC 1.00

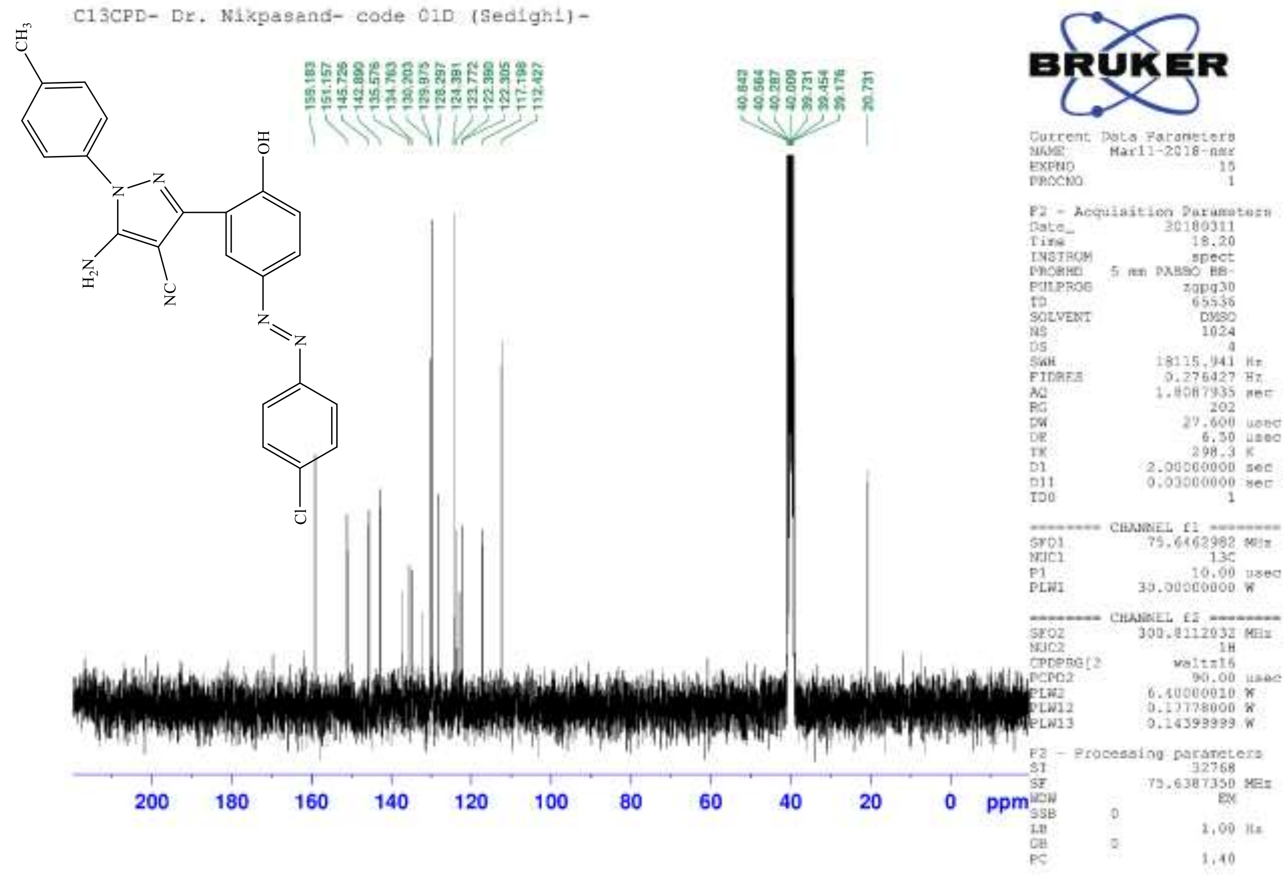
C13CPD- Dr. Nikpasand- code 01D (Sedighi)-
CH3
H2N
NC
OH
N
Cl
159.183
151.157
145.726
142.890
135.576
134.763
130.203
129.975
128.297
124.391
123.772
122.390
122.305
117.198
112.427
40.842
40.564
40.287
40.009
39.731
39.454
39.176
20.731
200 180 160 140 120 100 80 60 40 20 0 ppm
BRUKER
Current Data Parameters
NAME Mar11-2018-nmr
EXPNO 15
PROCNO 1
F2 - Acquisition Parameters
Date_ 20180311
Time 18.20
INSTRUM spect
PROBHD 5 mm PABBO BB-
PULPROG zgpg30
TD 65536
SOLVENT DMSO
NS 1024
DS 4
SWH 18115.941 Hz
FIDRES 0.276427 Hz
AQ 1.8087935 sec
RG 202
DW 27.600 usec
DE 6.50 usec
TE 298.3 K
D1 2.00000000 sec
D11 0.03000000 sec
TD0 1
======== CHANNEL f1 ========
SFO1 75.6462982 MHz
NUC1 13C
P1 10.00 usec
PLW1 30.00000000 W
======== CHANNEL f2 ========
SFO2 300.8112032 MHz
NUC2 1H
CPDPRG[2 waltz16
PCPD2 90.00 usec
PLW2 6.40000010 W
PLW12 0.17778000 W
PLW13 0.14399999 W
F2 - Processing parameters
SI 32768
SF 75.6387350 MHz
WDW EM
SSB 0
LB 1.00 Hz
GB 0
PC 1.40

C13CPD- Dr. Nikpasand- code 01D (Sedighi)-

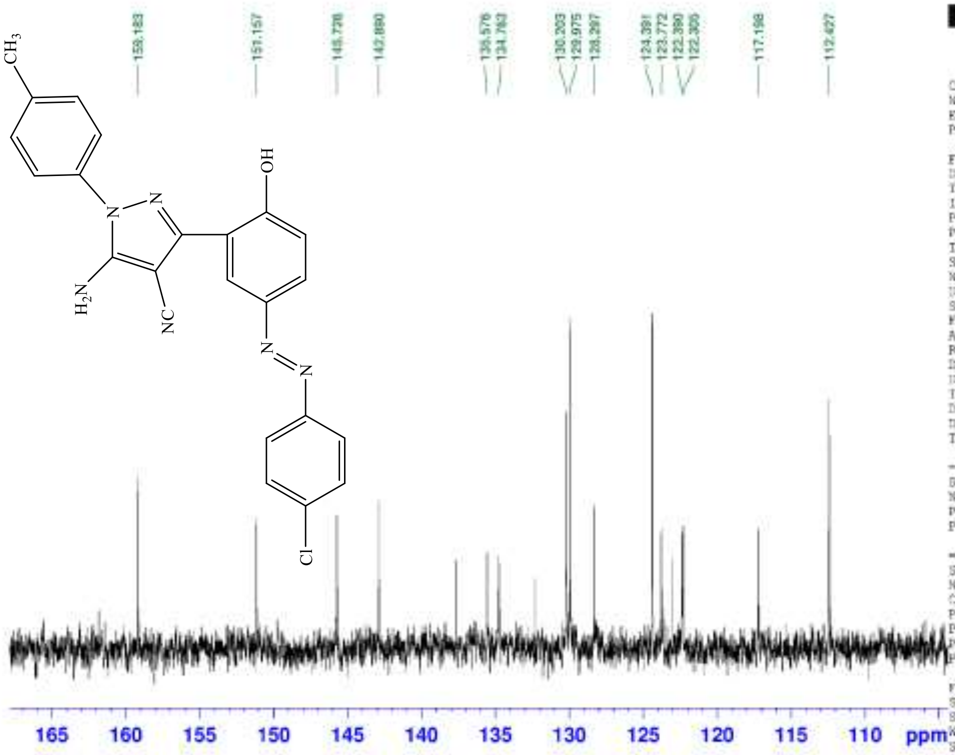

BRUKER

```
Current Data Parameters
NAME      Mar11-2018-nmr
EXPNO                 15
PROCNO                 1

F2 - Acquisition Parameters
Date_           20180311
Time               18.20
INSTRUM            spect
PROBHD   5 mm PABBO BB-
PULPROG           zgpg30
TD                 65536
SOLVENT             DMSO
NS                  1024
DS                     4
SWH            18115.941 Hz
FIDRES          0.276427 Hz
AQ             1.8087935 sec
RG                   202
DW                27.600 usec
DE                  6.50 usec
TE                 298.3 K
D1            2.00000000 sec
D11           0.03000000 sec
TD0                    1

======== CHANNEL f1 ========
SFO1         75.6462982 MHz
NUC1                 13C
P1                 10.00 usec
PLW1         30.00000000 W

======== CHANNEL f2 ========
SFO2        300.8112032 MHz
NUC2                  1H
CPDPRG[2         waltz16
PCPD2              90.00 usec
PLW2          6.40000010 W
PLW12         0.17778000 W
PLW13         0.14399999 W

F2 - Processing parameters
SI                 32768
SF            75.6387350 MHz
WDW                   EM
SSB      0
LB                  1.00 Hz
GB       0
PC                  1.40
```

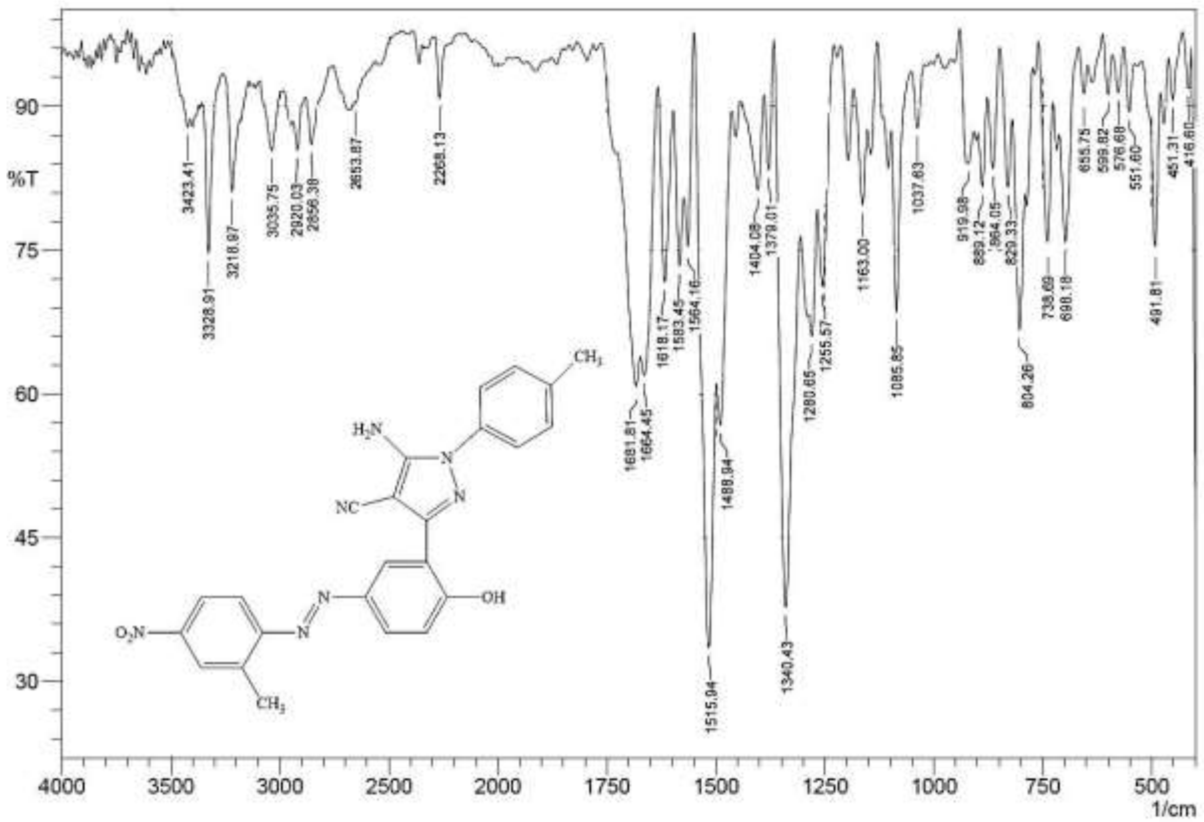

%T
90
75
60
45
30
4000
3500
3000
2500
2000
1750
1500
1250
1000
750
500
1/cm
3423.41
3328.91
3218.97
3035.75
2920.03
2856.38
2653.87
2268.13
1681.81
1664.45
1618.17
1583.45
1564.16
1515.94
1488.94
1404.08
1379.01
1340.43
1280.65
1255.57
1163.00
1085.85
1037.63
919.98
889.12
864.05
829.33
804.26
738.69
698.18
655.75
599.82
576.68
551.60
491.81
451.31
416.60
H₂N
CH₃
NC
N
OH
O₂N
CH₃

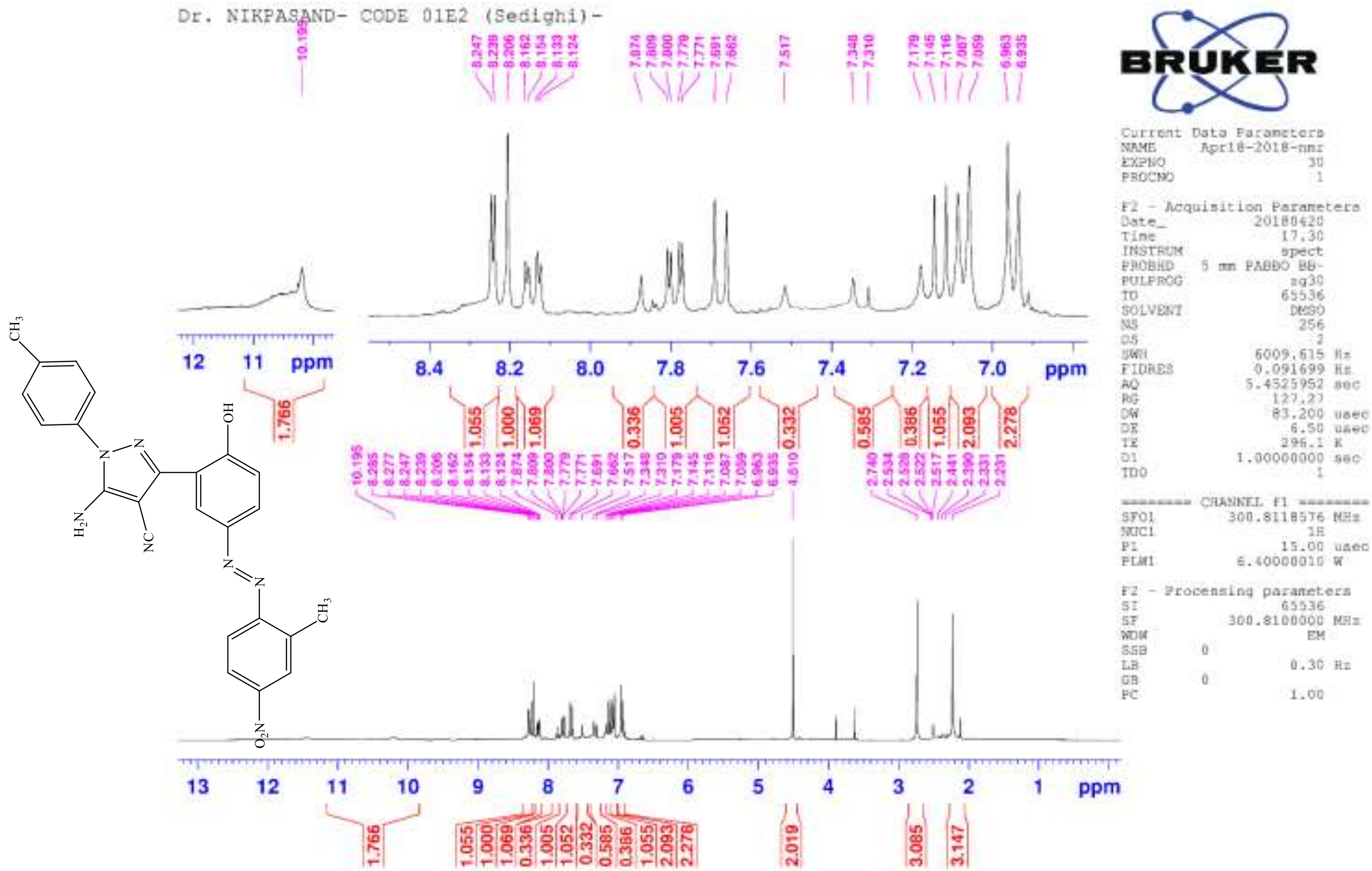
Dr. NIKPASAND- CODE 01E2 (Sedighi)-
BRUKER
Current Data Parameters
NAME Apr18-2018-nmr
EXPNO 30
PROCNO 1
F2 - Acquisition Parameters
Date_ 20180420
Time 17.30
INSTRUM spect
PROBHD 5 mm PABBO BB-
PULPROG zg30
TD 65536
SOLVENT DMSO
NS 256
DS 2
SWH 6009.615 Hz
FIDRES 0.091699 Hz
AQ 5.4525952 sec
RG 127.27
DW 83.200 usec
DE 6.50 usec
TE 296.1 K
D1 1.00000000 sec
TD0 1
======== CHANNEL f1 ========
SFO1 300.8118576 MHz
NUC1 1H
P1 15.00 usec
PLW1 6.40000010 W
F2 - Processing parameters
SI 65536
SF 300.8100000 MHz
WDW EM
SSB 0
LB 0.30 Hz
GB 0
PC 1.00

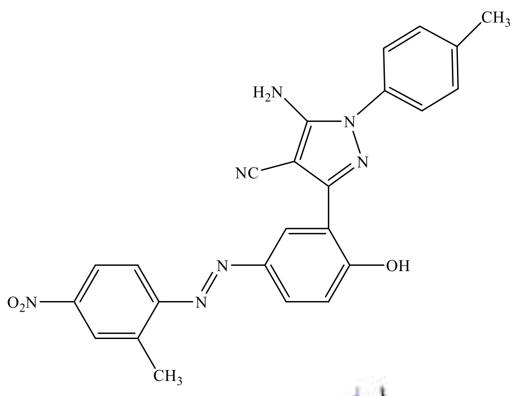
CH3
H2N
N
N
NC
OH
N
N
O2N
CH3

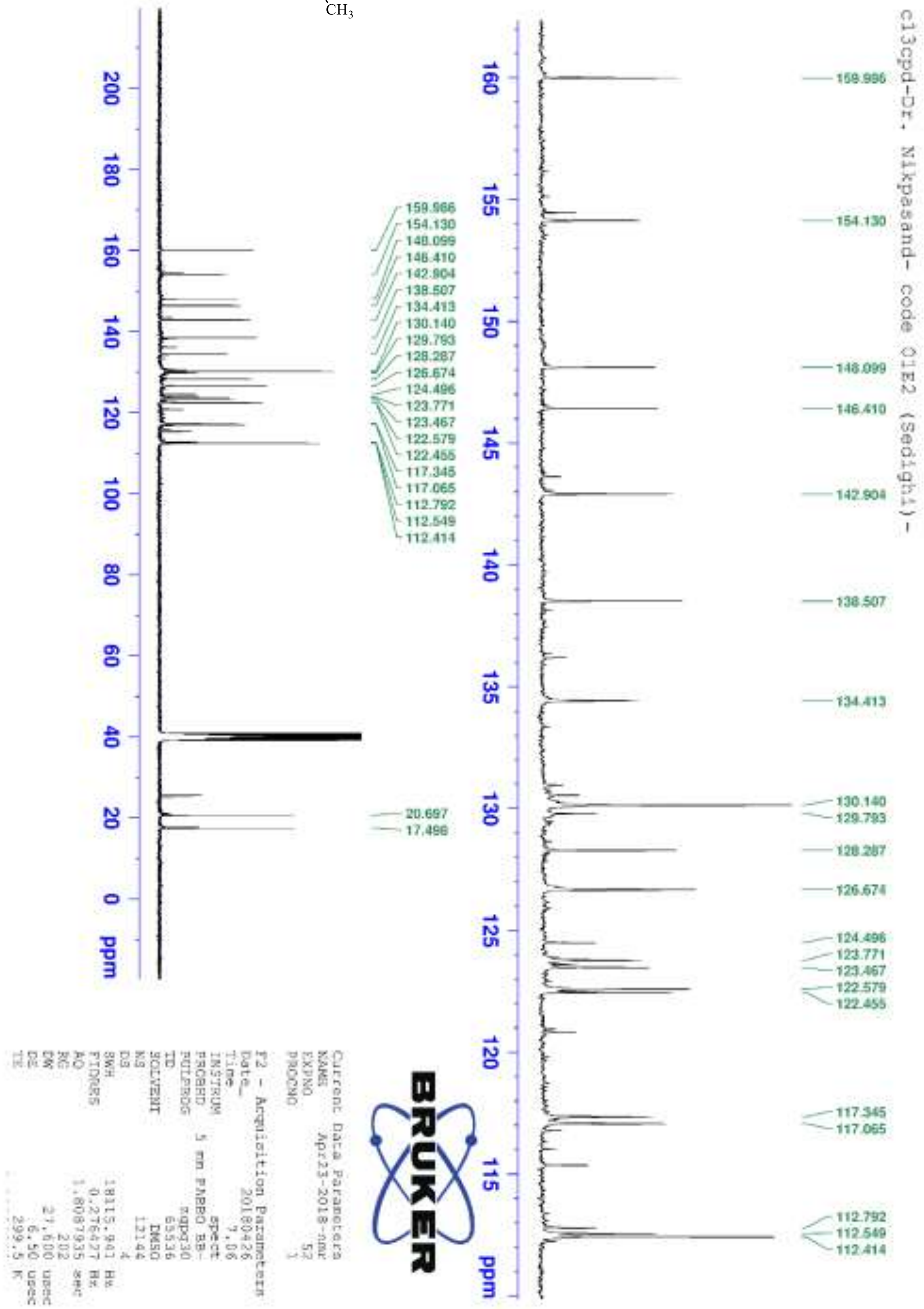
c13cpd-Dr. Nikpasand- code 01E2 (Sedighi)-
159.986
154.130
148.099
146.410
142.904
138.507
134.413
130.140
129.793
128.287
126.674
124.496
123.771
123.467
122.579
122.455
117.345
117.065
112.792
112.549
112.414
20.697
17.498
ppm
Current Data Parameters
NAME Apr23-2018-nmr
EXPNO 52
PROCNO 1
F2 - Acquisition Parameters
Date_ 20180426
Time 7.06
INSTRUM spect
PROBHD 5 mm PABBO BB-
PULPROG zgpg30
TD 65536
SOLVENT DMSO
NS 12144
DS 4
SWH 18115.941 Hz
FIDRES 0.276427 Hz
AQ 1.8087935 sec
RG 202
DW 27.600 usec
DE 6.50 usec
TE 299.5 K

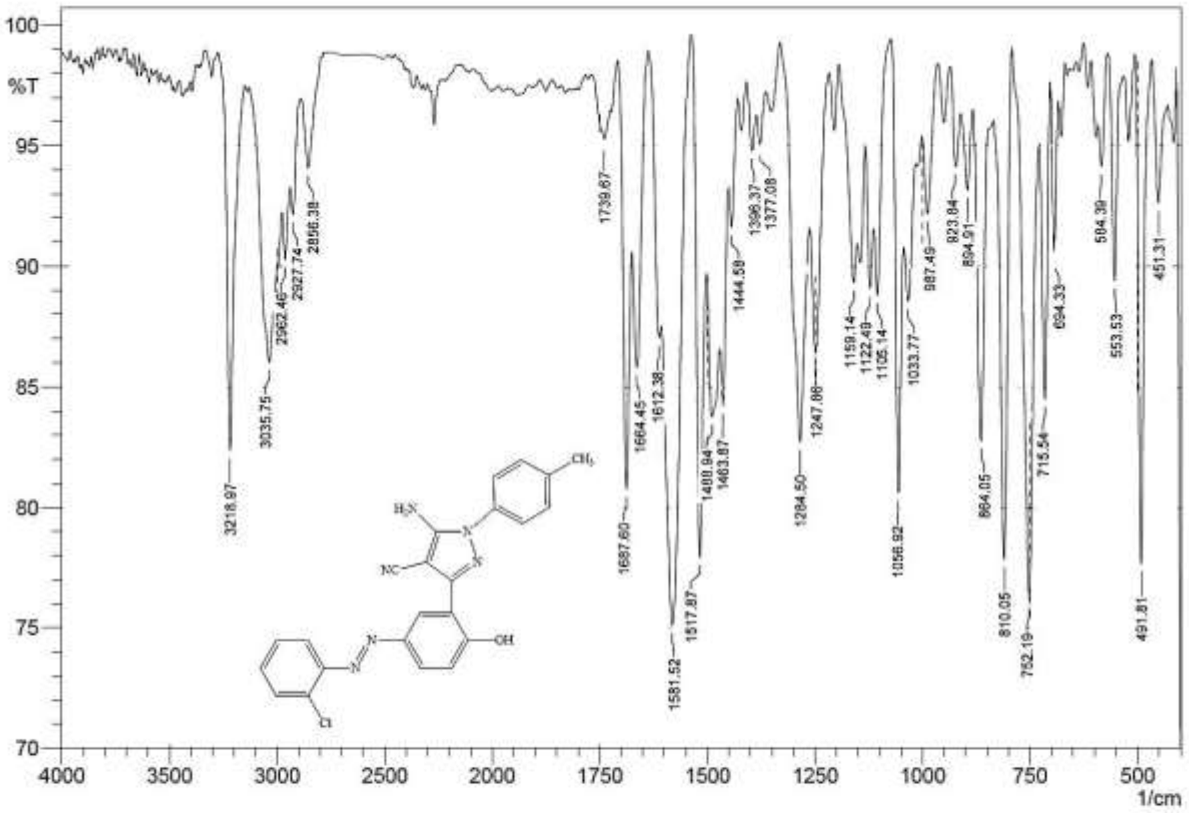

%T
1/cm
H₂N
NC
CH₃
OH
Cl

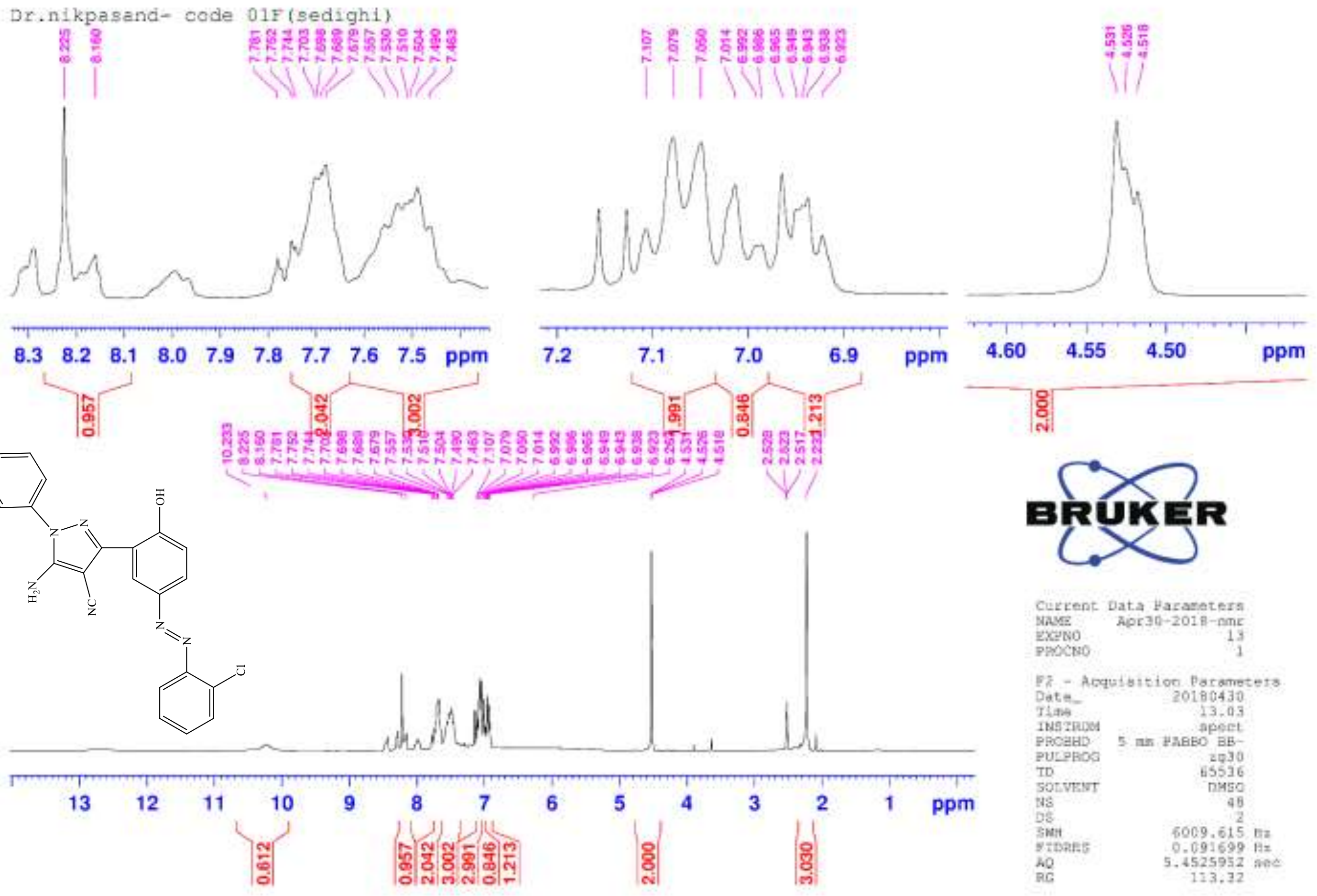
Dr.nikpasand- code 01F(sedighi)
BRUKER
Current Data Parameters
NAME Apr30-2018-nmr
EXPNO 13
PROCNO 1
F2 - Acquisition Parameters
Date_ 20180430
Time 13.03
INSTRUM spect
PROBHD 5 mm PABBO BB-
PULPROG zg30
TD 65536
SOLVENT DMSO
NS 48
DS 2
SWH 6009.615 Hz
FIDRES 0.091699 Hz
AQ 5.4525952 sec
RG 113.32

C13-Dr.Nikpasand- code 01F(sedighi)-

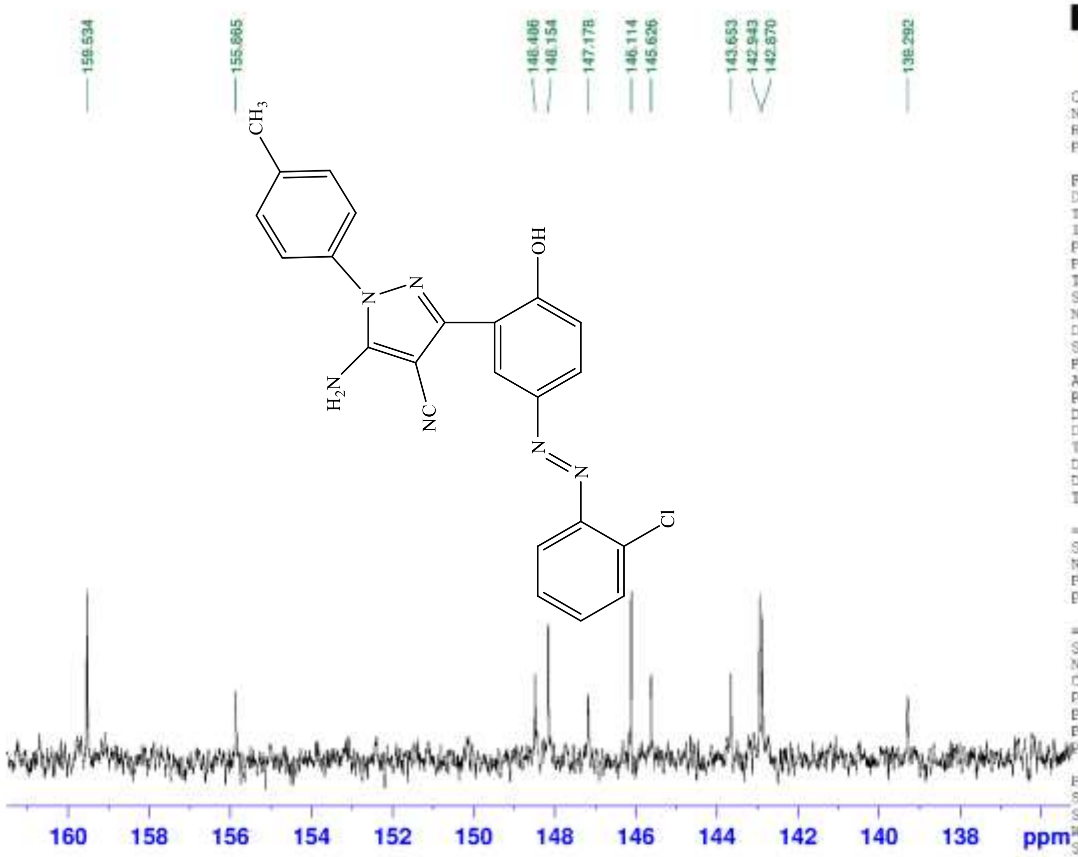


```
Current Data Parameters
NAME        May06-2018-nmr
EXPNO                   11
PROCNO                   1

F2 - Acquisition Parameters
Date_             20180506
Time                 13.13
INSTRUM              spect
PROBHD    5 mm PABBO BB-
PULPROG            zgpg30
TD                   65536
SOLVENT               DMSO
NS                    1024
DS                       4
SWH              18115.941 Hz
FIDRES            0.276427 Hz
AQ               1.8087935 sec
RG                     202
DW                  27.600 usec
DE                    6.50 usec
TE                   297.0 K
D1              2.00000000 sec
D11             0.03000000 sec
TD0                      1

======== CHANNEL f1 ========
SFO1            75.6462982 MHz
NUC1                   13C
P1                   10.00 usec
PLW1           30.00000000 W

======== CHANNEL f2 ========
SFO2           300.8112032 MHz
NUC2                    1H
CPDPRG[2           waltz16
PCPD2                90.00 usec
PLW2            6.40000010 W
PLW12           0.17778000 W
PLW13           0.14399999 W

F2 - Processing parameters
SI                   32768
SF              75.6387350 MHz
WDW                     EM
SSB      0
LB                    1.00 Hz
GB       0
PC                    1.40
```

C13-Dr.Nikpasand- code 01F(sedighi)-

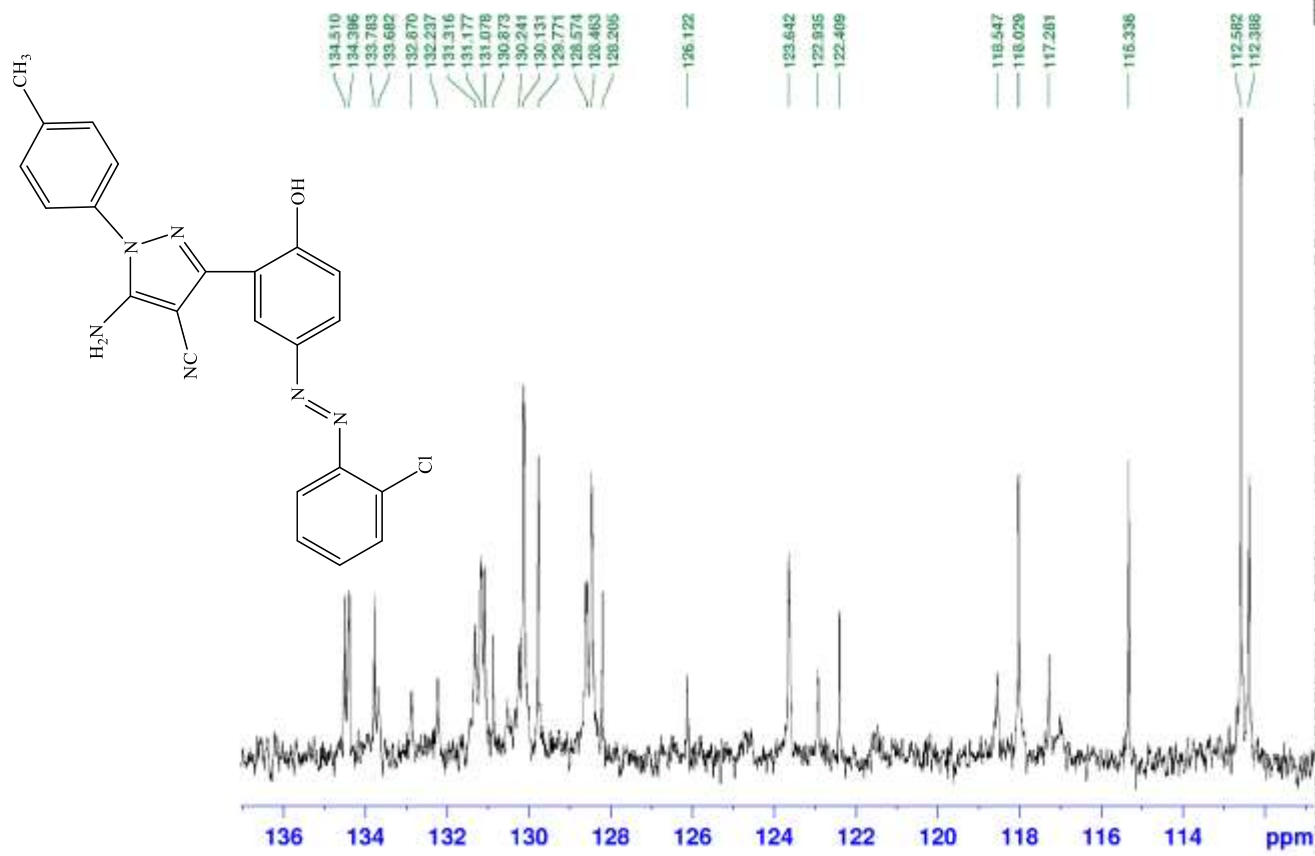

BRUKER

Current Data Parameters
NAME May06-2018-nmr
EXPNO 11
PROCNO 1

F2 - Acquisition Parameters
Date_ 20180506
Time 13.13
INSTRUM spect
PROBHD 5 mm PABBO BB-
PULPROG zgpg30
TD 65536
SOLVENT DMSO
NS 1024
DS 4
SWH 18115.941 Hz
FIDRES 0.276427 Hz
AQ 1.8087935 sec
RG 202
DW 27.600 usec
DE 6.50 usec
TE 297.0 K
D1 2.00000000 sec
D11 0.03000000 sec
TD0 1

======== CHANNEL f1 ========
SFO1 75.6462982 MHz
NUC1 13C
P1 10.00 usec
PLW1 30.00000000 W

======== CHANNEL f2 ========
SFO2 300.8112032 MHz
NUC2 1H
CPDPRG[2 waltz16
PCPD2 90.00 usec
PLW2 6.40000010 W
PLW12 0.17778000 W
PLW13 0.14399999 W

F2 - Processing parameters
SI 32768
SF 75.6387350 MHz
WDW EM
SSB 0
LB 1.00 Hz
GB 0
PC 1.40

C13-Dr.Nikpasand- code 01F(sedighi)-

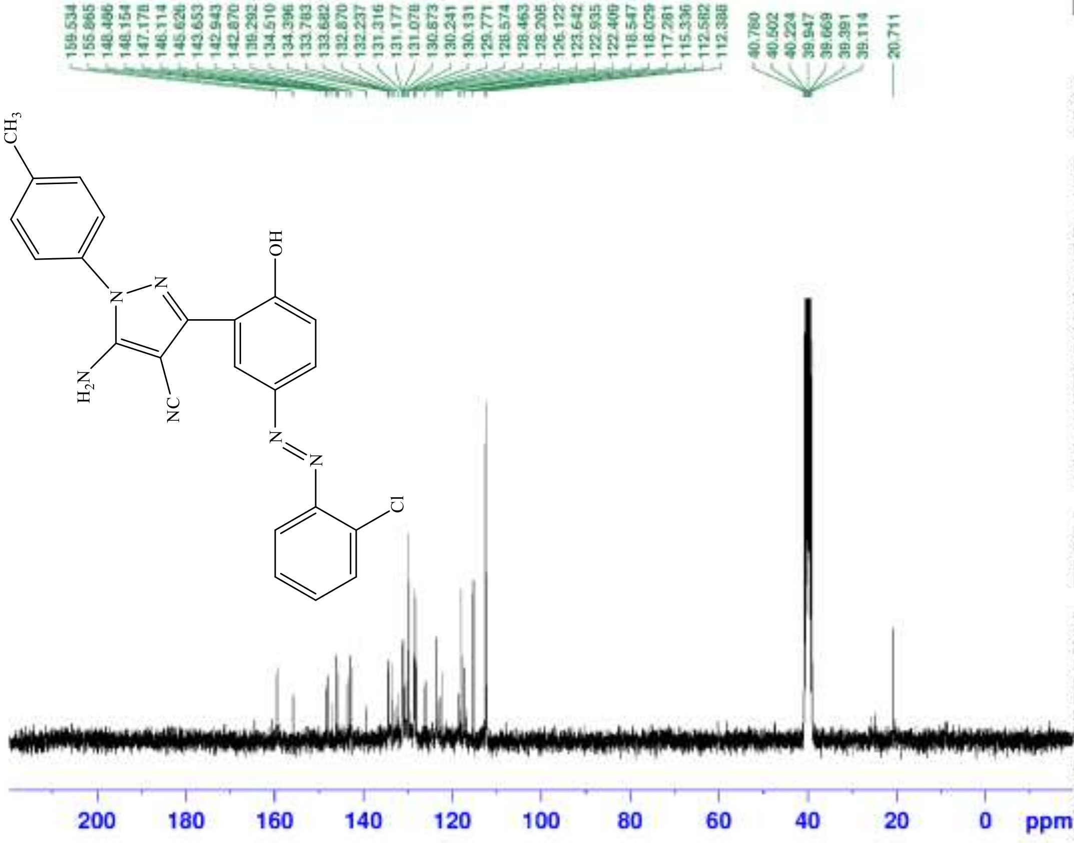

BRUKER

Current Data Parameters
NAME May06-2018-nmr
EXPNO 11
PROCNO 1

F2 - Acquisition Parameters
Date_ 20180506
Time 13.13
INSTRUM spect
PROBHD 5 mm PABBO BB-
PULPROG zgpg30
TD 65536
SOLVENT DMSO
NS 1024
DS 4
SWH 18115.941 Hz
FIDRES 0.276427 Hz
AQ 1.8087935 sec
RG 202
DW 27.600 usec
DE 6.50 usec
TE 297.0 K
D1 2.00000000 sec
D11 0.03000000 sec
TD0 1

======== CHANNEL f1 ========
SFO1 75.6462982 MHz
NUC1 13C
P1 10.00 usec
PLW1 30.00000000 W

======== CHANNEL f2 ========
SFO2 300.8112032 MHz
NUC2 1H
CPDPRG[2 waltz16
PCPD2 90.00 usec
PLW2 6.40000010 W
PLW12 0.17778000 W
PLW13 0.14399999 W

F2 - Processing parameters
SI 32768
SF 75.6387350 MHz
WDW EM
SSB 0
LB 1.00 Hz
GB 0
PC 1.40

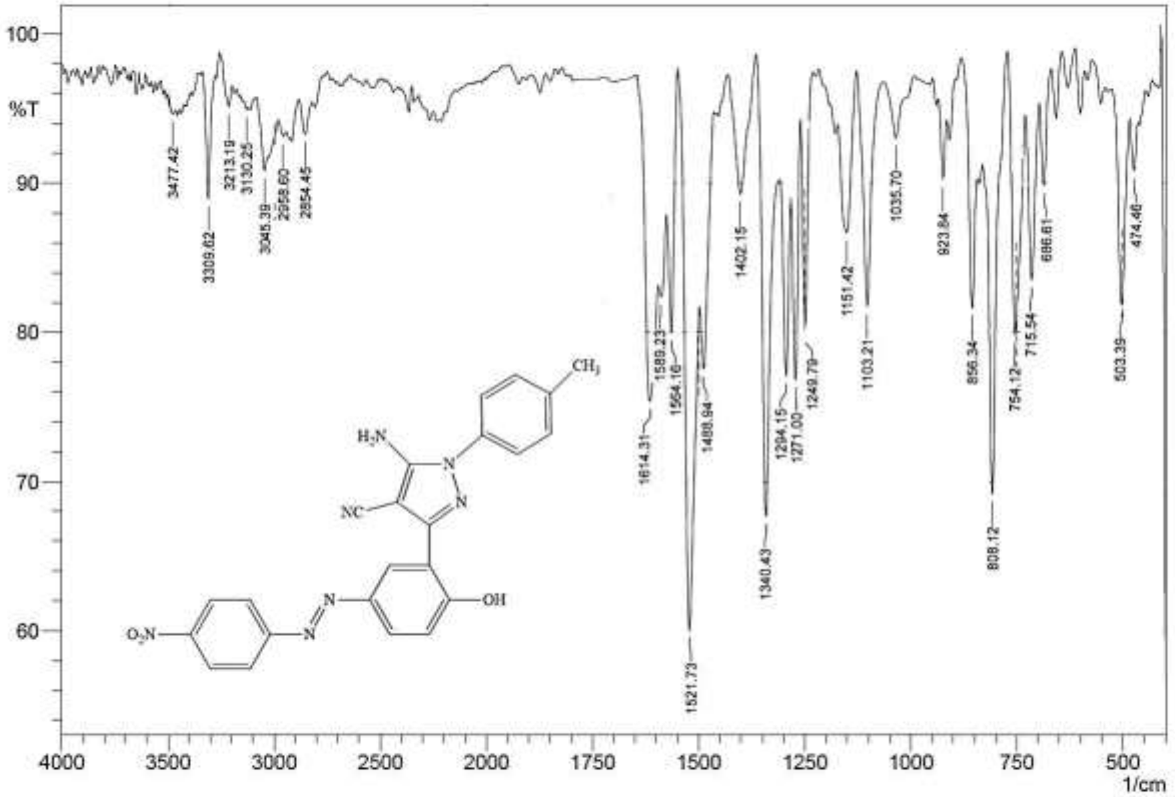

100
%T
90
80
70
60
3477.42
3309.62
3213.19
3130.25
3045.39
2958.60
2854.45
1614.31
1589.23
1564.16
1521.73
1488.94
1402.15
1340.43
1294.15
1271.00
1249.79
1151.42
1103.21
1035.70
923.84
856.34
808.12
754.12
715.54
686.61
503.39
474.46
4000
3500
3000
2500
2000
1750
1500
1250
1000
750
500
1/cm
CH3
H2N
N
N
NC
N
N
OH
O2N

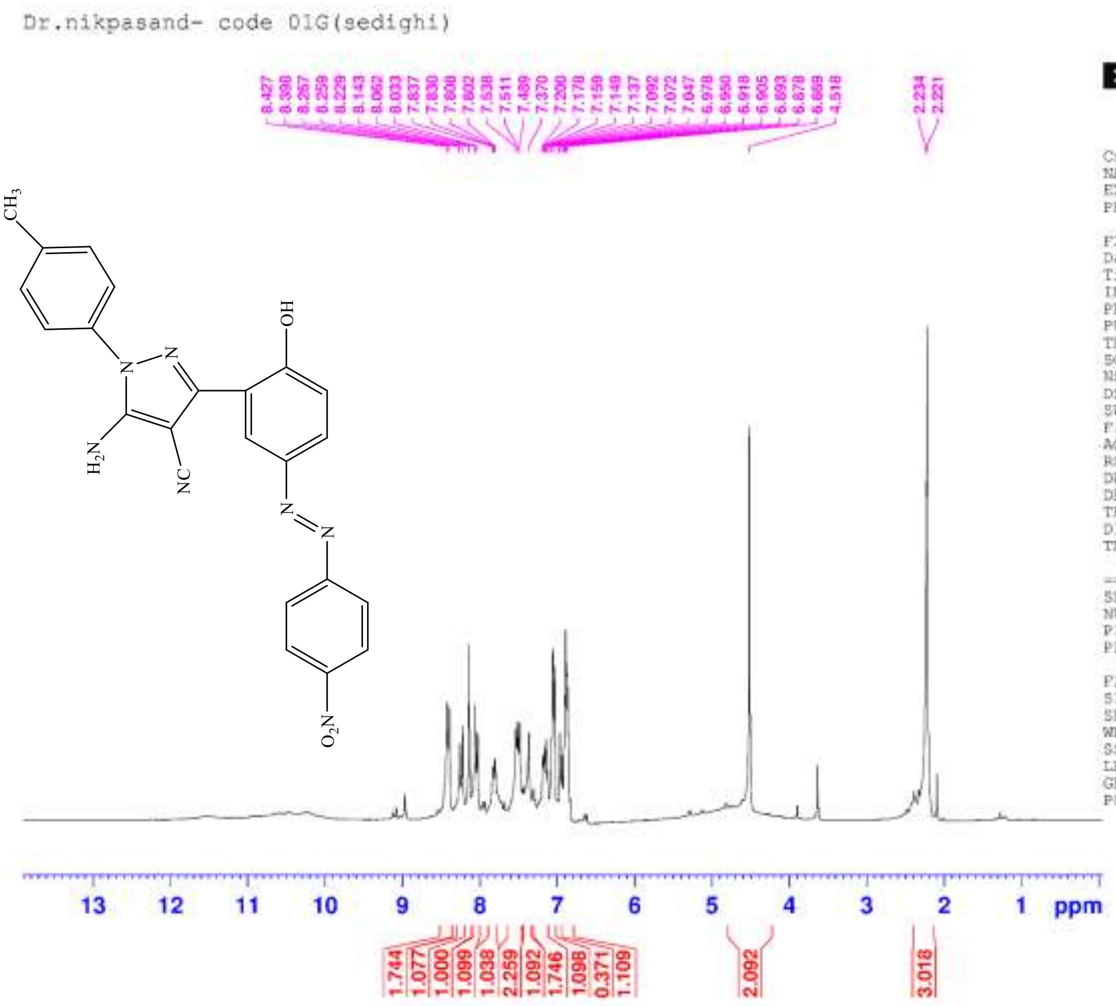


```
Current Data Parameters
NAME        Apr30-2018-nmr
EXPNO                   12
PROCNO                   1

F2 - Acquisition Parameters
Date_             20180430
Time                 12.53
INSTRUM              spect
PROBHD   5 mm PABBO BB-
PULPROG               zg30
TD                   65536
SOLVENT               DMSO
NS                      48
DS                       2
SWH            6009.615 Hz
FIDRES         0.091699 Hz
AQ            5.4525952 sec
RG                   99.22
DW               83.200 usec
DE                 6.50 usec
TE                 295.6 K
D1           1.00000000 sec
TD0                      1

======== CHANNEL f1 ========
SFO1        300.8118576 MHz
NUC1                    1H
P1                15.00 usec
PLW1         6.40000010 W

F2 - Processing parameters
SI                   65536
SF          300.8100000 MHz
WDW                     EM
SSB      0
LB                 0.30 Hz
GB       0
PC                    1.00
```

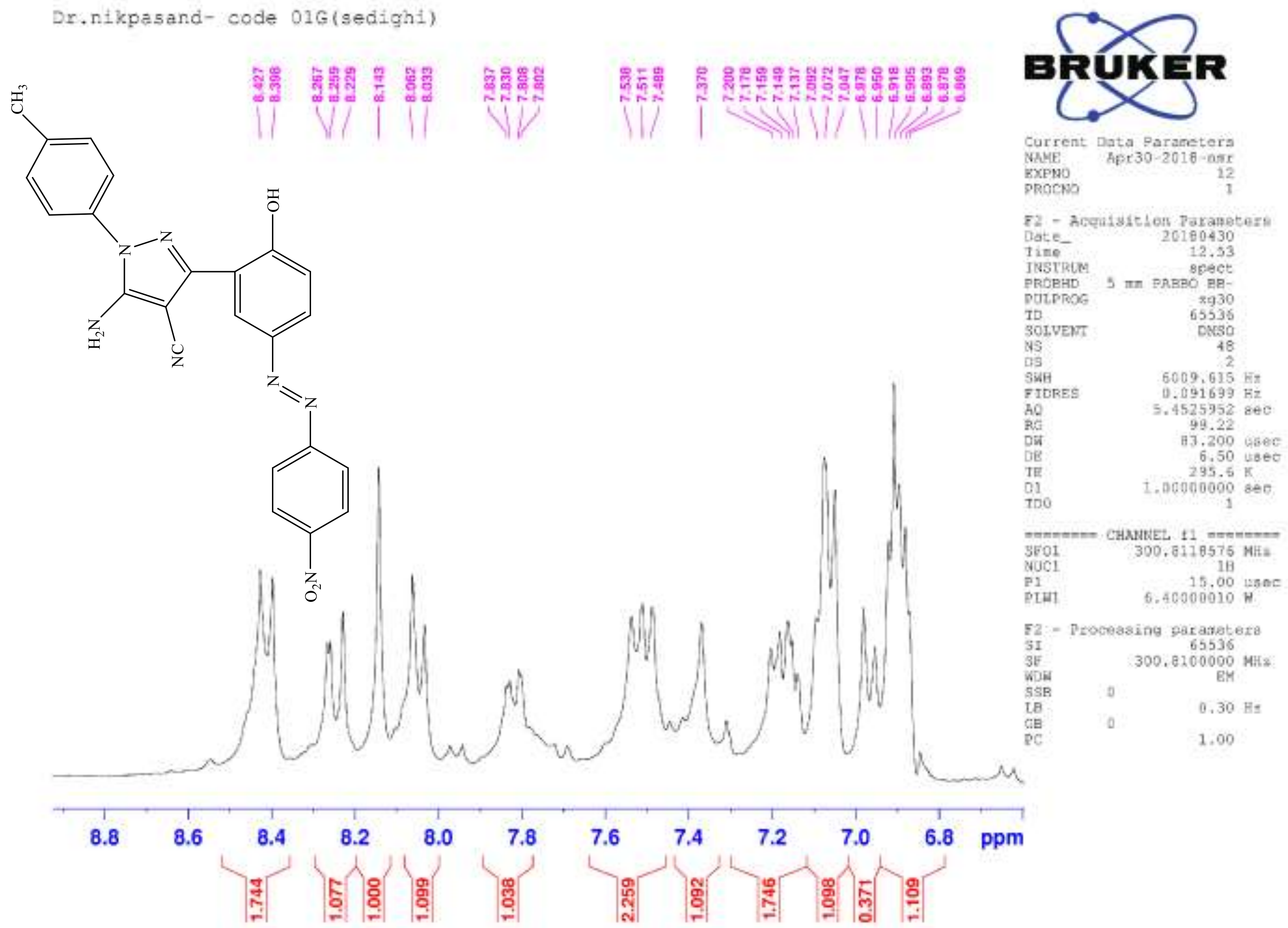
Dr.nikpasand- code 01G(sedighi)
CH3
H2N
N
N
NC
OH
N
N
O2N
8.427
8.398
8.267
8.259
8.229
8.143
8.062
8.033
7.837
7.830
7.808
7.802
7.538
7.511
7.489
7.370
7.200
7.178
7.159
7.149
7.137
7.092
7.072
7.047
6.978
6.950
6.918
6.905
6.893
6.878
6.869
BRUKER
Current Data Parameters
NAME Apr30-2018-nmr
EXPNO 12
PROCNO 1
F2 - Acquisition Parameters
Date_ 20180430
Time 12.53
INSTRUM spect
PROBHD 5 mm PABBO BB-
PULPROG zg30
TD 65536
SOLVENT DMSO
NS 48
DS 2
SWH 6009.615 Hz
FIDRES 0.091699 Hz
AQ 5.4525952 sec
RG 99.22
DW 83.200 usec
DE 6.50 usec
TE 295.6 K
D1 1.00000000 sec
TD0 1
======== CHANNEL f1 ========
SFO1 300.8118576 MHz
NUC1 1H
P1 15.00 usec
PLW1 6.40000010 W
F2 - Processing parameters
SI 65536
SF 300.8100000 MHz
WDW EM
SSB 0
LB 0.30 Hz
GB 0
PC 1.00
8.8
8.6
8.4
8.2
8.0
7.8
7.6
7.4
7.2
7.0
6.8
ppm
1.744
1.077
1.000
1.099
1.038
2.259
1.092
1.746
1.098
0.371
1.109

C13-Dr.Nikpasand- code 01G(sedighi)-

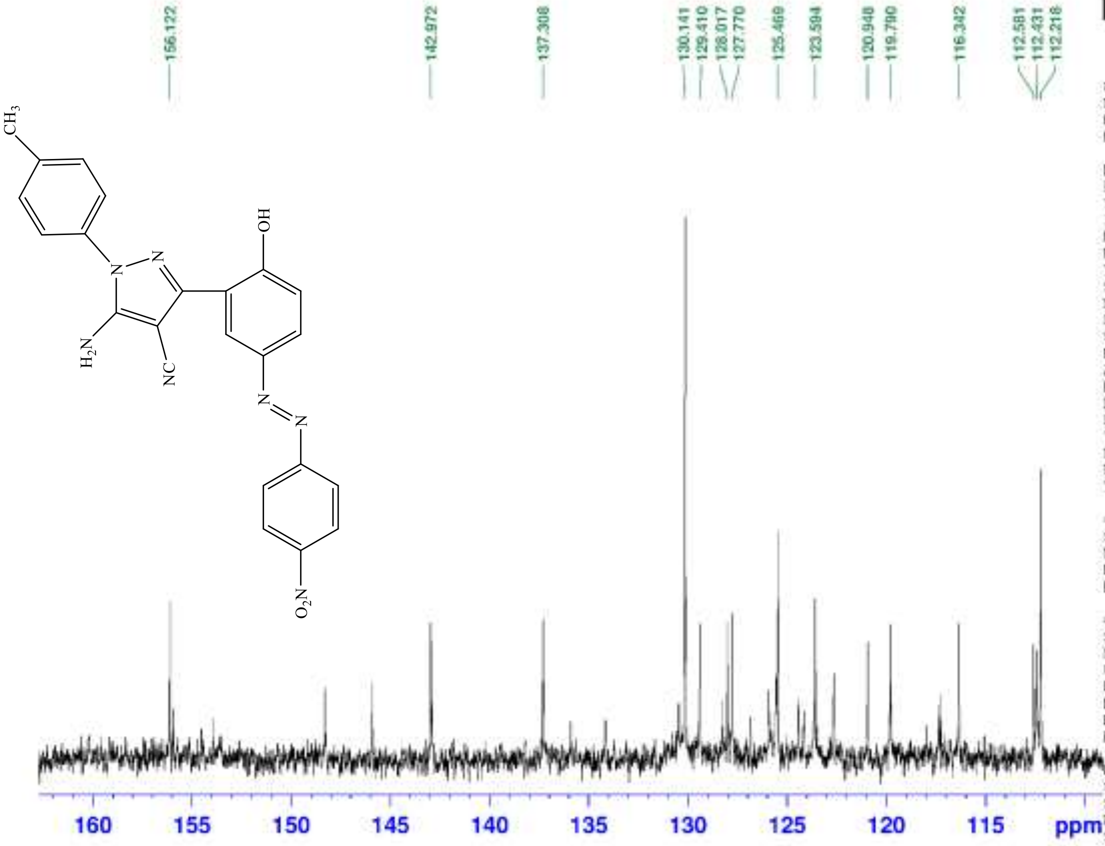

BRUKER

```
Current Data Parameters
NAME      May06-2018-nmr
EXPNO                 10
PROCNO                 1

F2 - Acquisition Parameters
Date_           20180506
Time               11.58
INSTRUM            spect
PROBHD   5 mm PABBO BB-
PULPROG          zgpg30
TD                65536
SOLVENT            DMSO
NS                  512
DS                    4
SWH           18115.941 Hz
FIDRES         0.276427 Hz
AQ            1.8087935 sec
RG                  202
DW               27.600 usec
DE                 6.50 usec
TE                296.9 K
D1           2.00000000 sec
D11          0.03000000 sec
TD0                   1

======== CHANNEL f1 ========
SFO1         75.6462982 MHz
NUC1                13C
P1                10.00 usec
PLW1        30.00000000 W

======== CHANNEL f2 ========
SFO2        300.8112032 MHz
NUC2                 1H
CPDPRG[2         waltz16
PCPD2             90.00 usec
PLW2         6.40000010 W
PLW12        0.17778000 W
PLW13        0.14399999 W

F2 - Processing parameters
SI                32768
SF           75.6387350 MHz
WDW                  EM
SSB      0
LB                 1.00 Hz
GB       0
PC                 1.40
```

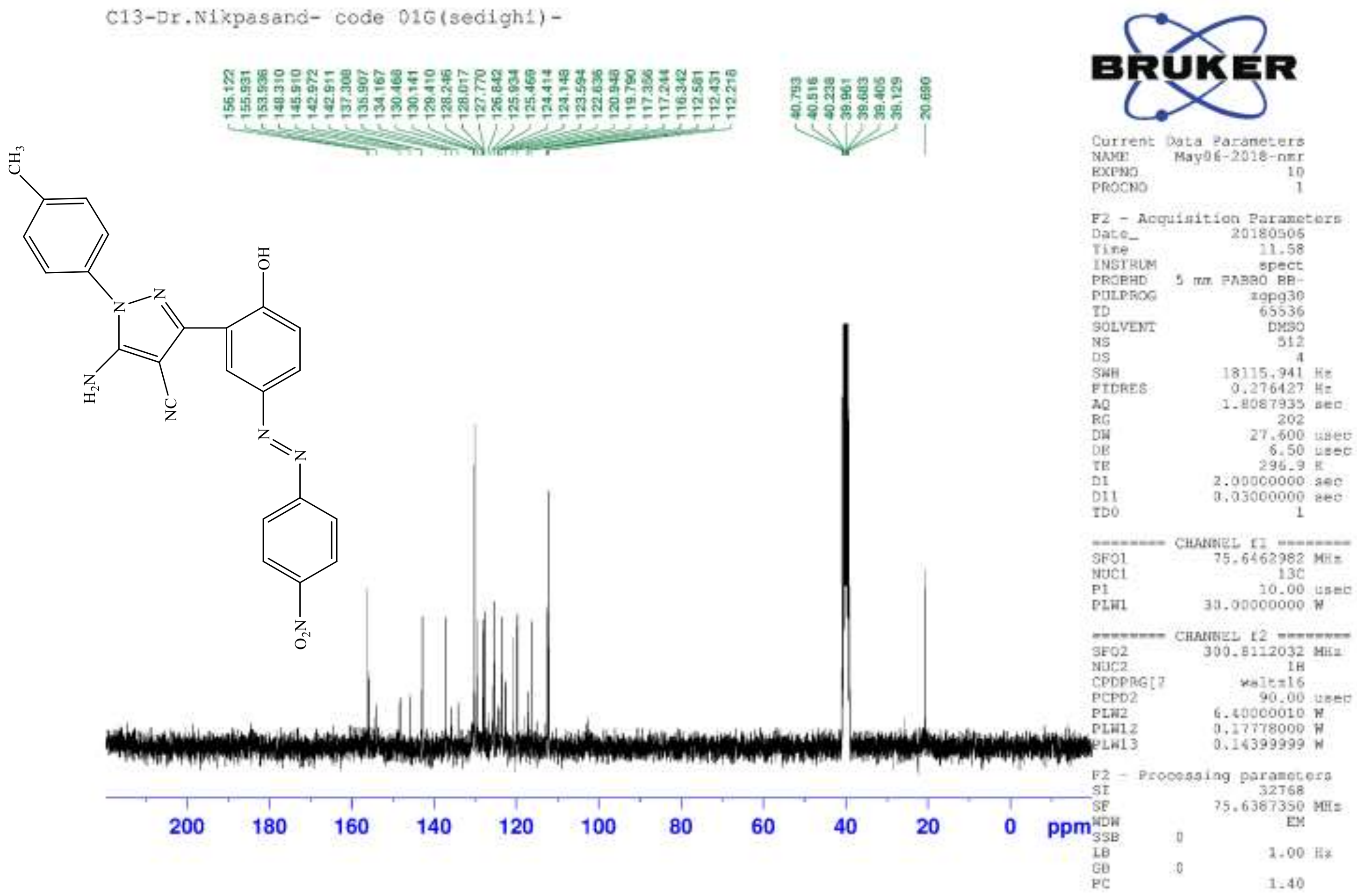
C13-Dr.Nikpasand- code 01G(sedighi)-
156.122
155.931
153.936
148.310
145.910
142.972
142.911
137.308
135.907
134.167
130.468
130.141
129.410
128.246
128.017
127.770
126.842
125.934
125.469
124.414
124.148
123.594
122.636
120.948
119.790
117.356
117.244
116.342
112.581
112.431
112.218
40.793
40.516
40.238
39.961
39.683
39.405
39.129
20.690
CH3
OH
N
N
H2N
NC
N
N
O2N
200
180
160
140
120
100
80
60
40
20
0
ppm
BRUKER
Current Data Parameters
NAME May06-2018-nmr
EXPNO 10
PROCNO 1
F2 - Acquisition Parameters
Date_ 20180506
Time 11.58
INSTRUM spect
PROBHD 5 mm PABBO BB-
PULPROG zgpg30
TD 65536
SOLVENT DMSO
NS 512
DS 4
SWH 18115.941 Hz
FIDRES 0.276427 Hz
AQ 1.8087935 sec
RG 202
DW 27.600 usec
DE 6.50 usec
TE 296.9 K
D1 2.00000000 sec
D11 0.03000000 sec
TD0 1
======== CHANNEL f1 ========
SFO1 75.6462982 MHz
NUC1 13C
P1 10.00 usec
PLW1 30.00000000 W
======== CHANNEL f2 ========
SFO2 300.8112032 MHz
NUC2 1H
CPDPRG[2 waltz16
PCPD2 90.00 usec
PLW2 6.40000010 W
PLW12 0.17778000 W
PLW13 0.14399999 W
F2 - Processing parameters
SI 32768
SF 75.6387350 MHz
WDW EM
SSB 0
LB 1.00 Hz
GB 0
PC 1.40

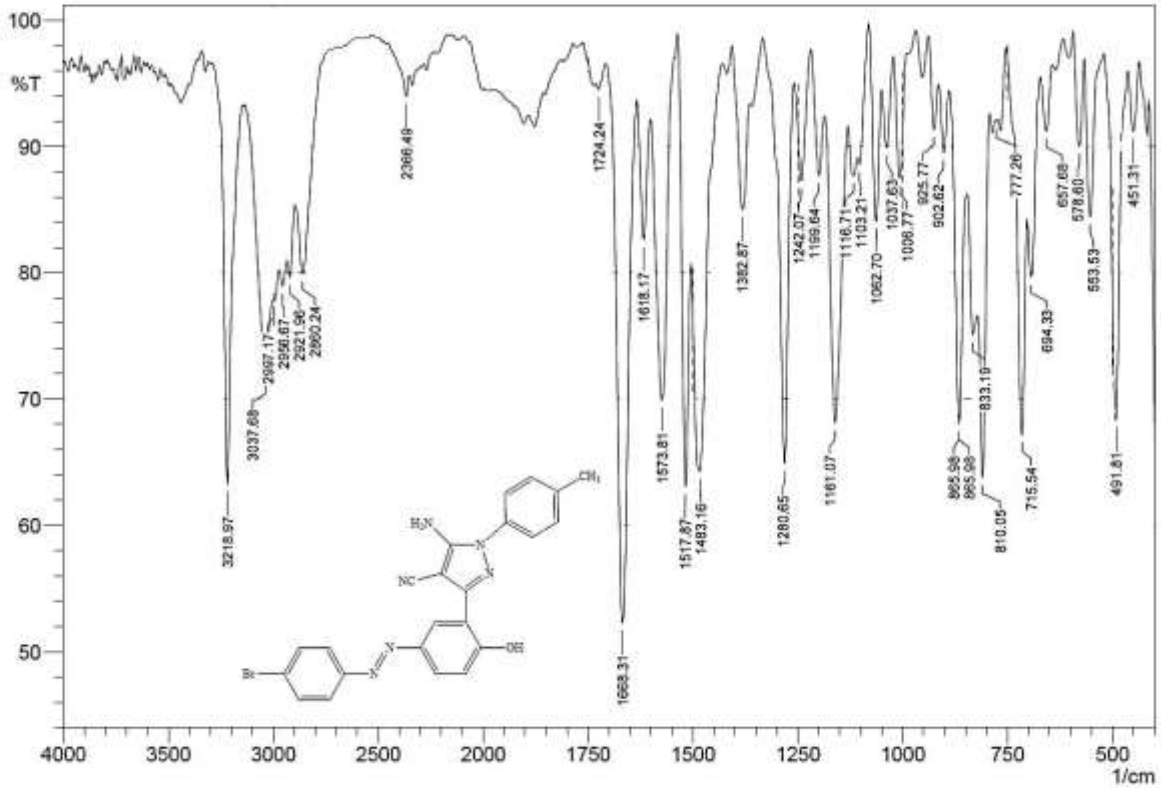
%T
100
90
80
70
60
50
4000
3500
3000
2500
2000
1750
1500
1250
1000
750
500
1/cm
3218.97
3037.68
2997.17
2956.67
2921.96
2860.24
2366.48
1724.24
1668.31
1618.17
1573.81
1517.87
1483.16
1382.87
1280.65
1242.07
1199.64
1161.07
1116.71
1103.21
1062.70
1037.63
1006.77
925.77
902.62
833.19
810.05
777.26
715.54
694.33
657.68
578.60
553.53
491.81
451.31
H2N
NC
OH
Br

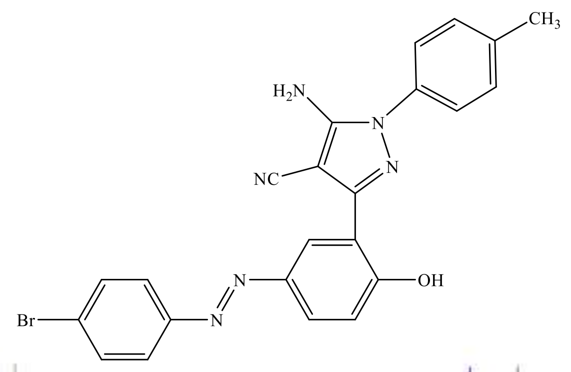
CH3
H2N
N
N
NC
N
N
OH
Br

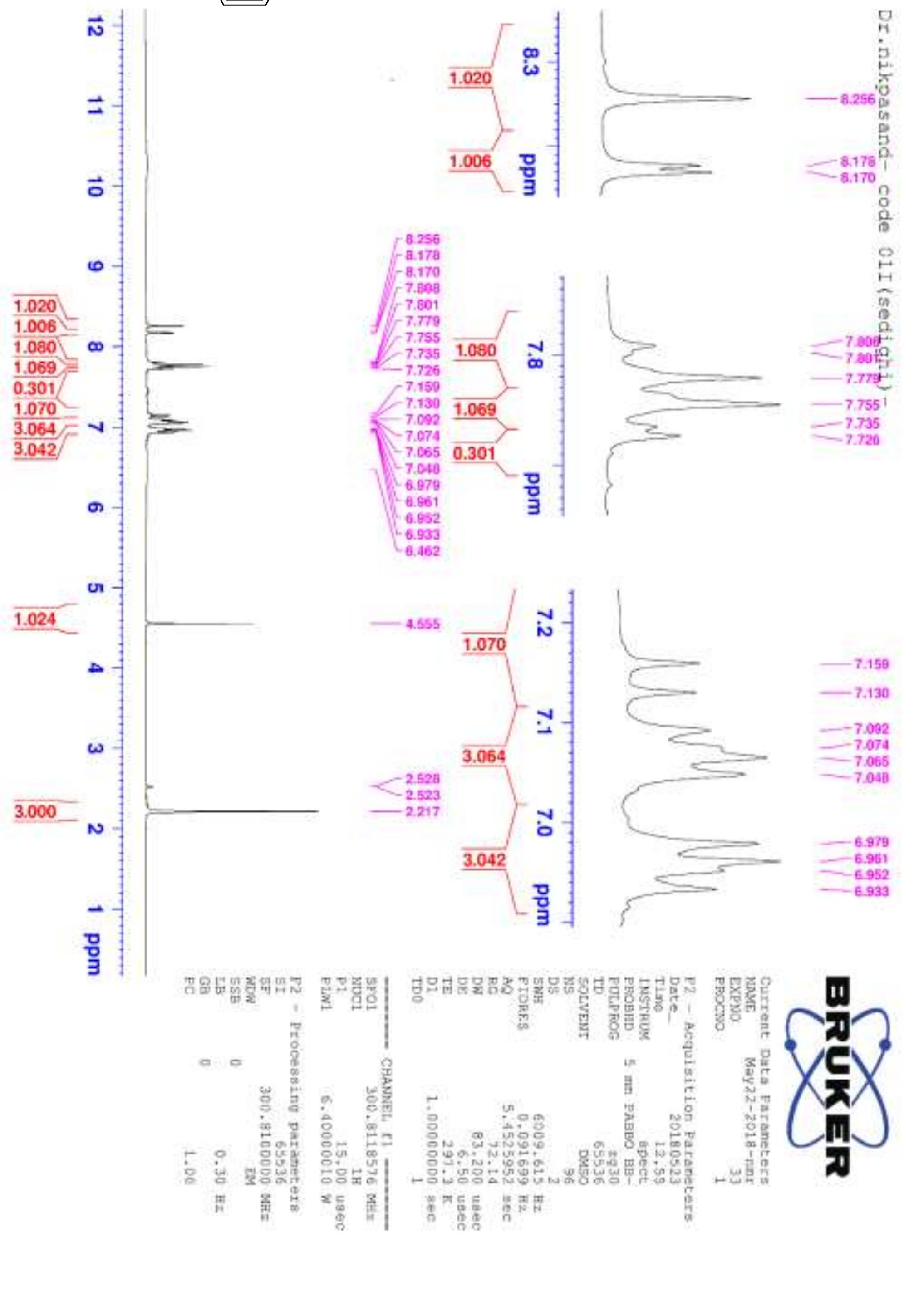

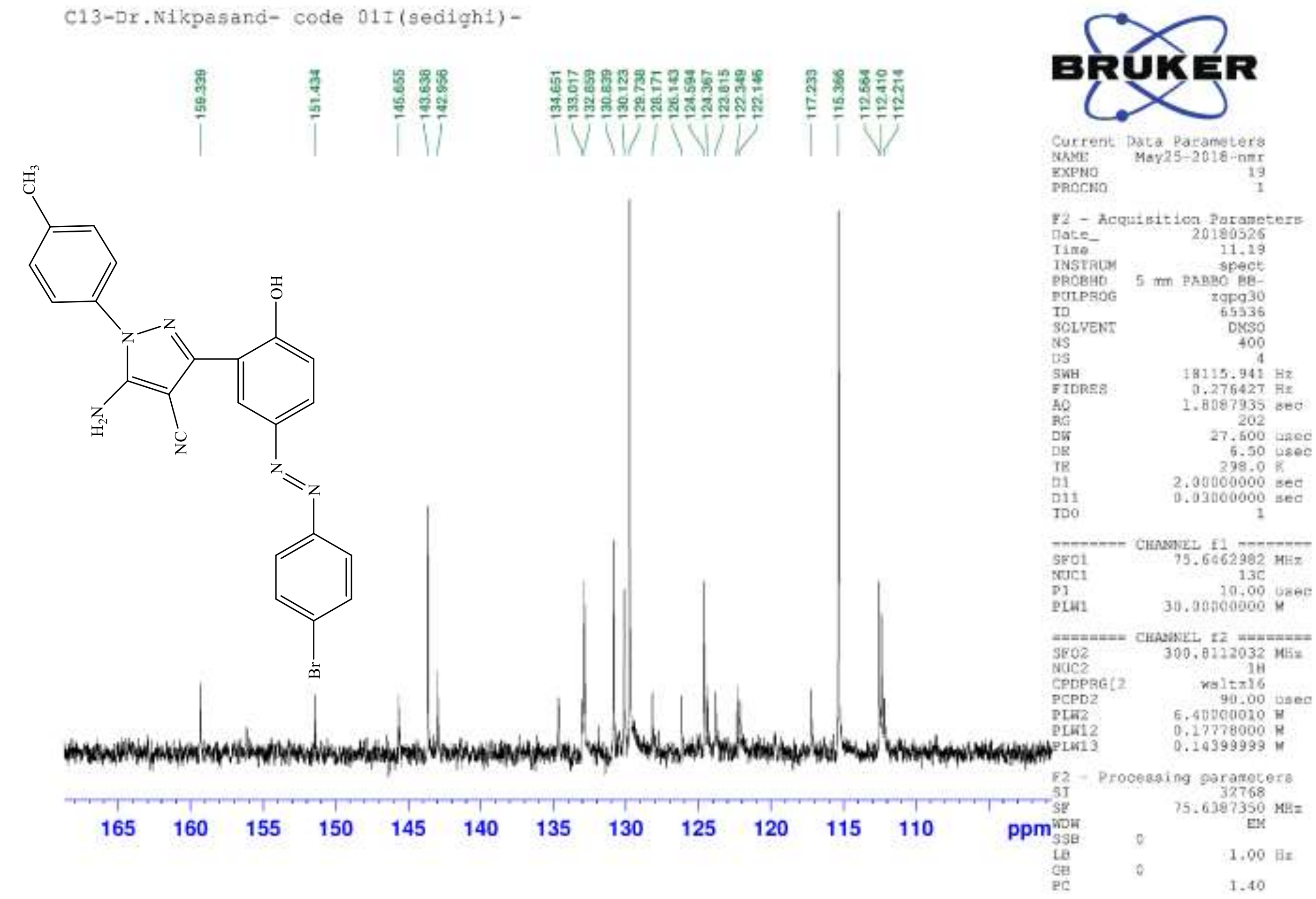
C13-Dr.Nikpasand- code 01I(sedighi)-
159.339
151.434
145.655
143.638
142.956
134.651
133.017
132.659
130.639
130.123
129.738
128.171
126.143
124.594
124.367
123.815
122.349
122.146
117.233
115.366
112.564
112.410
112.214
CH3
OH
N
N
H2N
NC
N
N
Br
165 160 155 150 145 140 135 130 125 120 115 110 ppm
BRUKER
Current Data Parameters
NAME May25-2018-nmr
EXPNO 19
PROCNO 1
F2 - Acquisition Parameters
Date_ 20180526
Time 11.19
INSTRUM spect
PROBHD 5 mm PABBO BB-
PULPROG zgpg30
TD 65536
SOLVENT DMSO
NS 400
DS 4
SWH 18115.941 Hz
FIDRES 0.276427 Hz
AQ 1.8087935 sec
RG 202
DW 27.600 usec
DE 6.50 usec
TE 298.0 K
D1 2.00000000 sec
D11 0.03000000 sec
TD0 1
======== CHANNEL f1 ========
SFO1 75.6462982 MHz
NUC1 13C
P1 10.00 usec
PLW1 30.00000000 W
======== CHANNEL f2 ========
SFO2 300.8112032 MHz
NUC2 1H
CPDPRG[2 waltz16
PCPD2 90.00 usec
PLW2 6.40000010 W
PLW12 0.17778000 W
PLW13 0.14399999 W
F2 - Processing parameters
SI 32768
SF 75.6387350 MHz
WDW EM
SSB 0
LB 1.00 Hz
GB 0
PC 1.40

C13-Dr.Nikpasand- code 01I(sedighi)-

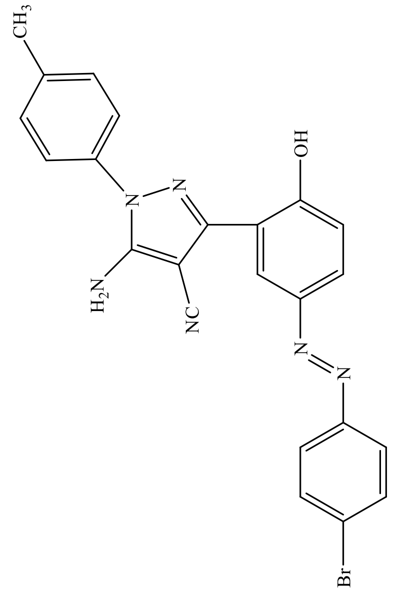

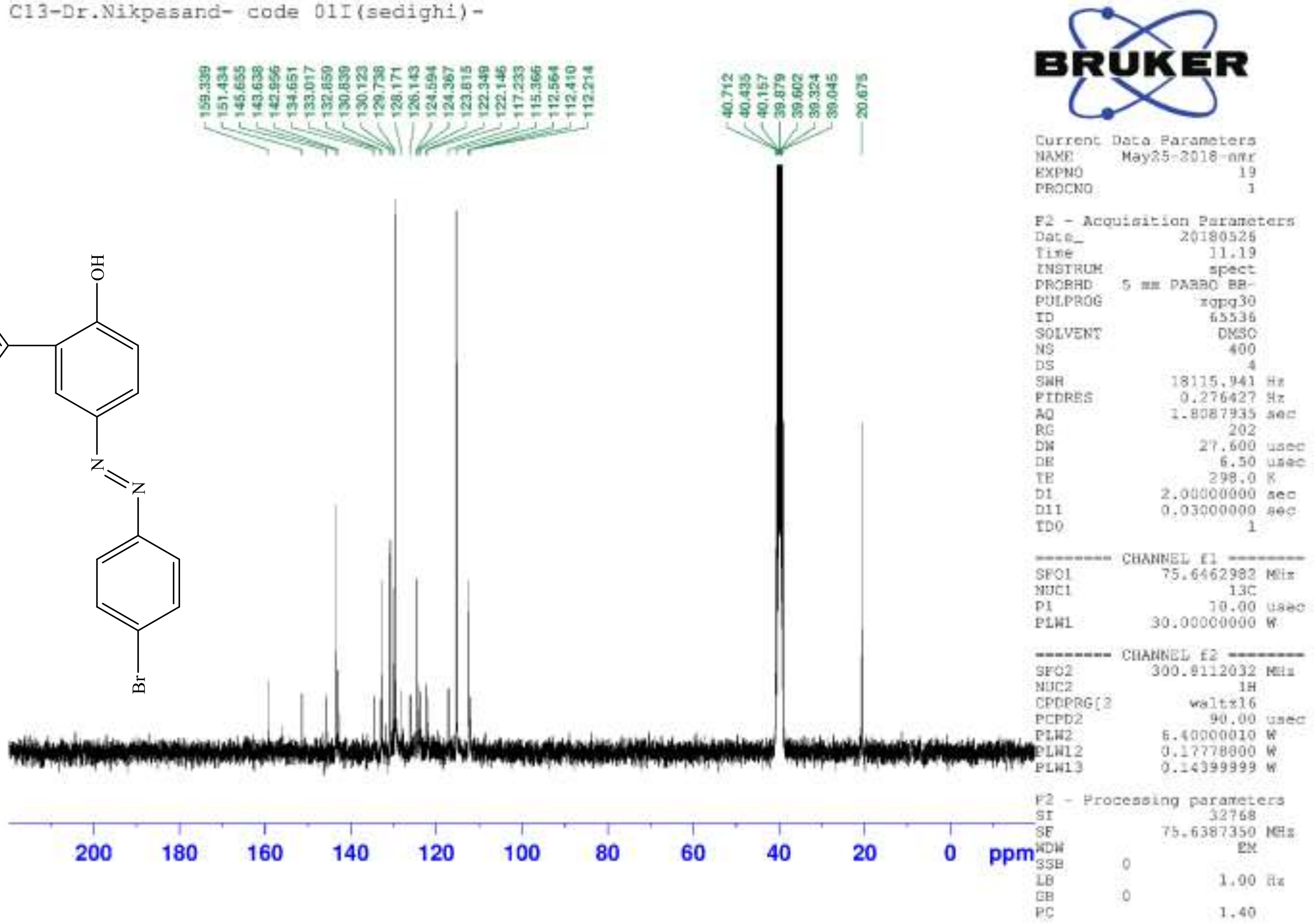

Supplement: Supplementary file 1 [file DataSheet1.PDF]
